# Histopathological predictors of lymph node metastasis in oral cavity squamous cell carcinoma: a systematic review and meta-analysis

**DOI:** 10.3389/fonc.2024.1401211

**Published:** 2024-05-14

**Authors:** Sadiq Alqutub, Abdulsalam Alqutub, Ahmed Bakhshwin, Zainab Mofti, Sulafa Alqutub, Ameera A. Alkhamesi, Mohammed A. Nujoom, Almoaidbellah Rammal, Mazin Merdad, Hani Z. Marzouki

**Affiliations:** ^1^ Department of Pathology and Laboratory Medicine, King Abdulaziz University, Jeddah, Saudi Arabia; ^2^ Department of Otolaryngology-Head and Neck Surgery, King Abdulaziz University, Jeddah, Saudi Arabia; ^3^ Department of Family and Community Medicine, King Abdulaziz University, Jeddah, Saudi Arabia; ^4^ Department of Family and Community Medicine, University of Jeddah, Jeddah, Saudi Arabia

**Keywords:** predictors, squamous cell carcinoma of head and neck, oral cancer, lymphatic metastasis, neck dissection, overtreatment, standard of care

## Abstract

**Objectives:**

Lymph node metastasis (LNM) is the most significant parameter affecting overall survival in patients with oral cavity squamous cell carcinomas (OCSCC). Elective neck dissection (END) is the standard of care in the early management of OCSCC with a depth of invasion (DOI) greater than 2-4 mm. However, most patients show no LNM in the final pathologic report, indicating overtreatment. Thus, more detailed indicators are needed to predict LNM in patients with OCSCC. In this study, we critically evaluate the existing literature about the risk of different histological parameters in estimating LNM.

**Methods:**

A systematic review was conducted using PRISMA guidelines. PubMed, Web of Science, Cochrane, and Scopus were searched from inception to December 2023 to collect all relevant studies. Eligibility screening of records was performed, and data extraction from the selected studies was carried out independently. Inclusion in our systematic review necessitated the following prerequisites: Involvement of patients diagnosed with OCSCC, and examination of histological parameters related to lymph node metastasis in these studies. Exclusion criteria included animal studies, non-English articles, non-availability of full text, and unpublished data.

**Results:**

We included 217 studies in our systematic review, of which 142 were eligible for the meta-analysis. DOI exceeding 4 mm exhibited higher risk for LNM [Risk ratio (RR) 2.18 (1.91-2.48), p<0.00001], as did perineural invasion (PNI) [RR 2.04 (1.77-2.34), p<0.00001], poorly differentiated tumors [RR 1.97 (1.61-2.42), p<0.00001], lymphovascular invasion (LVI) [RR 2.43 (2.12-2.78), p<0.00001], groups and single pattern of invasion [RR 2.47 (2.11-2.89), p<0.00001], high tumor budding [RR 2.65 (1.99-3.52), p<0.00001], tumor size over 4 cm [RR 1.76 (1.43-2.18), p<0.00001], tumor thickness beyond 4 mm [RR 2.72 (1.91-3.87), p<0.00001], involved or close margin [RR 1.73 (1.29-2.33), p = 0.0003], and T3 and T4 disease [RR 1.98 (1.62-2.41), p <0.00001].

**Conclusion:**

Our results confirm the potential usefulness of many histopathological features in predicting LNM and highlight the promising results of others. Many of these parameters are not routinely incorporated into pathologic reports. Future studies must focus on applying these parameters to examine their validity in predicting the need for elective neck treatment.

## 1 Introduction

Oral cancer, primarily manifested as oral cavity squamous cell carcinomas (OCSCC), constitutes more than 90% of malignant cases within the oral cavity. This form of cancer significantly contributes to global cancer-related mortality, leading to approximately 177,000 deaths annually (1). Defined by its aggressive nature, OCSCC presents a formidable clinical scenario, exhibiting a 5-year overall survival rate of roughly 50%, a figure that declines to below 30% in advanced stages of the disease (2). Cervical lymph node involvement has been linked to a 50% reduction in overall survival and a higher incidence of distant metastasis, making it the most significant parameter affecting overall survival in patients with OCSCC (3, 4). Clinical examination and imaging modalities, including positron emission tomography, computed tomography, ultrasonography, and magnetic resonance imaging, have been used to detect nodal metastases. However, none of these methods can identify micrometastases in cervical nodes, and the sensitivity of these modalities in identifying preoperative nodal metastasis is only 70% (5, 6). In recent studies, sentinel lymph node biopsy has shown promising results in detecting occult lymph node metastasis (LNM). However, due to operator sensitivity and sampling errors, a wide range of false-negative outcomes has been reported (from 2.56% to 36%), making its reliability subject to debate in OCSCC (7–11). Elective neck dissection (END) is the current standard of care in the early management of OCSCC with a depth of invasion (DOI) greater than 2-4 mm (12). Many prospective studies and meta-analyses conducted in the past few years have indicated that patients undergoing END may have favorable survival outcomes (12, 13). Still, less than one-third of these individuals were found to have occult lymph node metastases, indicating that roughly 70% of them had unnecessary surgery (12).

Two key histological features have been added to the 8th iteration of the TNM staging system published by the American Joint Committee on Cancer (AJCC) (14). These are DOI, denoting the extent of tumor invasion from the basement membrane to the deepest point of invasion, and extranodal extension (ENE), signifying the spread of tumor cells beyond the lymph nodal capsule. Clinical and/or imaging assessments can ascertain both features, but histological evaluation remains indispensable, particularly in non-straightforward or ambiguous cases (4). Numerous studies revealed the prognostic significance of other histopathological features, such as tumor thickness, pattern of invasion, lymphovascular invasion (LVI), surgical margins, perineural invasion (PNI), tumor budding, and tumor-stroma ratio, which have been described more recently (15). Although these parameters have been studied for their prognostic impact, no large randomized controlled trials or meta-analyses have investigated their potential to predict LNM. More detailed indicators are needed to complement the TNM staging in determining which patients can benefit from END (15). Ideally, neck dissection would be performed on patients with tumors at high risk of LNM; the remainder could be treated with local tumor excision and close clinicoradiological monitoring, minimizing the rates of unnecessary surgeries. This paper systematically reviews the literature, focusing on the histological characteristics of primary OCSCC that may serve as potential predictors for the presence of lymph node metastases. This information holds the potential to aid in the strategic triaging of patients, distinguishing those who may benefit from additional surgeries from those in whom such procedures might be avoidable.

### 1.1 Methods

The methodology employed in this research study aligned with the recommendations delineated in the Cochrane Handbook for Systematic Reviews of Interventions. Additionally, the documentation of this study followed the guidelines established by the Preferred Reporting Items for Systematic Reviews and Meta-analyses (PRISMA) (13, 14).

### 1.2 Literature search

To compile pertinent literature, a comprehensive exploration of various databases—PubMed, Web of Science, Cochrane, and Scopus—was conducted from their inception until December 2023. A manual search of eligible articles and prior meta-analyses within the field was done to include any missed citations. The following search terms were used: (((“Oral squamous cell carcinoma” OR “ OSCC” OR “oral cancer” OR “oral carcinoma”) AND (prognosis OR predict* OR survival OR recurrence OR mortality OR metastasis)) AND (“depth of invasion” OR “invasion” OR “tumor thickness” OR “tumor length” OR “budding” OR “pattern of invasion” OR “tumor invasion” OR “tumor infiltration” OR “tumor island” OR “grade” OR “grading” OR “lymphovascular invasion” OR “lymphoid response” OR “perineural” OR “tumor size” OR “lympho-vascular invasion”))).

### 1.3 Eligibility criteria

The identified references underwent individual screening to evaluate their eligibility against pre-established criteria. Inclusion in our systematic review necessitated compliance with the following prerequisites: 1) Involvement of patients diagnosed with OCSCC and 2) Examination of histological parameters related to lymph node metastasis in the study. Numerous studies were omitted for specific reasons, such as 1) animal studies, 2) non-English articles, 3) non-availability of full text, and 4) unpublished data. This review focused on histomorphological parameters diagnosed in hematoxylin and eosin (H&E)-stained slides. Studies that only concentrated on molecular parameters as predictors for LNM were excluded.

### 1.4 Data gathering

Data collection was executed using an offline data extraction template, systematically capturing pertinent details from each study. The extracted data encompassed vital elements such as the primary author’s name and publication year, participant numbers, study location, participants’ age, and gender distribution. It also evaluated histological parameters, inclusion criteria, and the conclusion drawn from each study. One reviewer collected all data, which were then cross-checked by another.

### 1.5 Risk of bias assessment

The quality assessment of the cohort studies included in our review was conducted by the National Institutes of Health (NIH) (16). Studies underwent an assessment where scores were assigned, determining their quality. The quality of the retrieved RCT was assessed using the Cochrane Risk of Bias Tool 1 (ROB1) (17). The study evaluated bias across various domains, including sequence generation, allocation concealment, blinding of participants and outcome assessors, incomplete outcome data, selective outcome reporting, and other potential sources of bias. Discrepancies in assessment were resolved through discussions between investigators or a third assessor to maintain accuracy and consistency.

### 1.6 Assessing the risk of bias across studies

We used the funnel plot to investigate publication bias and minor study effects. We plotted the standard error *vs*. risk ratio for the included studies in each assessed parameter and then judged it using visual inspection.

### 1.7 Data synthesis

We calculated risk ratios (RR) with a 95% confidence interval (CI) to evaluate dichotomous outcomes between the compared groups, employing the Mantel-Haenszel method. Initially, a fixed-effect model was applied for homogeneous studies, allowing for the pooling of effect estimates. However, a random-effects model was utilized in instances of observed heterogeneity among the studies. Evaluation of statistical heterogeneity involved the I^2^ statistic and the Chi^2^ test, with a significance threshold of p < 0.10 indicating heterogeneity and an I^2^≥50% denoting substantial heterogeneity. All statistical analyses were conducted using Review Manager software [(RevMan), Version 5.4, The Cochrane Collaboration, 2020].

## 2 Results

### 2.1 Literature search results

**Figure 1:**
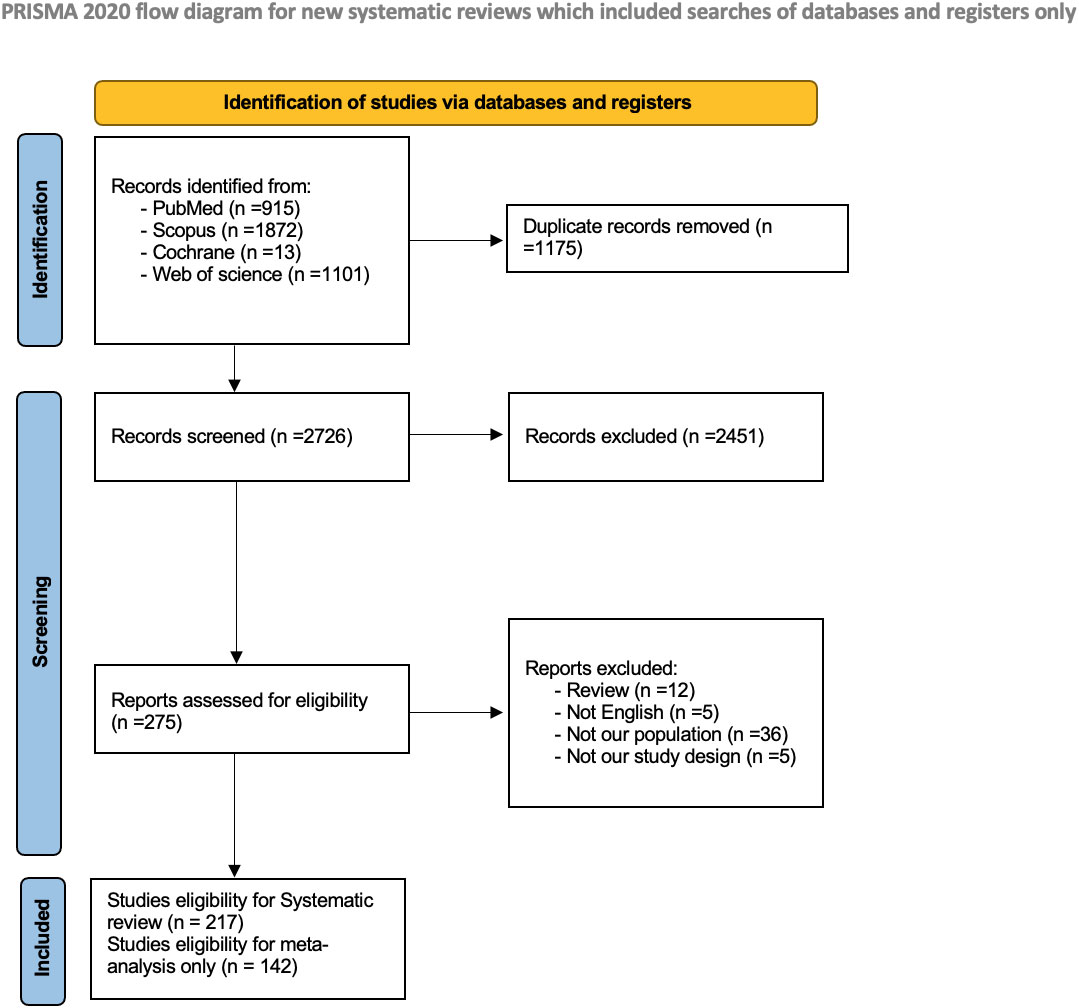
PRISMA flow chart.

The preliminary search across four databases yielded 3901 studies. After removing duplicate entries, 2726 distinct articles remained for further scrutiny. The screening process entailed an assessment of titles and abstracts, identifying 275 studies deemed potentially pertinent for full-text evaluation. Fifty-eight studies were excluded based on pre-established criteria. Eventually, 217 studies met the stipulated inclusion criteria for the systematic review, with 142 studies eligible for inclusion in the subsequent meta-analysis. The PRISMA flow diagram is shown in **Figure 1**.

### 2.2 Characteristics of the included studies

Two hundred and seventeen studies were included, encompassing 49999 patients across 19 countries. Of them, 199 studies (91.7%) were retrospective cohort, 15 (6.9%) were prospective cohort, two (0.9%) were cross-sectional, and one study (0.5%) was an RCT. Perineural invasion, DOI, degree of differentiation, and lympho-vascular invasion were the most studied histological parameters in our included studies, with the following number of studies assessed them: 69 (31.7%), 85 (39.2%), 57 (26.2%), and 51(23.5%), respectively. A summary of the characteristics of the included studies is provided in **Supplementary Table 1**.

### 2.3 Risk of bias assessment

In our analysis of the included RCT by Yang et al. (18), most domains exhibited a low risk of bias. However, there was uncertainty regarding the risk of performance bias. ROB1 assessment is provided in **Supplementary Table 2**. Additionally, most of our cohort studies showed fair quality on the NIH tool, scoring between 9 and 11. Specifically, 75 studies (34.7%) were of good quality, 138 studies (63.8%) were fair, and three studies (1.5%) were of poor quality. Most studies did not clearly state blinding status among investigators and participants. The NIH tool judgment tables are provided in **Supplementary Table 3**.

### 2.4 Publication bias assessment

By visual inspection, funnel plots showed asymmetry, suggesting a possible publication bias in the following assessed parameters: depth of invasion, degree of differentiation, pattern of invasion, lymphovascular invasion, tumor budding, tumor thickness, tumor grade, and tumor size. The rest of the parameters showed a symmetric distribution around the pooled estimate **Supplementary Figures 1**-**12**.

### 2.5 Outcomes

#### 2.5.1 DOI (mm)

**Figure 2:**
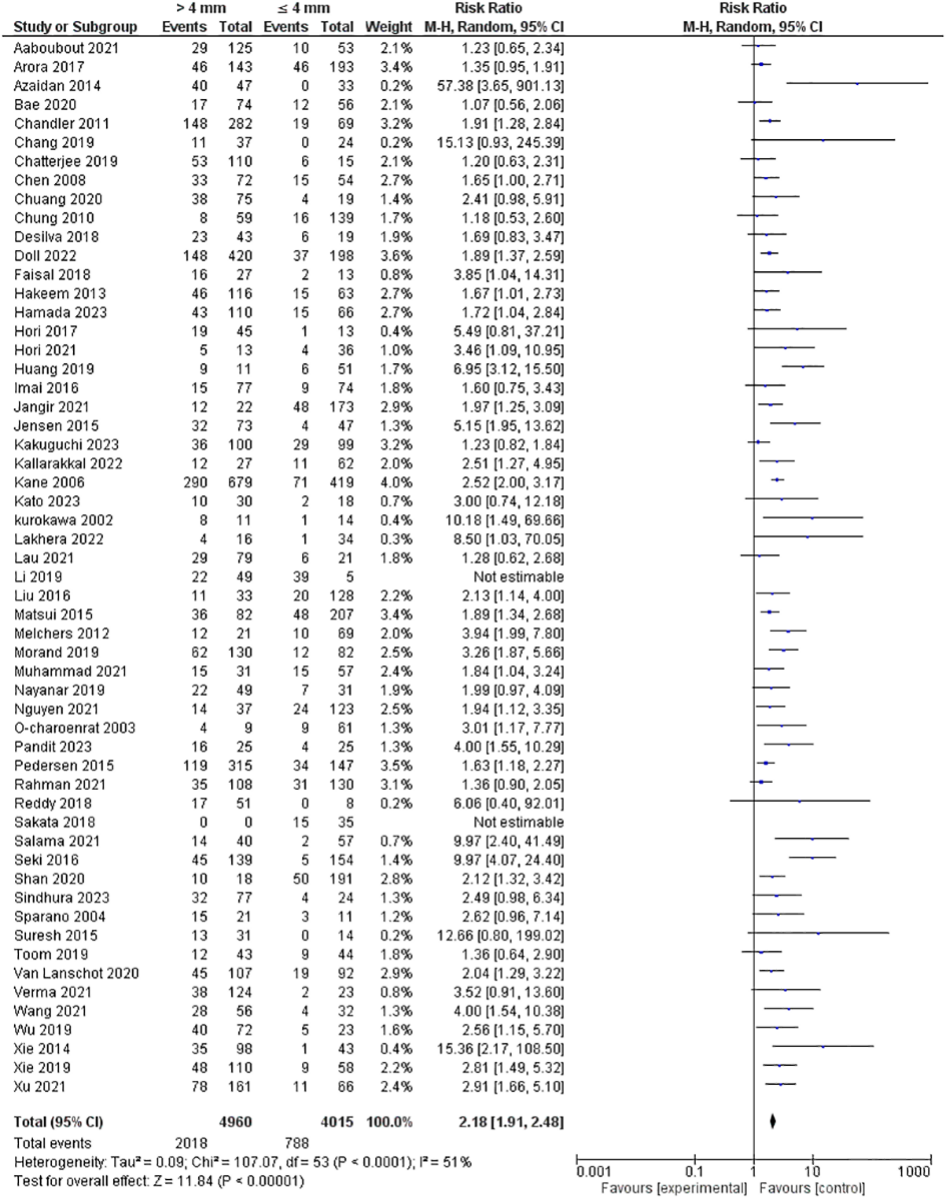
Risk of LNM according to the depth of invasion.

Tumor depth of invasion was evaluated in 56 studies encompassing 8975 patients. Our pooled RR showed a depth of more than 4 mm invasion had a higher risk of lymph node metastasis; 2.18 [1.91-2.48], p <0.00001. However, heterogeneity was observed in our pooled analysis; I^2^ and Chi^2^-p = (51%, <0.0001). **Figure 2** illustrates the forest plot for the DOI outcome.

#### 2.5.2 PNI

**Figure 3:**
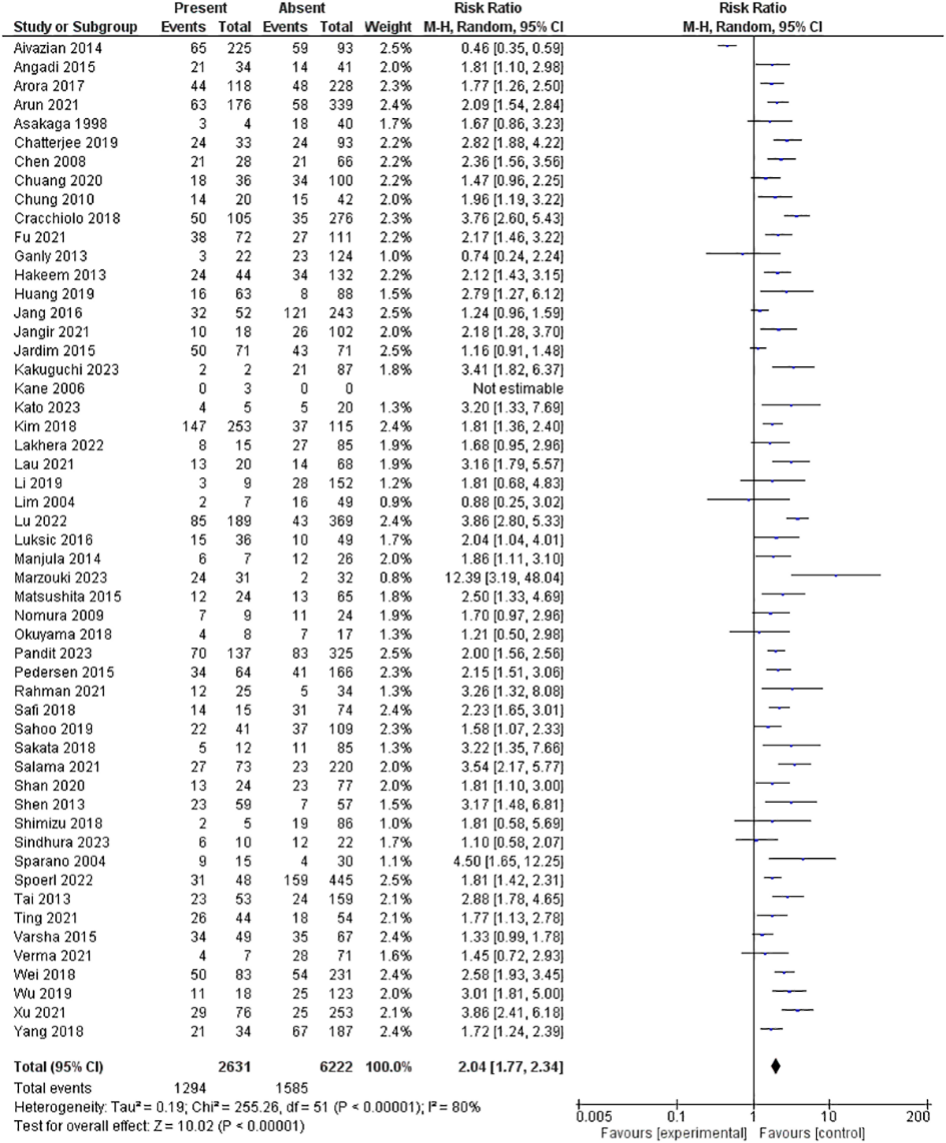
Risk of LNM according to the perineural invasion.

The PNI was evaluated in 53 studies encompassing 8853 patients. Our pooled RR showed the presence of PNI had a significantly higher risk for lymph node metastasis; 2.04 [1.77-2.34], p <0.00001. However, heterogeneity was observed in our pooled analysis; I^2^ and Chi^2^-p = (80%, <0.00001). **Figure 3** illustrates the forest plot for the PNI outcome.

#### 2.5.3 Degree of differentiation

**Figure 4:**
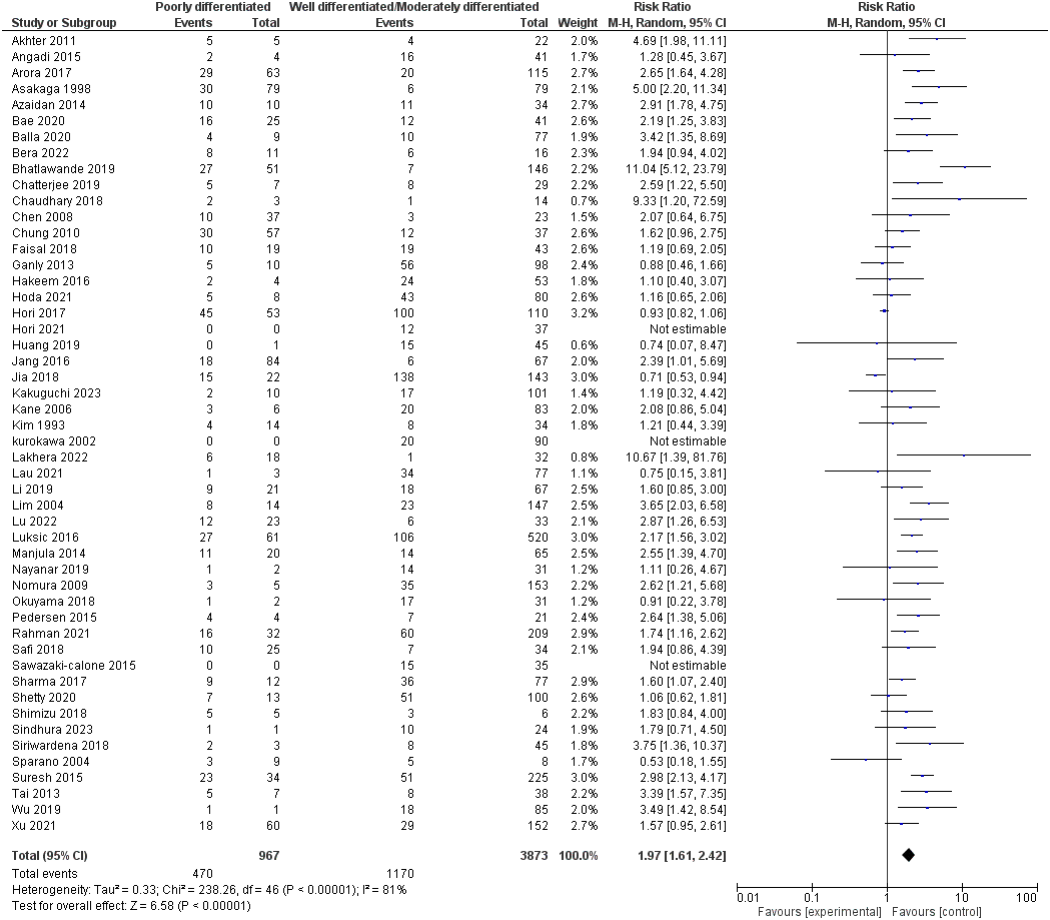
Risk of LNM according to the degree of differentiation.

Fifty studies assessed the degree of differentiation of oral squamous cell carcinoma, including 4840 patients. Our pooled RR showed that the poorly differentiated group had a higher risk of lymph node metastasis: 1.97 [1.61-2.42], p <0.00001. However, heterogeneity was observed in our pooled analysis; I^2^ and Chi^2^-p = (81%, <0.00001). **Figure 4** demonstrates the forest plot for the degree of differentiation outcome.

#### 2.5.4 LVI

**Figure 5:**
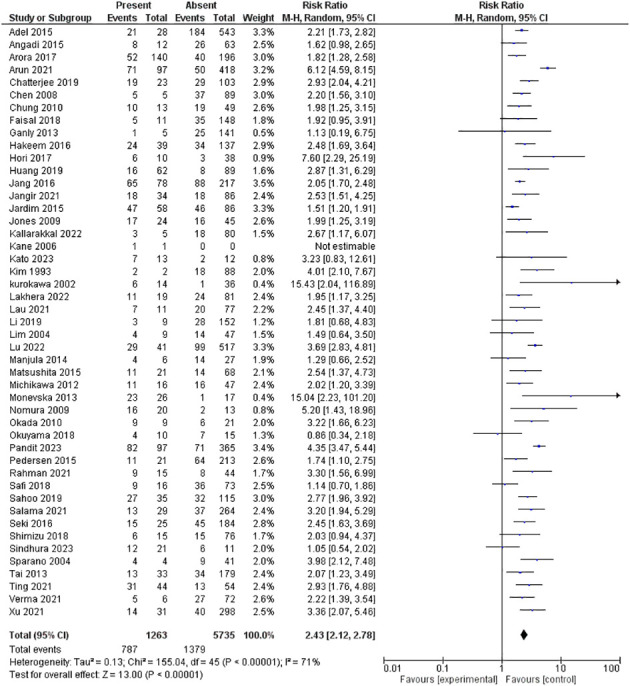
Risk of LNM according to the lympho-vascular invasion.

Forty-seven studies assessed LVI, including 6,998 patients. The pooled RR showed that the presence of LVI had a significantly higher risk for lymph node metastasis; 2.43 [2.12-2.78], p <0.00001. However, heterogeneity was observed in the pooled analysis; I^2^ and Chi^2^-p = (71%, <0.00001). **Figure 5** illustrates the forest plot LVI outcome.

#### 2.5.5 Lymphoplasmacytic infiltration

The lymphoplasmacytic infiltration was evaluated in 9 studies, including 881 patients. Our pooled RR showed no significant difference between the two groups regarding lymph node metastasis: 0.8 [0.5-1.28], p = 0.36. Heterogeneity was observed among pooled studies; I^2^ and Chi^2^-p = (72%, 0.00009). **Supplementary Figure 13** shows the forest plot for the lymphoplasmocytic infiltration outcome.

#### 2.5.6 Pattern of invasion

The pattern of invasion was evaluated in 21 studies encompassing 2862 patients. Pooled RR showed that groups and single invasion patterns had a significantly higher risk for lymph node metastasis than tumors with pushing borders and tumors advancing in cord, band, or strand patterns of invasion; 2.47 [2.11-2.89], p <0.00001. Pooled studies were homogeneous; I^2^ and Chi^2^-p = (45%, 0.02). **Supplementary Figure 14** demonstrates the forest plot for a pattern of invasion outcome.

#### 2.5.7 Margin status

According to The National Comprehensive Cancer Network (NCCN), a margin ≥5 mm is classified as a clear margin, a margin of 1-5 mm is considered close, and a margin <1 mm is regarded as involved (19). Nine studies encompassing 1970 patients evaluated the margin status. The pooled RR showed that the group with an involved or close margin carried a significantly higher risk of lymph node metastasis: 1.73 [1.29-2.33], p = 0.0003. Pooled studies were homogeneous; I^2^ and Chi^2^-p = (0%, 0.48). **Supplementary Figure 15** shows the forest plot for the margin status outcome.

#### 2.5.8 Tumor budding

Twenty-five studies evaluated tumor budding outcomes, including 3042 patients. Our pooled RR showed that high tumor budding had a significantly higher risk for lymph node metastasis: 2.65 [1.99-3.52], p <0.00001. Heterogeneity was observed among pooled studies; I^2^ and Chi^2^-p = (81%, <0.00001). **Supplementary Figure 16** shows the forest plots for tumor budding outcome.

#### 2.5.9 Tumor size (cm)

Tumor size was evaluated in 10 studies encompassing 2650 patients. The pooled RR showed that tumor size over 4 cm had a significantly higher risk for lymph node metastasis: 1.76 [1.43-2.18], p <0.00001. Heterogeneity was observed among pooled studies; I^2^ and Chi^2^-p = (56%, 0.01). **Supplementary Figure 17** illustrates the forest plot for tumor size outcome.

#### 2.5.10 Tumor thickness (mm)

Twenty-three studies assessed the tumor thickness, encompassing 3022 patients. The pooled RR showed that tumor thickness of more than 4 mm had a higher risk for lymph node metastasis; 2.72 [1.91-3.87], p <0.00001. Heterogeneity was observed among pooled studies; I^2^ and Chi^2^-p = (72%, <0.00001). **Supplementary Figure 18** demonstrates the forest plot for tumor thickness outcome.

#### 2.5.11 Tumor grade

Grading was assessed in 13 studies encompassing 3678 patients. The pooled RR showed that tumors G3 and G4 had a higher risk for lymph node metastasis; 1.84 [1.49-2.26], p <0.00001. Heterogeneity was observed among pooled studies; I^2^ and Chi^2^-p = (43%, 0.05). **Supplementary Figure 19** illustrates the forest plot for tumor grade outcome.

#### 2.5.12 Growth pattern

Five studies assessed the growth pattern, including 468 patients. The pooled RR revealed no difference between exophytic and endophytic growth patterns regarding lymph node metastasis: 0.82 [0.48-1.4], p = 0.46. Heterogeneity was observed among our pooled studies: I^2^ and Chi^2^-p = (74%, 0.004). **Supplementary Figure 20** shows the forest plot for growth pattern outcome.

#### 2.5.13 Tumor stage

Tumor stage was assessed by TNM staging in 30 studies with 5258 patients. Pooled RR showed that tumor stage T3 and T4 had a significantly higher risk for lymph node metastasis; 1.98 [1.62-2.41], p <0.00001. Pooled studies showed heterogeneity with I^2^ and Chi^2^-p = (81%, <0.00001). However, T1, in comparison with T2, was assessed in 48 studies including 5792 patients, the pooled RR showed T2 had a significantly higher risk [1.74 (1.51, 1.99), p <0.00001]. Heterogeneity was observed among these pooled studies; I^2^ and Chi^2^-p = (44%, 0.0009). Also, T3 was compared to T4 in 26 studies encompassing 1765 patients. The pooled RR showed no significant difference among the two groups regarding lymph node metastasis [1.00 (0.85, 1.17), p = 0.96]; pooled studies showed heterogeneity; I^2^ and Chi^2^-p = (49%, 0.003). **Supplementary Figures 21**-**23** illustrate the forest plots for the tumor stage outcomes.

## 3 Discussion

A perfect biomarker should possess specificity, measurability, significance, and, ideally, accessibility and affordability. While OCSCC continues to rely on traditional TNM clinical staging for therapeutic strategies and prognostic determinations. Certain tumors have seen rapid advancements in this domain. For instance, tumor budding, lymphovascular invasion, and degree of differentiation are independent predictors of LNM in colorectal cancer (20–22). The TNM systems’ lack of specificity leads to erratic insights into the disease’s biology (15). Consequently, an imperative need arises for novel biomarkers to supplement the TNM system. These new biomarkers would aid in predicting treatment responses accurately and forecasting prognosis, thereby filling crucial gaps in current diagnostic and prognostic protocols (15).

Our meta-analysis investigated diverse factors influencing LNM risk in OCSCC patients. PNI, poorly differentiated tumors, specific invasion patterns (groups and single), LVI, high tumor budding, increased tumor size (> 4 cm), greater tumor thickness (> 4 mm), higher tumor grades (G3 and G4), higher tumor stages (T3 and 4 *vs*. T1 and 2), and depth of invasion (> 4 mm) all exhibited significantly elevated risks for LNM. Conversely, lymphoplasmacytic infiltration, growth patterns (exophytic *vs*. endophytic), and certain tumor stages (T4 *vs*. T3) did not showcase substantial variations in LNM risk.

In the meta-analysis conducted by Dolens et al., the confirmation of the predictive potential of various factors in OCSCC was noteworthy. Parameters such as extra-nodal extension, DOI, LVI, PNI, and involvement of surgical margins exhibited promising results in predicting poorer survival outcomes (15). Additionally, associations suggesting increased risk for poor survival were observed for patterns of invasion and tumor thickness despite the limited number of studies exploring these factors. Ironically, albeit based on a small number of studies, tumor budding, and tumor-stroma ratios also displayed clinical significance in predicting survival among patients with OCSCC (15). However, their study focused on survival outcomes rather than on risks for LNM. By prioritizing survival outcomes over a direct investigation into LNM, the study might lack detailed insights into the specific mechanisms or factors solely related to lymph node involvement. This could hinder the ability to draw precise conclusions about the applicability of different histological parameters in detecting OCSCC in its early stages.

Regarding DOI, the variability in clinical decision-making associated with it arises from its interchangeable usage with tumor thickness across various studies (23–29). It is essential, therefore, to understand the difference between tumor thickness and DOI. The 8th edition of the AJCC guidelines defined DOI as the distance measured between the basal membrane of normal adjacent mucosa and the deepest point of tumor invasion, focusing on the endophytic component of the tumor (30). In contrast, tumor thickness accounts for the tumor’s vertical bulk, consisting of the endophytic and exophytic components (4). This clarification aids in establishing a standardized understanding of DOI, addressing the ambiguity caused by its interchangeability with tumor thickness in various research contexts. Consequently, numerous studies have become outdated, and those published after the 8th edition of the AJCC exhibit considerable disparities (13, 23, 26, 31, 32). Several of these newer studies fail to validate the established DOI cut-off value of 4 mm, while others proposed different cut-offs. For instance, Faisal et al. identified a 10 mm DOI as the decisive threshold for electing END, Tam et al. proposed 7.25 mm, and Kozak et al. did not specify an alternative cut-off value (33–35). These discrepancies among recent studies highlight the need for a consensus regarding the best DOI threshold to aid clinical decision-making. Conversely, van Lanschot et al. corroborated the established DOI threshold of 4 mm, aligning with previous findings (36). Additionally, Brockhoff et al. proposed specific DOI cut-off values for different subsites, including 2 mm for the tongue, 3 mm for the floor of the mouth, and 4 mm for the buccal mucosa/hard palate (25). In this meta-analysis, tumor thickness and DOI were separated based on the aforementioned definitions, and our findings indicated an increased risk of 118% for LNM in the presence of a DOI > 4 mm and an increased risk of LNM by 172% when the tumor thickness is > 4 mm. Although previous studies revealed DOI as a superior prognostic indicator compared to tumor thickness (23), our study showed a higher risk for LNM with higher tumor thickness. Both DOI > 4 mm and tumor thickness > 4 mm pose a higher risk for LNM, and END should be considered in these patients. However, the level of evidence is low for both, given the significant heterogeneity in reported risk ratios. This information is essential particularly for DOI as it is the primary parameter in the END decision. Thus, we advocate for other parameters with better evidence to predict LNM.

Similarly, despite the questionable level of evidence due to substantial heterogeneity, this meta-analysis revealed that PNI is linked with a 104% higher risk of LNM compared to the baseline. Thus, when diagnosing OCSCC, the presence of PNI on histopathological examination may raise the consideration for END. In previous studies, PNI has emerged as a factor linked to treatment decisions and prognostic outcomes across various cancer types, including OCSCC (37, 38). Still, the consensus regarding its impact remains elusive in the literature, with divergent findings (36, 37). For example, an Australian study did not establish a statistically significant link between PNI and LNM (39). On the other hand, a United States-based study demonstrated a significant association between PNI and LNM (40). This was further augmented by another study from the University of Michigan, encompassing 88 cases of oral cavity squamous cell carcinoma, which revealed that PNI independently served as an adverse factor for nodal metastasis and extra-capsular spread (41). In India, a study from 2014 to 2015 identified a strong correlation between LVI, PNI, and nodal metastasis (42). The significant variation in reported detection of PNI among OCSCC patients, ranging from 5.2% to 90% across studies, can be attributed to the utilization of different criteria. Liebig et al. defined PNI as tumor cells within nerve sheaths or surrounding at least one-third of the nerve circumference, which has been widely adopted (40). However, discrepancies arise when some studies consider PNI present even if tumor cells are merely touching a nerve segment (41). Thus, it is recommended to apply the former criteria rather than the latter as it reduces subjectivity in the assessment process (42). Future research should delve into the qualitative and quantitative aspects of PNI, including parameters like the size of the involved nerve, number of foci, and localization within or around the tumor (43–45). Validating the significance of PNI in guiding treatment decisions is another crucial area for exploration, especially given recent reviews that have not definitively shown improved survival rates with adjuvant postoperative therapy for patients exhibiting PNI (37, 43).

In patients diagnosed with OCSCC, LVI has been recognized as an adverse prognostic factor associated with a poorer prognosis (44). Furthermore, LVI has demonstrated significant associations with tumor grade, invasion pattern, LNM, and local recurrence (45–47). For instance, Martinez Gimeno et al. found that lymph node involvement affected 74.2% of patients with intravascular invasion versus 2.1% of patients without intravascular invasion (48). Additionally, Arora et al. reported that LVI independently predicts cervical LNM, with a sensitivity of 80% and specificity of 74% (4). A meta-analysis by Huang et al., focusing exclusively on early-stage OCSCC, confirmed the predictive value of LVI in predicting LNM (44). In line with the findings of these studies, our meta-analysis resonated with their conclusions, confirming a notable elevation in the risk of LNM by 143% in cases exhibiting LVI. However, Kane et al. did not discover a relationship between LVI and LNM in individuals with early-stage OCSCC (23). Similarly, some studies have questioned the prognostic significance of LVI in OCSCC (49, 50). This discrepancy may arise from inherent heterogeneity in the biological characteristics of OCSCC or challenges in identifying LVI within standard hematoxylin and eosin-stained sections. To mitigate this challenge, there has been advocacy for using immunohistochemistry, like CD31 and D2-40, to further confirm the presence of LVI. However, recent studies have shown limited advantages of immunohistochemical analysis for identifying LVI, particularly in histologically negative cases of tongue carcinomas (51). Another crucial issue is the definition of LVI in OCSCC, which varies across studies. While some define LVI strictly as the presence of tumor cells within the vascular space (52, 53), others extend this definition to encompass tumor cells within or adjacent to the vessels (54). Furthermore, whereas most studies combine lymphatic and vascular invasions into the LVI concept, some studies separated these invasions as venous or lymphatic. This variation in classification methods contributes to divergent findings. Despite these challenges, the results of this meta-analysis underscore the clinical importance of LVI as a predictor for LNM. Given the substantial implications of early detection of positive LVI on prognosis, it becomes imperative to differentiate patients who may benefit from elective neck dissection or additional adjuvant therapies from those lacking LVI. For the latter group, radical local tumor excision coupled with diligent postoperative monitoring may suffice as a suitable treatment strategy.

The primary objective in surgical oncology for OCSCC revolves around achieving negative resection margins. As per the NCCN, a margin ≥5 mm is classified as a clear margin (negative), while a margin of 1-5 mm is considered close, and a margin <1 mm is categorized as involved (positive) (19). A clear margin correlates with a reduced risk of recurrence and prolonged survival periods (55). Nevertheless, a positive margin or insufficient distance from normal tissue (a close margin) carries adverse prognostic implications, warranting adjuvant treatment (56). Hamman et al. reported superior overall survival associated with clear margins after analyzing data from nine studies (57). Meta-analyses conducted by Anderson et al. (58) based on four studies and Bulbul et al. (59) drawing from eight studies demonstrated a heightened likelihood of local recurrence in cases with a positive margin. Our study showed an increased risk of LNM by 45% in the presence of involved or, at least, close margin after excision of the lesion. Our finding provides moderate-to-strong evidence on non-clear margins, as they strongly predict LNM, leading to adverse outcomes. Patients with close and positive margins after excision of OCSCC should be offered END or adjuvant therapy.

Tumor budding is characterized by isolated cells or small tumor clusters comprising fewer than five cells within the stroma present at the invasive front of the tumor (60), representing the most non-cohesive form of invasion. Studies indicate that these budding areas contain cells displaying typical epithelial-mesenchymal transition features, indicating heightened invasiveness (61). Numerous studies consistently link increased tumor budding density with histological indicators of unfavorable outcomes in OCSCC (62–64). Furthermore, previous meta-analyses have reaffirmed the significant impact of tumor budding on clinical outcomes in OCSCC (15, 65, 66). Our meta-analysis proved tumor budding to increase the risk of LNM by 165%. The included studies applied different criteria to determine tumor budding density, yielding significant heterogeneity. Thus, the quality of the evidence is low. We encourage researchers to consolidate the potential importance of tumor budding in estimating the risk of LNM. Nevertheless, two meta-analyses proved the clinical importance of tumor budding on clinical outcomes (65, 66). Thus, tumor budding should be routinely reported in the final histopathological examination report whenever feasible.

Regarding growth patterns, there has been a historical belief that the endophytic morphology signifies a poorer prognosis compared to the exophytic growth pattern. The endophytic pattern was thought to represent the most invasive form of OCSCC, carrying a higher risk of LNM and subsequently lower survival rates (67, 68) Be that as it may, multiple studies have contradicted this notion and denied any association between growth patterns and the outcome of OCSCC (33, 65). Supporting this notion, our comprehensive analysis concluded no significant difference between exophytic and endophytic growth patterns concerning LNM.

H&E staining is a widely available, universal procedure of relatively low cost that holds many of the ideal features for a biomarker. This study focused on detecting which histopathological parameters diagnosed in H&E-stained slides can predict LNM. Some of these parameters can be highlighted in the biopsy before surgery; others can be only interpreted after the whole tumor is resected. For instance, group and single cells as patterns of invasion or non-clear margin showed strong evidence in predicting LNM. These two parameters can be accurately reported only after complete tumor resection. The decision for neck treatment may be delayed until these parameters can be fully interpreted, as they may aid in distinguishing which patient would benefit from additional surgery, especially in scenarios where the traditional parameters are unavailable or show no significant risk for LNM. Beyond the histomorphological features, a growing number of studies assessed parameters at the molecular level as predictors of decreased survival and increased incidence of occult LNM in patients with OCSCC (69, 70). Even though they show promising results in predicting tumor behavior regarding LNM, none of these parameters show consistent evidence. These parameters create an excellent avenue for future research.

To the best of our knowledge, this is the largest and the most comprehensive meta-analysis analyzing 16 different histological parameters across 217 studies for assessing LNM risk among OCSCC patients. Our large sample size makes our evidence more robust and less depicted to false negative results (type II error). However, our study was not free of limitations: First, the inclusion of diverse study designs introduces inherent methodological differences, possibly influencing the robustness and comparability of the findings. Second, the absence of result stratification according to the precise site of OCSCC may obscure the variations in outcomes specific to distinct locations. Third, the variability in classification criteria and definitions for specific histologic parameters across studies introduces a challenge in ensuring consistency and comparability in data analysis. These limitations pave the way for future research by addressing methodological disparities among diverse study designs, stratifying results by precise OCSCC sites, standardizing classification criteria, and establishing uniform cut-off points.

## 4 Conclusion

The risk of LNM in OCSCC is influenced by histologic parameters not commonly analyzed in routine pathologic reports. In this study, the presence of DOI > 4 mm, tumor thickness > 4 mm, PNI, LVI, poorly differentiated tumors, specific invasion patterns (groups and single cells), high tumor budding, and increased tumor size significantly elevated the risk of LNM in OCSCC. Strong consideration should be given to incorporating these parameters in standard pathology reports. In the same way, care should be taken to prevent surgically undertreating patients with potentially positive lymph nodes. Current studies examining histopathological parameters for pre-surgical biopsies, aimed at identifying patients who would benefit from lymph node dissection, are notably scarce, yet immensely necessary. This gap highlights a critical need for future research initiatives in this domain. Specifically, exploring molecular biomarkers assessable through these biopsies could unveil significant predictive values, offering a promising avenue for investigation and potential clinical application.

## Author contributions

SaA: Conceptualization, Data curation, Project administration, Resources, Supervision, Validation, Visualization, Writing – original draft, Writing – review & editing. AbA: Formal Analysis, Investigation, Methodology, Project administration, Resources, Software, Writing – original draft, Writing – review & editing. AB: Data curation, Formal Analysis, Project administration, Resources, Validation, Visualization, Writing – original draft, Writing – review & editing. ZM: Formal Analysis, Writing – review & editing. SuA: Conceptualization, Formal Analysis, Project administration, Software, Supervision, Writing – review & editing. AmA: Data curation, Project administration, Visualization, Writing – review & editing. MN: Investigation, Project administration, Supervision, Writing – review & editing. AR: Investigation, Methodology, Resources, Supervision, Writing – review & editing. MM: Formal Analysis, Investigation, Methodology, Supervision, Writing – review & editing. HM: Conceptualization, Data curation, Funding acquisition, Project administration, Resources, Software, Supervision, Visualization, Writing – review & editing.
