# Supplementary material for: Histopathological predictors of lymph node metastasis in oral cavity squamous cell carcinoma: a systematic review and meta-analysis
Source: Front Oncol. 2024 May 14;14:1401211. doi: 10.3389/fonc.2024.1401211 (PMC11148647; doi:10.3389/fonc.2024.1401211)
Supplement: Supplementary file 1 [file DataSheet_1.docx]

Supplementary Material


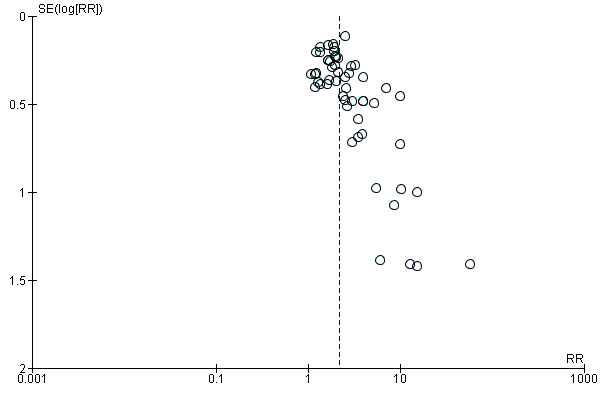


**Supplementary Figure 1.** Publication bias of Risk of LNM according to the depth of invasion.

**
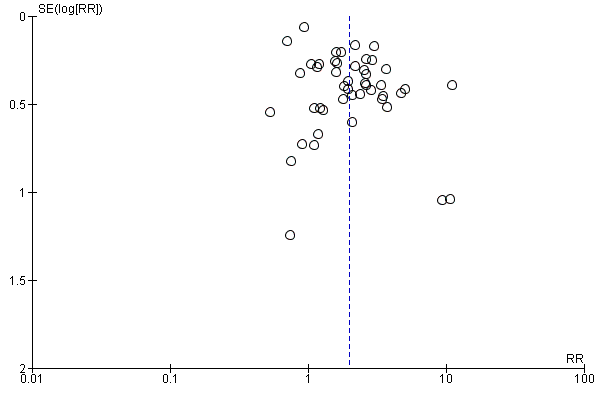
**

**Supplementary Figure 2.** Publication bias of Risk of LNM according to the degree of differentiation.

**
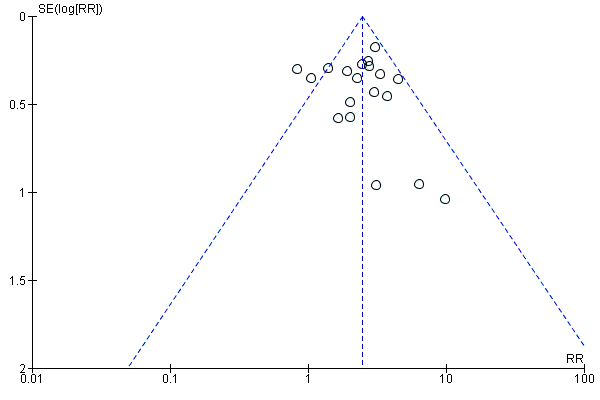
**

**Supplementary Figure 3.** Publication bias of Risk of LNM according to the pattern of invasion.

**
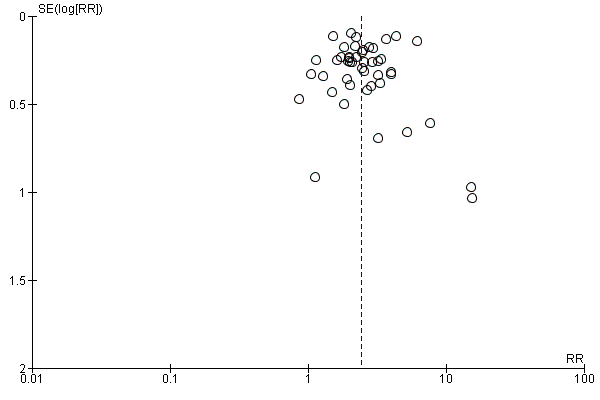
**

**Supplementary Figure 4.** Publication bias of Risk of LNM according to the lymphovascular invasion**.**

**
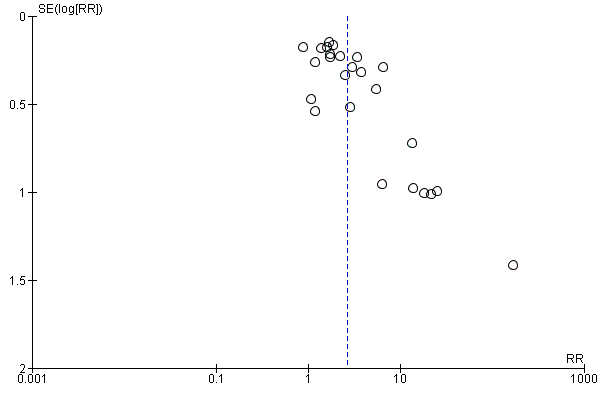
**

**Supplementary Figure 5.** Publication bias of Risk of LNM according to the tumor budding.


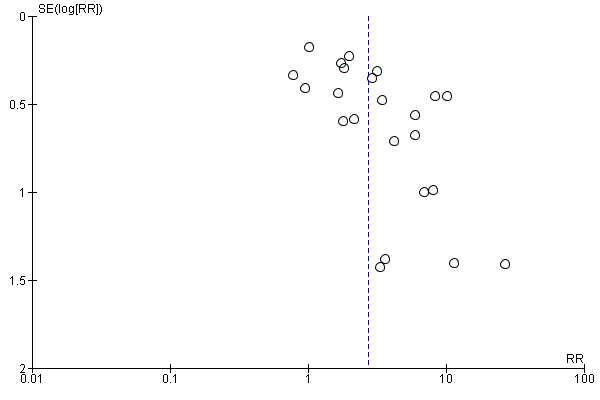


**Supplementary Figure 6.** Publication bias of Risk of LNM according to the tumor Thickness (mm).


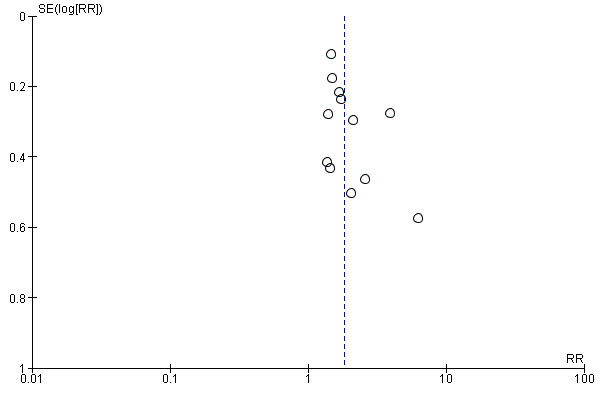


**Supplementary Figure 7.** Publication bias of Risk of LNM according to the tumor grade


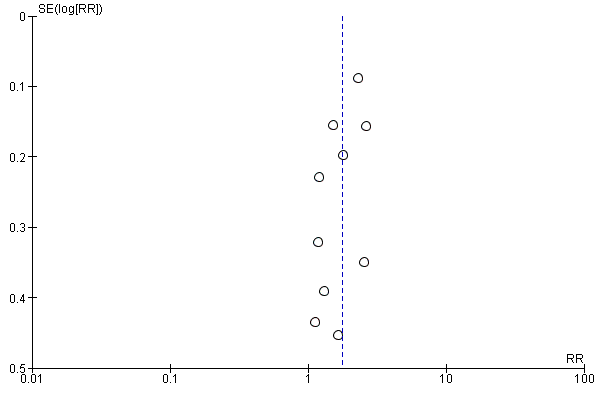


**Supplementary Figure 8.** Publication bias of Risk of LNM according to the tumor size.


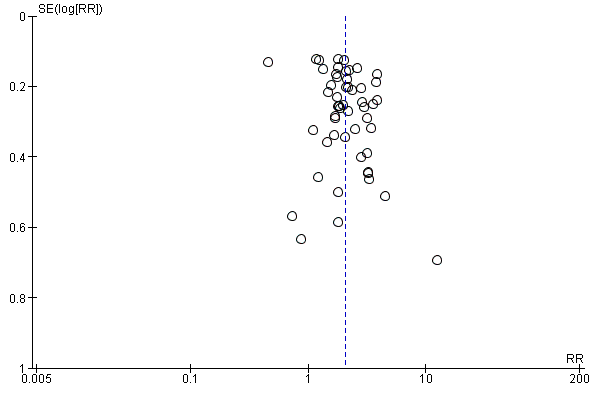


**Supplementary Figure 9.** Publication bias of Risk of LNM according to the perineural invasion.


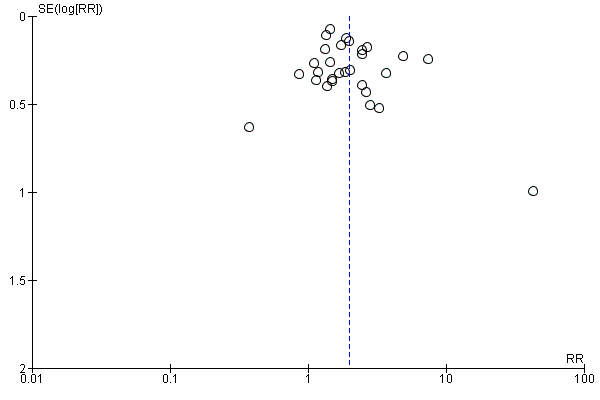


**Supplementary Figure 10.** Publication bias of Risk of LNM according to the tumor stage (T2&3 vs T3&4).


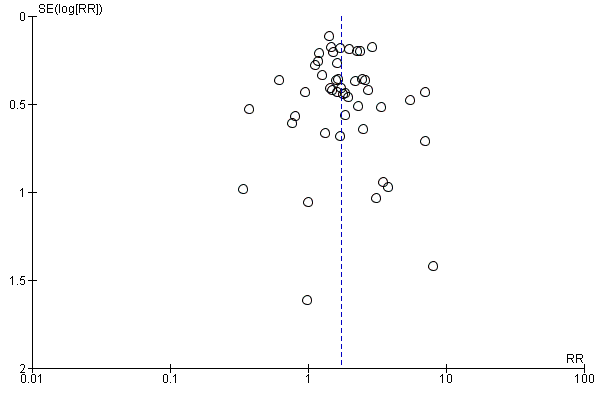


**Supplementary Figure 11.** Publication bias of Risk of LNM according to the tumor stage (T1 vs T2).


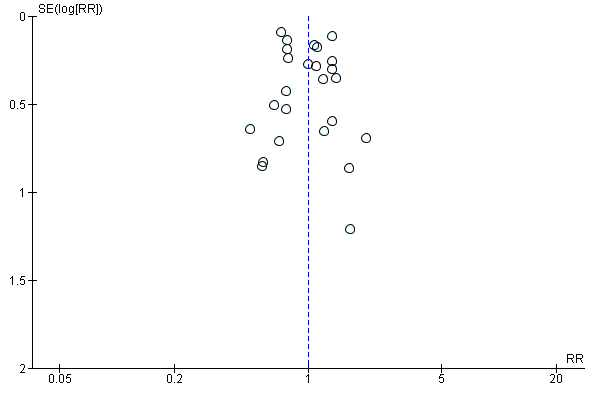


**Supplementary Figure 12.** Publication bias of Risk of LNM according to the tumor stage (T3 vs T4).


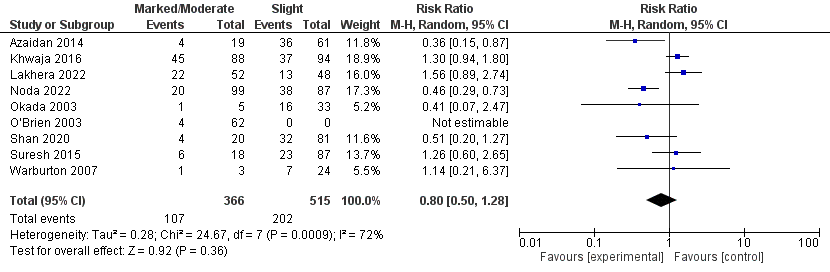


**Supplementary Figure 13.** Risk of LNM according to the lymphoplasmacytic infiltration


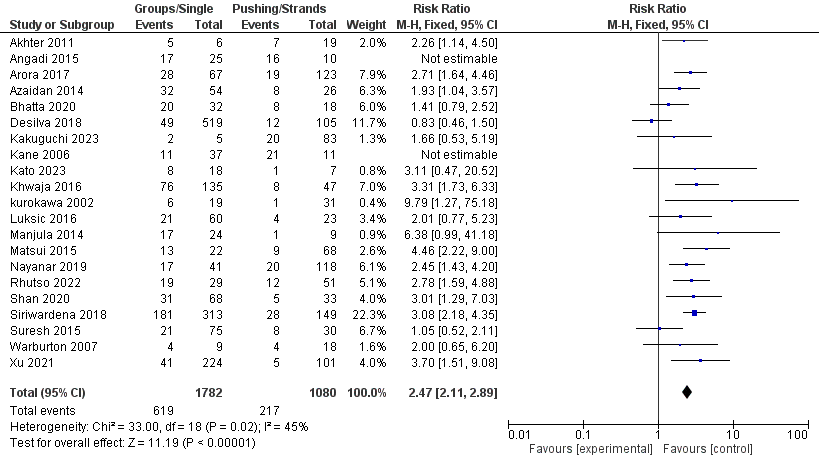


**Supplementary Figure 14.** Risk of LNM according to the pattern of invasion


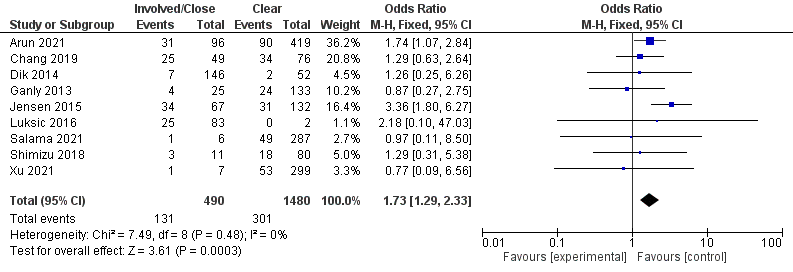


**Supplementary Figure 15.** Risk of LNM according to the margin status.


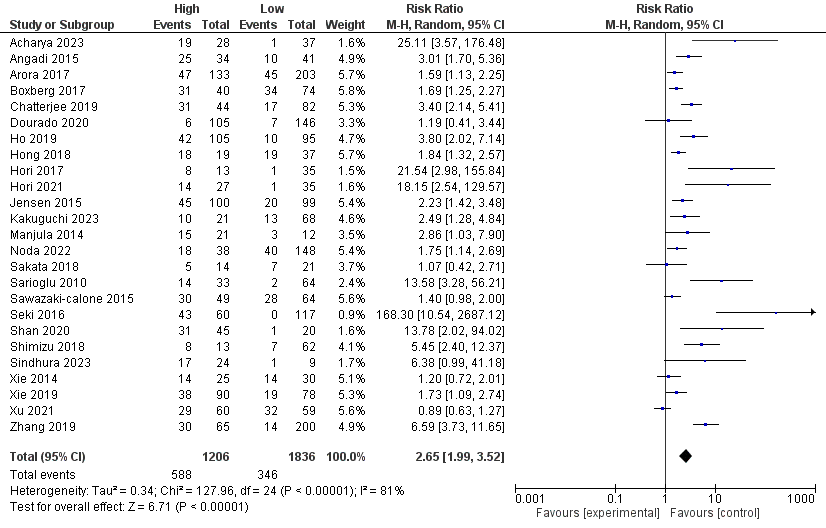


**Supplementary Figure 16.** Risk of LNM according to the tumor budding.


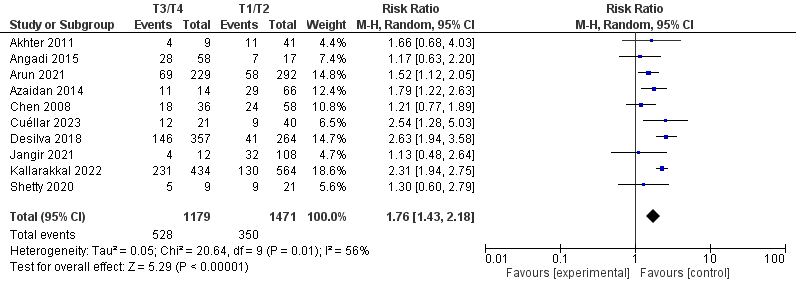


**Supplementary Figure 17.** Risk of LNM according to the tumor size.


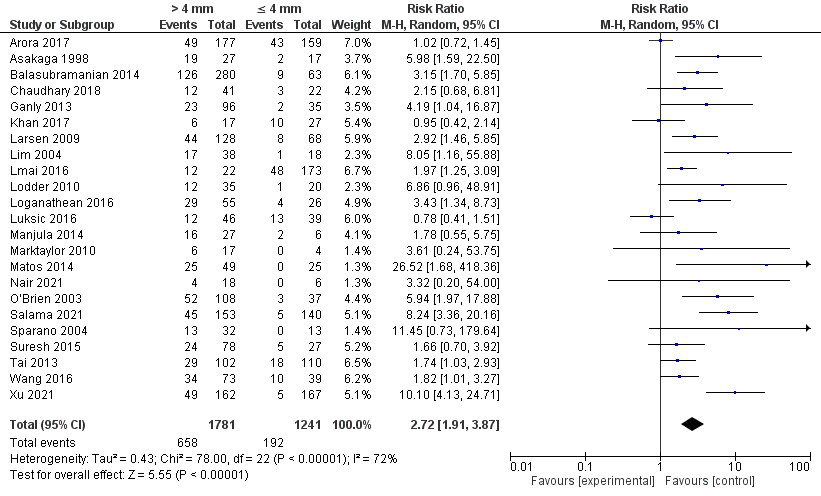


**Supplementary Figure 18.** Risk of LNM according to the tumor thickness (mm).


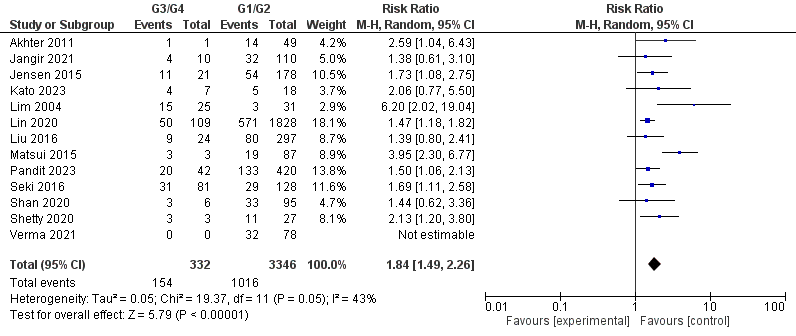


**Supplementary Figure 19.** Risk of LNM according to the tumor grade.


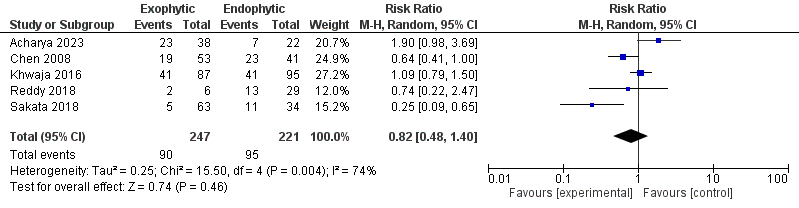


**Supplementary Figure 20.** Risk of LNM according to the growth pattern.


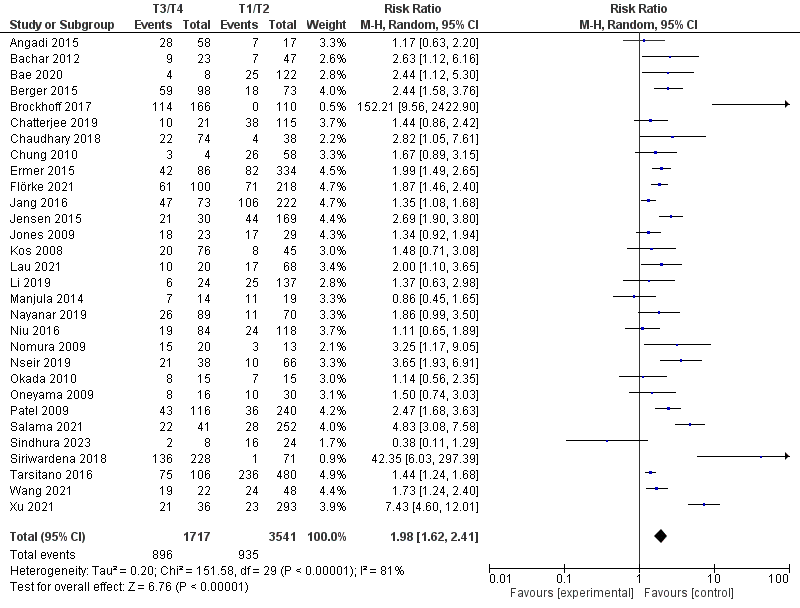


**Supplementary Figure 21.** Risk of LNM according to the tumor stage (T2&3 vs T3&4).

**
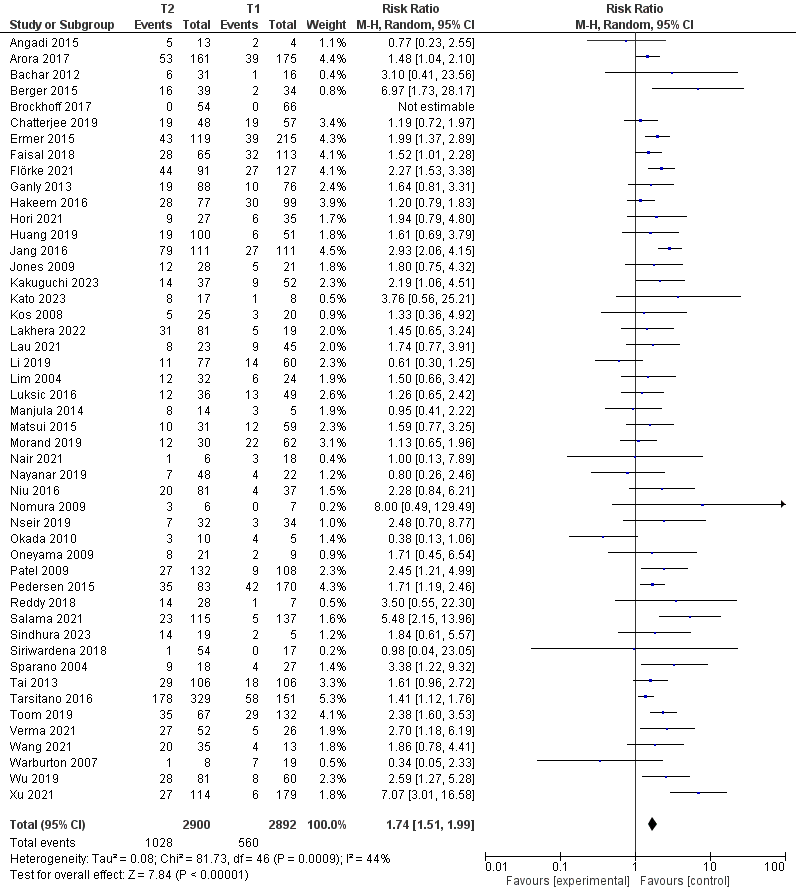
**

**Supplementary Figure 22.** Risk of LNM according to the tumor stage (T1 vs T2).


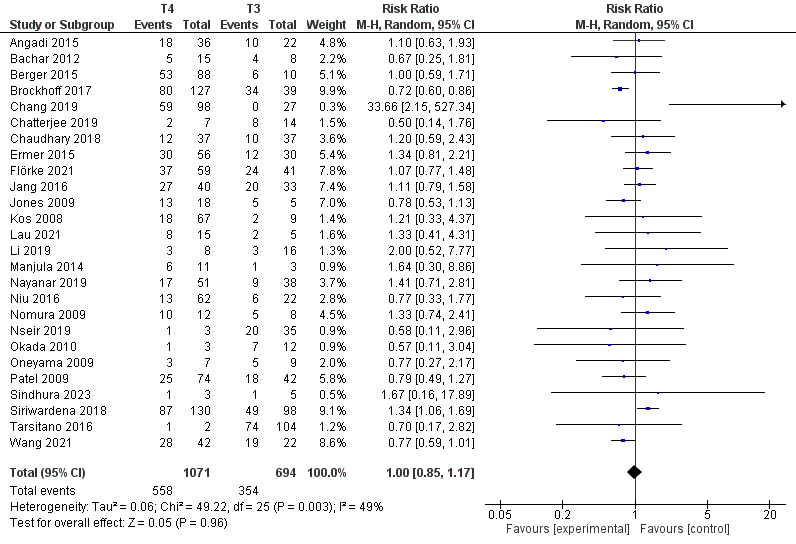


**Supplementary Figure 23.** Risk of LNM according to the tumor stage (T3 vs T4).
