# Supplementary material for: Histopathological predictors of lymph node metastasis in oral cavity squamous cell carcinoma: a systematic review and meta-analysis
Source: Front Oncol. 2024 May 14;14:1401211. doi: 10.3389/fonc.2024.1401211 (PMC11148647; doi:10.3389/fonc.2024.1401211)
Supplement: Supplementary file 2 [file Table_1.docx]

| **Supplementary Table 1: Summary and baseline characteristics of the included studies (1-217)** | | | | | | | | | | | |
| --- | --- | --- | --- | --- | --- | --- | --- | --- | --- | --- | --- |
| **N** | **Study ID** | **No. of patients** | **Study design** | **Site** | **Age,** (mean±SD)y | **Male,** n (%) | **Cancer site,** n (%) | **Cancer stage,** n(%) | **Inclusion criteria** | **Histopathological parameters** | **Conclusion** |
| **1** | **Aaboubout 2021** | **222** | Retrospective Cohort study | Netherlands | 64.5 ± 12.83 | 138(62.2) | 1. Tongue, 128(57.6) 2. Floor of mouth, 65(29.3) 3. Buccal mucosa, 12(5.4) 4. Retromolar trigone, 7(3.2) 5. Gingiva mandible, 7(3.2) 6. Gingiva maxilla, 2(0.9) 7. Lip, 1(0.4) | 1. T1, 117(52.7) 2. T2, 105(47.93) | 1. Surgically treated patients with primary OCSCC  2. Clinically negative lymph nodes (cN0)  3. Identified from January 2006 until December 2012  4. Clinical lymph node status was determined by palpation of the neck and/or by imaging | Depth of invasion | "The DOI is a significant predictor for occult lymph node metastasis in early-stage oral carcinoma. An NPV of 81% was found at a DOI cut-off value of 4 mm. Therefore, an END should be performed if the DOI is >4 mm." |
| **2** | **Acharya 2021** | **65** | Retrospective Cohort study | India | a. <40, 8(12.31%)  b. >40, 57(87.69%) | 54(83.1) | 1. Buccal mucosa 2. Gingivobuccal sulcus 3. Retromolar trigone 4. Lateral border of the tongue 5. Lip  6. Palate and maxillary alveolus | 1. T1, 8(12) 2. T2, 31(48)  3. T3, 18(28)  4. T4, 8(12) | 1. The clinical diagnosis was confirmed based on incisional biopsy report  2. Treated by surgical excision along with neck dissection  3. OSCC cases only in which both pre and corresponding postoperative sections were available along with relevant details | Tumor budding | "Findings are in accord that the BD model is an objective, simplest, fast, and most effective tool to evaluate the prognosis in OSCC. However, its value in preoperative biopsies is questionable. There was a significant association between preoperative and postoperative biopsies for assessing B, but the discrepancy was with regard to D measurement. Further investigations are required to substantiate the value of the BD model in preoperative biopsies of OSCC. Observations of this analysis recommend that a reasonable representative biopsy may increase consistency in the assessment of preoperative B and precision in BD model evaluation in OSCC." |
| **3** | **Acharya 2023** | **60** | Retrospective Cohort study | India | 49 ± 10.75 | 48(80) | 1. Buccal mucosa/retromolar trigone,40(33.33) 2. Alveolus/gingiva, 17  3. Tongue, 3(28.33) | 1. T1, 26(43.33) 2. T2, 33(55) 3. T3, 1(1.67) | 1. Cases of squamous cell carcinoma  2. Reported in our institution from 2007 to 2010 3. Graded according to Modified Broders’ descriptive system and Anneroth et al., MFG system | Growth pattern | "Moderate to good agreement between observers greatly increases the validity of the MFG system. The multifactorial malignancy grading could serve as a predictor for metastasis in the cervical lymph nodes." |
| **4** | **Adel 2015** | **571** | Retrospective Cohort study | Taiwan | 52.7 ± 11.7 | 516(90.4) | OCSCC, 571(100) | 1. T1, 97(16.99)  2. T2, 127(22.24)  3. T3, 92(16.11)  4. T4, 255(44.66) | 1. Patients who were diagnosed with OSCC  2. Underwent surgery as the primary modality of therapy  3. All patients were followed up for at least 18 months or until death | Lymphovascular invasion | "Moderate to good agreement between observers greatly increases the validity of the MFG system. The multifactorial malignancy grading could serve as a predictor for metastasis in the cervical lymph nodes." |
| **5** | **Adel 2016** | **277** | Retrospective Cohort study | China | a. <50, 116(41.88%)  b. >50, 161(58.12%) | 253(91.34) | 1. OTSCC, 114(41.16)  2. Buccal mucosa, 106(38.27)  3. Others,57(20.58) | 1. T1, 62(22.4)  2. T2, 46(16.6)  3. T3. 37(13.4)  4. T4, 132(47.65) | All Patients received radical surgery with curative intent as the primary modality of therapy. | Lymph Node Density | "LND was correlated with the incidence of distant metastasis and DFS and OS in OSCC. Concurrent high preoperative SCC-Ag and CRP levels exhibited a linear correlation with LND and can be used as predictors for adverse tumor features. Preoperative elevated serum levels can be used to stratify patients for adjuvant treatment and must be considered complementary to the prognostic significance of LND." |
| **6** | **Aires 2017** | **274** | Retrospective Cohort study | Brazil | 59.9 ± 10.9 | 210(76.6) | 1. OTSCC, 97(35.4) 2. Floor of mouth, 91(33.21)  3. Buccal mucosa, 16(5.84)  4. Hard palate, 9(3.28)  5. Lower gingiva, 14(5.11)  6. Upper gingiva, 8(2.92) 7. Retromolar trigone, 39(14.23) | 1. T1, 57(20.8)  2. T2, 62(22.6)  3. T3, 42(15.3)  4. T4, 113(41.2) | 1. All patients with OCSCC (excluding the lip)  2. Confirmed by histopathological analysis  3. Underwent surgical resection with curative intent | 1. Perineural invasion 2. Tumor thickness | "Patients with OCSCC who have contralateral lymph node metastasis, tumors with a thickness >25 mm, angiolymphatic invasion, or locoregional recurrence after surgical treatment have a greater risk of developing distant metastasis." |
| **7** | **Aivazian 2014** | **318** | Retrospective Cohort study | Australia | 54 ± 8.21 | 194(61) | 1. OTSCC, 130(40.88) 2. Floor of mouth, 91(28.62)  3. Alveolus, 35(11)  4. Retromolartrigone, 23(7.23)  5. Buccal, 32(10.06)  6. Hard palate, 4(1.26)  7. Unspecified, 3(0.94) | 1. T1, 108(33.96)  2. T2, 106(33.33) 3. T3, 26(8.17)  4. T4, 76(23.9) | 1. Patients with OSCC  2. Undergoing primary surgical treatment with curative intent  3. Between 1995 and 2010 | Perineural invasion | "Multifocal PNI is associated with poor outcomes even with PORT suggesting consideration of therapeutic escalation, particularly with involved nerves 1 mm. Unifocal PNI did not affect prognosis even in the absence of PORT, which may not be required if this is the sole risk factor. Prospective validation and testing of these hypotheses is essential before implementation." |
| **8** | **Akhter 2011** | **50** | Prospective Cohort Study | Bangladesh | NR | NR | OSCC,50(100) | 1. T1, 8(16) 2. T2, 33(66) 3. T3, 7(14)  4. T4, 2(4) | 1. All cases of OSCCs  2. Registered from 1 Nov 2003 to 31 Dec 2004  3. The age group of the study population was above 30 years  4. Patients of both genders were randomly selected | 1. Tumor grade  2. Tumor size  3. Pattern of invasion  4. Degree of differentiation | "Anneroth’s classification can be taken as a standard diagnostic factor and predictive factor of lymph node metastasis." |
| **9** | **Amit 2013** | **2258** | Retrospective Cohort study | Multicenter | 55 ± 12 | 1516(67) | OSCC, 2258(100) | NR | 1. Patients were preoperatively staged by the anatomical extent of the disease  2. Staging was done according to the current tumor, node, metastasis (TNM) classification system  3. Treated for OSCC with primary surgery with or without adjuvant radiotherapy or chemoradiotherapy  4. All patients underwent unilateral or bilateral neck dissection involving levels I–III, I–IV, or I–V | 1. Depth of invasion  2. Tumor stage  3. Margin status | "In conclusion, in this study, we investigated a selected group of patients with a pN-neck classification. We demonstrated that clinical evidence of neck metastases is associated with a high risk of regional recurrence, regardless of the pathological N classification. According to our results, clinical evidence of nodal metastases is an independent prognostic factor of OS and DSS. Further studies are warranted in order to decide whether these patients would benefit from adjuvant treatment." |
| **10** | **Angadi 2015** | **75** | Retrospective Cohort study | India | 45.1 ± 11.5 | 64(85.3) | Mostly Buccal | 1. T1, 4(5.33) 2. T2, 13(17.33)  3. T3, 22(29.33)  4. T4, 36(48) | 1. OSCC cases  2. Had undergone surgical resection of the primary tumor and radical neck dissection for OSCC  3. From January 2010 to December 2013  4. None of the patients had received any form of adjuvant therapy prior to surgery | 1. Tumor size  2. Tumor budding  3. Lymphovascular invasion  4. Pattern of invasion  5. Degree of differentiation  6. Perineural invasion | "Tumor budding is frequently encountered histologic marker in OSCC. High-intensity tumor budding is a strong independent prognostic factor for prediction of lymph node metastasis." |
| **11** | **Arora 2017** | **336** | Retrospective Cohort study | India | 55.4 ± 14.3 | 212(63) | 1. Buccal mucosa, 117(35) 2. OTSCC, 107(32) 3. Retromolar trigonum, 41(12) 4. Floor of mouth, 33(10) 5. Mandibular gingival, 22(6.5) 6. Lip, 9(2.5) 7. Buccal-maxillary gingival, 07(2) | T1 and T2 | 1. Patients with clinical stage I/II (cT1/T2/N0/M0)  2. Availability of well-stained, good-quality sections with tumor interface  3. Follow-up was available for at least five years or until death event | 1. Tumor size  2. Tumor budding  3. Lymphovascular invasion  4. Pattern of invasion  5. Degree of differentiation  6. Perineural invasion | "The risk of lymph node metastasis in OSCC is influenced by many histologic parameters that are not commonly analyzed in routine pathologic reports. In this study, we graded these significant independent factors (DOI, mode of invasion, grade of tumor, LVI, PNI, lymphoid response at tumor-host interface, and tumor budding) to design a scoring system that permits accurate evaluation of the risk of metastasis with accuracy independent of the traditional TNM system or isolated histologic parameters. The need for neck node dissection can be predicted depending upon the scores obtained." |
| **12** | **Arun 2021** | **339** | Retrospective Cohort study | India | 55.3 ± 11.9 | 278(70.6) | 1. ORSCC, 176(44.7)  2. Gingivobuccal, 218(55.3) | NR | 1. Patients who underwent surgery as the primary method of treatment for OSCC  2. From April 2011 through December 2017 | 1. Tumor size  2. Margin status  3. Lymphovascular invasion  4. Degree of differentiation  5. Perineural invasion | "Significant differences are noted in the prevalence of pathologic risk factors between the single node positive and node negative groups. Within the single-node positive group, tumor factors like the gingivobuccal subsite, PNI, and margin positivity impacted survival. Among nodal factors, a deposit size of 12 mm or more and the presence of ENE are pointers to poor prognosis. These patients would benefit from adjuvant treatment." |
| **13** | **Asakage 1998** | **44** | Retrospective Cohort study | Japan | 61.75 ± 14.14 | 27(61.36) | OTSCC,44(100) | T1 and T2 | 1. Patients with previously untreated Stage I/II carcinoma of the tongue  2. All patients were treated with partial glossectomy only | 1. Tumor thickness  2. Degree of differentiation  3. Perineural invasion | "Current study data indicate that patients with Stage I/II carcinoma of the tongue ú 4 mm in thickness are at increased risk for subsequent cervical metastasis. Thus, conservative suprahyoid neck dissection is indicated in patients with Stage I/II carcinoma of the tongue ú 4 mm in thickness." |
| **14** | **Azaidan 2014** | **80** | Retrospective Cohort study | Iraq | a. <=29, 6(7.5%)  b. 40-80, 74(92.5%) | 54(67.5) | 1. Floor of the mouth, 4(5)  2. Anterior 2/3 of the tongue, 27(33.75)  3. Buccal mucosa, 14(17.5)  4. Alveolar ridge, 15(18.75)  5. Hard palate, 7(8.75)  6. Lips, 13(16.25) | 1. T1, 24(30) 2. T2, 42(52.5)  3. T3, 9(11.25) \| 4. T4, 5(6.25) | 1. Patients with OSCC  2. Underwent surgical excision of primary oral cancer and neck dissection | 1. Depth of invasion  2. Tumor size  3. Pattern of invasion  4. Degree of differentiation  5. Lymphoplasmacytic infiltration | "Tumor depth is a reliable factor to predict nodal metastasis, and tumor depth of 4 mm can be considered as a suggested standard Iraqi cutoff number in staging and management of early oral squamous cell carcinoma." |
| **15** | **Bachar 2012** | **70** | Retrospective Cohort study | Canada | 57.62 ± 7.5 | 33(47) | Buccal mucosa, 70(100) | 1. T1, 16(22.86) 2. T2, 31(44.28)  3. T3, 8(11.43)  4. T4, 15(21.43) | Patients with buccal mucosa carcinoma | Tumor stage | "Most patients present with early-stage local disease (T1-T2). Surgery was the treatment of choice in 87% of patients managed at PMH, with 36% receiving postoperative radiotherapy. The 5- year local, regional, and overall control rates were 57.5%, 83.5%, and 50%, respectively. The 5-year overall and recurrence-free survival was 69% and 46%, respectively. We found that the only significant predictors of survival were nodal status and extranodal extension. Further, multi-institutional studies should be performed to better characterize the unique features of this relatively uncommon disease in the Western world and to better evaluate the primary treatment modality." |
| **16** | **Bae 2020** | **130** | Retrospective Cohort study | Germany | 52 ± 18.48 | 84(64.12) | OSCC, 130(100) | All | 1. cN0 OCC patients  2. Underwent 18F-FDG PET/CT scanning and   subsequent curative surgery and neck dissection | 1. Depth of invasion  2. Degree of differentiation | "Tumour 18F-FDG PET/CT parameters might predict occult metastasis and survival in cN0 OCC patients." |
| **17** | **Balasubramanian 2014** | **343** | Retrospective Cohort study | Australia | 59.75 ± 21.65 | 227(66.2) | 1. OTSCC, 222(64.72)  2. Floor of mouth, 121(35.28) | 1. T1, 137(39.94)  2. T2, 139(40.52)  3. T3, 32(9.33)  4. T4, 35(10.2) | 1. Patients with OSCC 2. The neck was considered positive in the presence of pathologically   proven nodal metastases on neck dissection or during follow-up | Tumor thickness | "Thin FOM tumors (2.1–4 mm) have a high rate of nodal metastases. Elective neck dissection is appropriate in FOM tumors P2 mm thick and in tongue tumors P4 mm thick." |
| **18** | **Balla 2020** | **40** | Retrospective Cohort study | India | NR | 22(55) | 1. Palate,14(35)  2. Tongue, 12(30)  3. Buccal mucosa, 10(25)  4. Floor of the mouth, 3(7.5)  5. Lip, 1(2.5) | All | 1. Cases of diagnosed primary OSCC  2. With radical or elective neck dissection | Degree of differentiation | "Statistically, a significant association was observed between TNM staging and Immunomorphological patterns of Lymph Node (P < 0.01). The relationship between the histopathological grade of the primary tumor of Squamous Cell Carcinoma and the Immunomorphological patterns was statistically significant (P < 0.01). Statistically, a significant association was observed between Sinus Histiocytosis pattern and decreased occurrence of nodal metastasis (P < 0.01)." |
| **19** | **Beggan 2016** | **58** | Retrospective Cohort study | Ireland | a. ≥70, 15(25.86%)  b. <70 years 43(74.14%) | 44(75.8) | Floor of the mouth, 58(100) | 1. T1, 22(37.93)  2. T2, 19(32.76)  3. T3, 3(5.17)  4. T4, 14(24.14) | 1. Cases of FOM SCC  2. Independently evaluated for POI and LVI by three pathologists  3. Interobserver variability was analyzed using Fleiss Kappa statistics | 1. Pattern of invasion  2. Lymphovascular invasion | "Interobserver agreement in the diagnosis of LVI was substantial. POI at the 50% and 20% cut-offs is moderately reproducible and has prognostic value in FOM SCC. Further studies are necessary to establish the optimum quantitative cut-off for POI. " |
| **20** | **Bera 2022** | **320** | Retrospective Cohort study | India | 57.9 ± 5.98 | NR | 1. Buccal mucosa, 120(37.5) 2. Tongue, 65(20.3) 3. Floor of mouth, 68(21.3) 4. Mandibule alveolus, 36(11.3) 5. Maxilla alveolus, 12(3.8)  6. RMT, 19(5.9) | NR | 1. Patient records with biopsy-proven OSCC  2. Underwent surgery as the primary modality of treatment  3. Patients who presented with Clinically N0 neck | Degree of differentiation | "The conclusion that can be drawn from both these studies is that the cutoff value of 4 mm for DOI is still optimum." |
| **21** | **Berger 2015** | **171** | Retrospective Cohort study | Germany | 62.05 ± 18.76 | 102(59.6) | Maxillary SCC, 171(100) | 1. T1, 34(19.88)  2. T2, 39(22.81)  3. T3, 10(5.84)  4. T4, 88((49.71) | 1. Patients underwent a tumor resection 2. Indication for an ipsilateral neck dissection was given when the staging score was cT  3. Patients with cT1 and cN- Tumours 4. Advanced tumor stage (cT4) and patients with cT | 1. Tumor stage  2. Tumor Grade | "Reviewing recent literature underlined by the illustrated data, we put up for discussion the treatment of SCC of the maxilla as similar to therapy protocols for SCC of the oral cavity. This would include an ipsilateral ND even in the low tumor stage and in T4 staged tumors on both sides. However, prospective multicentre studies are needed to verify and recommend these therapy assumptions." |
| **22** | **Bhatlawande 2019** | **50** | Retrospective Cohort study | India | 49.88 ± 12.77 | 40(80) | NR | NR | 1. Patients with OSCC 2. The grades of primary tumors were classified into WD, MD, and PDSCC 2. Histopathologic features of the tumor were included as follows, per Broder’s   and Byrne’s classification: tumor grade, invasive front, neural invasion, and vascular invasion  3. Evaluation of LNs and comparison with clinical and histopathological parameters was by histopathology | Degree of differentiation | "The present study adds to the understanding of lymph node immunoreactivity patterns and their correlation with tumor grade. We recommend further study of lymph node patterns for all sentinel lymph node biopsies and routine neck dissections for OSCCs." |
| **23** | **Bhatta 2020** | **50** | Retrospective Cohort study | India | 34–76) | 46(92) | 1. Gingivobuccal sulcus, 32(64)  2. RMT, 8(16)  3. Alveolar mucosa, 6(12)  4. Tongue, 4(8) | 1. T1, 7(14)  2. T2, 11(22)  3. T3, 22(42)  4. T4, 10(20) | 1. OSCC cases  2. Hematoxylin and eosin-stained slides representing primary tumors and   selective neck dissections of OSCC patients  3. Operated from 2016 to 2020 were retrieved from department archives | Pattern of invasion | "This study supports the presence of a strong immunological host-tumor relationship." |
| **24** | **Bjerkli 2020** | **150** | Retrospective Cohort study | Norway | NR | 92(61.3) | OTSCC, 150(100) | 1. T1, 91(60.67)  2. T2, 28(18.67)  3. T3, 8(5.33) 4. T4, 2(1.33) | Patients with OSCC | 1. Tumor budding  2. Depth of invasion | "Reclassification according to TNM8 shifted many tumors to a higher T-status and also increased the prognostic value of the T-status. This supports the implementation of depth of invasion to the T-categorization in TNM8. Tumor budding correlated with lymph node metastases and survival. Therefore, information on tumor budding can aid clinicians in treatment planning and should be included in pathology reports of oral tongue squamous cell carcinomas." |
| **25** | **Boxberg 2017** | **157** | Retrospective Cohort study | Germany | 60.1 ± 8.18 | 103(65.7) | OSCC, 157(100) | Advanced stage | 1. Patients with primary, untreated local, and locoregional OSCC 2. All tumors had their punctum maximum in the oral cavity | Tumor budding | "We recapitulated and validated almost exactly the strong prognostic impact of a grading algorithm proposed recently for squamous cell carcinoma of the lung in OSCC. Our data may pave the way for a prognostically highly relevant future squamous cell carcinoma grading." |
| **26** | **Brockhoff 2017** | **286** | Retrospective Cohort study | USA | 63 ± 12.5 | NR | 1. Tongue  2. Floor of mouth  3. Retromolar trigone  4. Alveolus/hard palate | 1. T1, 66(23.08)  2. T2 , 54(18.89)  3. T3 , 39(13.64)  4. T4 , 127(44.4) | All patients who had received any form of neck dissection with resection of a primary oral cavity squamous cell carcinoma | Depth of invasion | "Depth of invasion and the location of the tumor are two important variables to consider when making treatment recommendations to patients with clinical N0 disease." |
| **27** | **Brown 2002** | **100** | Prospective Cohort Study | England | 61 ± 13 | 56(56) | 1. Buccal, 5(5) 2. Tongue, 12(12) 3. Retromolar, 21(21) 4. Floor of mouth, 42(42) 5. Alveolus, 20(20) | NR | 1. Patients underwent a resection of the mandible (either rim or segment)  2. Patients with squamous cell carcinoma | Pattern of invasion | "Larger or more deeply invading tumors in the soft tissue are more likely to invade the mandible and show the more aggressive (invasive) form of tumor spread, reducing the options of a more conservative (rim) resection. Tumors tend to enter the mandible at the point of abutment, which in both the dentate and edentulous jaw is often at the junction of the reflected and attached mucosa. A point of tumor entry below the occlusal ridge or gingival crest should be assumed when planning rim or marginal resections of the mandible." |
| **28** | **Caponio 2021** | **200** | Retrospective Cohort study | Italy | a. ≥65, 104(52%)  b. <65, 96(48%) | 126(63) | OSCC, 200(100) | 1. T1, 38(19)  2. T2, 59(29.5)  3. T3, 56(28)  4. T4, 47(23.5) | 1. Surgical samples of primary OTSCC  2. Age above 18 years  3. Absence of preoperative chemo or radiation therapy 4. No human papilloma virus  5. Follow-up data of at least three years for living patients | 1. Pattern of invasion  2. Perineural invasion | "PNI emerged to be a fingerprint of aggressive behavior in OTSCCs. In particular, in patients with early T-status tumors, the evidence of PNI may represent a factor leading to a worse DSS. Moreover, patients with tumors characterized by intratumoral PNI, together with high-class grading and WPOI systems, were more likely to exhibit positive lymph node metastasis. PNI might serve as an additional prognostic factor in OTSCC, and by integrating PNI in the current staging system, further improvements in prognostication might be reached." |
| **29** | **Chandavarkar 2015** | **30** | Retrospective Cohort study | India | 35-70 |  | OSCC, 30(100) | NR | 1. Oral squamous cell carcinoma (OSCC) was taken  2. Ten of these were well-differentiated SCCs (WDSCC), ten moderately differentiated SCCs (MDSCC), and ten poorly differentiated SCCs (PDSCC) | 1. Pattern of invasion  2. Tumor grade | "Immuno-morphological assessment of draining lymph nodes reflects the immune status of the patient with respect to metastases. This may facilitate identification of high and low-risk patients and help in planning appropriate therapy for the high-risk patients." |
| **30** | **Chandler 2011** | **61** | Retrospective Cohort study | USA | NR | NR | OSCC, 61(100) | T1, 61(100) | 1. Cases were identified with OSCC 2. Had clinically negative lymph nodes, negative margins of resection  (tumor C0.1 cm from margin of resection)  3. Possessed at least two years of clinical follow-up | Depth of invasion | "Cases with a DOI greater than 3 mm had a 29.7% PPV of occult lymph node metastasis. Cases with muscle invasion had a 43.7% PPV of local tumor recurrence. Cases with maximum DOI of greater than 3 mm had a 40.4% PPV of tumor recurrence." |
| **31** | **Chang 2019** | **341** | Cross-sectional study | Taiwan | 52.8 ± 17.61 | 313(90.98) | 1. Oral cavity 2. Lips | All | Potential prognostic implications of oral squamous cell carcinoma with adverse features | 1. Depth of invasion  2. Margin status | "When determining survival prognosis for patients with a pN0 status, we recommended including all adverse features. In contrast, extranodal extension was the most important prognostic factor for patients with a pN+ status." |
| **32** | **Chang 2010** | 92 | Retrospective Cohort study | Taiwan | Mean(52.34) | 129(84.3) | 1. Tongue  2. Buccal mucosa  3. Gingiva | All | Patients with OSCC were retrieved from the archives of the Department of Pathology. | Pattern of invasion | "Our study confirms the validity of the IPGS, an indicator that is simple and easy to use. IPGS not only provides a histological assessment of biological behavior but also offers an independent prognostic factor that may influence the treatment of OSCC." |
| **33** | **Chang 2017** | 389 | Retrospective Cohort study | China | 52.65 ± 17.61 | 355(91.3) | OSCC, 389(100) | All | 1. Patients with primary OSCC  2. Underwent surgical resection in 2002–2015 | Lymph Node Density | "Lymph node density, at a cutoff of 0.05, was an independent predictor of OS and DFS. OS and DFS underwent multiple analyses, and LND remained significant. The pathologic N stage had no influence in the OS analysis. Clinical relevance LND is a more reliable predictor of survival in betel nut-chewing patients for further post-operation adjuvant treatment, such as reoperation or adjuvant radiotherapy." |
| **34** | **Chatterjee 2019** | 126 | Retrospective Cohort study | India | 22.2 ± 8.3 | 104(82.5) | Buccal mucosa and oral tongue, 126(100) | Early | 1. All the cases of buccal mucosa and oral tongue SCC  2. Underwent resection with cervical LN dissection  3. From July 2012 to December 2017 | 1. Depth of invasion  2. Tumor budding  3. Lymphovascular invasion  4. Degree of differentiation  5. Perineural invasion | "WPOI and tumor budding are important risk factors for predicting LN metastasis in all stages of OSCC and associated with a poorer outcome in early-stage tumors. These are easy and reliable prognostic factors and should be included in the histopathological reporting guidelines." |
| **35** | **Chaudhary 2018** | 112 | Retrospective Cohort study | India | 46.1 ± 15.87 | 98(87.5) | OSCC, 112(100) | T1, T2, T3  and T4 | 1. Patients having oral tongue squamous cell cancer  2. With clinically and radiologically (computerized tomography [CT]/magnetic resonance imaging) N0 neck and T stage T2, T3, T4  3. Underwent surgery as the primary mode of treatment | 1. Tumor thickness  2. Degree of differentiation | "As is evident, there is no consistent statistically significant factor that can be attributed as a predictor of occult metastasis in head and neck cancer of the oral cavity. The search to identify reliable and accurate predictor(s) of occult metastases or approaches to the management of patients with cN0 oral SCC must continue. In the absence of such predictors, keeping the high incidence of occult metastasis in mind, we recommend END in all cases of N0 OSCC." |
| **36** | **Chen 2008** | 94 | Retrospective Cohort study | Taiwan | 52 ± 16.17 | 80(85.1) | OSCC, 94(100) | All | 1. All patients with OSCC  2. Had documented pathological reports of the surgical specimens   of both the oral or primary site and the neck lymph nodes | 1. Depth of invasion  2. Growth pattern  3. Tumor size  4. Lymphovascular invasion  5. Degree of differentiation  6. Perineural invasion | "Cases of tongue cancers may be subject to a higher incidence of neck nodal metastasis if they are moderate or poorly differentiated, have an invasion depth >3 mm and positive perineural invasion or lymphovascular permeation at the time of presentation. The authors suggest that an elective neck dissection or other neck treatment, such as radiotherapy, should be considered in tongue cancers with these histopathological risk factors." |
| **37** | **Chuang 2020** | 102 | Retrospective Cohort study | Taiwan | 57.25 ± 15.59 | 52(50.9) | OTSCC, 102(100) | T1, 102(100) | 1. Primary early OTSCC  2. From January 2010 to September 2015  3. The tumor was restaged according to the AJCC 8th edition Cancer Staging  4. Diagnostic workup for all patients consisted of at least one imaging modality CT, MRI, or PET-CT scan | 1. Depth of invasion  2. Perineural invasion | "Histologic grade ≥2 was an adverse prognostic factor of neck recurrence and was significantly associated with poor cancer-specific survival in T1-2N0 early oral tongue cancer patients. Therefore, prophylactic neck dissection or prophylactic adjuvant radiation therapy to the neck may be considered in T1-2N0 early oral tongue cancer with histologic grade ≥2 of the primary tumor." |
| **38** | **Chung 2010** | 62 | Retrospective Cohort study | South Korea | 51.5 ± 14.42 | 37(59.6) | OTSCC, 62(100) | 1. T1, 29(46.7)  2. T2, 29(46.7) 3. Others, 4(6.45) | 1. Patients with primary OSCC  2. Underwent surgical resection | 1. Depth of invasion  2. Degree of differentiation  3. Perineural invasion | "ILD showed a strong correlation with regional metastasis in patients with squamous cell carcinoma of the tongue." |
| **39** | **Cracchiolo 2018** | 381 | Retrospective Cohort study | USA | 57 ± 22.51 | 222(58.3) | OTSCC, 381(100) | All | 1. Patients diagnosed with oral tongue SCC  2. T1 to T4 classifications | 1. Lymphovascular invasion  2. Perineural invasion | "The presence of PNI in oral tongue SCC predicts worse DSS, with distant recurrence as the most common pattern of failure. High PNI foci density is associated with worse DRFS." |
| **40** | **Cuéllar 2023** | 61 | Retrospective Cohort study | Spain | 59.72 ± 15.72 | 40(65.6) | OTSCC, 61(100) | T1,T2 and T3 | 1. Patients had initial clinical stages of oral tongue SCC  2. (cT1, cT2, cN0, stages I, II) according to the AJCC 3. Patients had undergone surgery  4. Surgical procedures consisted of extensive tumor removal and elective neck dissection 5. Patients were over the age of 18 | Tumor size | "Depth of invasion is a histological risk factor in early clinical stages of oral tongue squamous cell carcinoma. Depth of invasion impacts negatively on patient prognosis, is capable of modifying the T category and the global tumor staging, and is associated with the presence of cervical metastatic disease, perineural invasion, and tumoural differentiation grade." |
| **41** | **D'Cruz 2021** | 570 | RCT | India | 47.6 ± 15.87 | 430(75.4) | Tongue, 484(84.91) | T1 and T2 | 1. Clinically node-negative histologically proven early oral cancers (T1/T2)  2. With lateralized lesions amenable to peroral excision  3. Designed to address the appropriate management of the neck | Depth of invasion | "The results of our study provide proof of concept supporting the hypothesis that for early oral cancers with DOI 10 mm, adequate neck treatment can negate the detrimental impact of DOI on survival. Tumors with DOI >10 mm are associated with an increase in other adverse factors, raising the possibility that DOI may not be an independent prognostic factor in this group of patients as well." |
| **42** | **Desilva 2018** | 623 | Retrospective Cohort study | Newzeland | a. <40, 28(4.51%)  b. >40, 593(95.49%) |  | Buccal mucosa and Tongue | All | 1. All patients who underwent surgery for OSCC of the tongue or buccal mucosa with neck dissection  2. Between 1998 and 2015  3. The cases were selected from archives of the Department of Oral Pathology | 1. Depth of invasion  2. Tumor size  3. Pattern of invasion | "It provides a useful model that is practicable to use in developing countries of South and Southeast Asia where OSCC etiology is similar and advanced facilities are not available." |
| **43** | **Dik 2014** | 200 | Retrospective Cohort study | Netherlands | 62.2 ± 12.35 | 113(56.5) | OTSCC, 105(52.5) | T1 and T2 | Patients had primary surgery for a Stage 1–2 OSCC of the tongue, the floor of the mouth, or the cheek mucosa. | Margin status | "With this treatment strategy, the local recurrence rate was 4.5%. No evidence was found for local adjuvant treatment in the case of close margins P3 mm with 62 unfavorable histological parameters. Current data do not support the use of one treatment modality above any other." |
| **44** | **Dillon 2015** | 54 | Retrospective Cohort study | USA | 56.25 ± 19.04 | 40(74) | Tongue and Oral cavity | All | 1. Patients with OSCC  2. Treated with resection surgery at the University of Washington | Marginal status | "The results suggest that the presence of a close surgical margin (1 to 5 mm) is an adverse risk feature comparable to an involved margin and therefore, is associated with decreased disease-free and overall survival. Future studies are needed to replicate these findings before they can be used as a basis for clinical recommendations." |
| **45** | **Doll 2022** | 66 | Retrospective Cohort study | Germany | 65.8 ± 12.8 | 32(48.48) | Upper oral cavity, 66(100) | All | 1. All patients with primary SCC of the upper oral cavity  2. Received primary tumor resection in combination with ND as the initial treatment | Depth of invasion | "Elective neck dissection should be preferably performed for OSCC of the upper oral cavity. For early-stage and clinically node-negative patients, sentinel lymph node biopsy can be an alternative, especially in patients with a DOI ≤ 4.5 mm. Since this group had no occult CLNM below this DOI cutoff value, watch and might be an alternative for selected patients." |
| **46** | **Ourado 2020** | 254 | Retrospective Cohort study | Brazil | NR | 188(74) | 1. Tongue 2. Floor of mouth  3. Others | All | 1. Patients with OSCC  2. Treated at referral hospitals in Brazil | Tumor budding | "The TSR, tumour budding, and their combination provide significant information on OSCC outcome, suggesting that their incorporation in the routine evaluation of histopathological specimens might be useful in prognostication for OSCC patients." |
| **47** | **Ebihara 2019** | 64 | Retrospective Cohort study | Japan | 59.25 ± 19.33 | NR | OTSCC, 64(100) | Early | 1. Patients with cT1/2N0 tongue SCC  2. Underwent first curative surgery at our hospital  3. Between 2007 and 2015 | Tumor budding | "The sole predictor of NLM and the prognosis of early tongue SCC was TBG, indicating that it might help to select overwhelming risk patients." |
| **48** | **Ermer 2015** | 429 | Retrospective Cohort study | Germany | 58.3 ± 19.8 | 277(64.6) | OTSCC, 429(100) | All | 1. OSCC patients  2. Over a 13-year period | Tumor staging | "Increasing primary tumor size correlates with the incidence of cervical metastases and recurrence rate. Initial cervical metastases show no effect on recurrence rates. Differentiation of primary tumors does not correlate with the recurrence rate. The majority of recurrences show consistent histopathological grading." |
| **49** | **Faisal 2018** | 179 | Retrospective Cohort study | Pakistan | 57.92 ± 11.93 | 102(57) | OTSCC, 179(100) | T1 and T2 | 1. Patients with pathologically involved nodes (pN+) or close margin (<5mm)  2. Underwent post-operative radiotherapy(PORT)  3. With multiple involved levels or extranodal extension (ENE) received chemo-radiotherapy (CRT) | 1. Depth of invasion  2. Lymphovascular invasion  3. Degree of differentiation | "Depth more than 10 mm is associated with significantly increased risk of recurrence and nodal metastasis. Elective neck dissection should be a consideration for tumors having depth less than 5mm." |
| **50** | **Faustino 2021** | 87 | Retrospective Cohort study | Brazil | 59.4 ± 15.59 | 68(78.2) | Tongue and floor of mouth, 87(100) | T1,T2 | 1. Patients with primary OSCC 2. Underwent surgical treatment | Lymph Node Density | "The intra or peritumoral lymphatic vessel density had no predictive value for occult lymph node metastasis in the early stages of oral cancer arising in the tongue or floor of mouth." |
| **51** | **Federica 2020** | 36 | Retrospective Cohort study | Italy | 65.4 ±14.8 | 23(63.8) | 1. Tongue  2. Floor of the mouth  3. Buccal mucosa | T1 and T2 | 1. Age more than 18 years  2. History of intraoral US before biopsy for the assessment of suspected lesions  3. Confirmed diagnosis of primary OSCC located on soft tissues of the oral cavity | Tumor staging | "Intraoral US can provide useful information to determine the optimal treatment for patients with OSCC, particularly in early-stage tumors. Further studies of large cohorts of patients are necessary to develop and test a method that allows clinicians to measure DOI and assess error distributions accurately." |
| **52** | **Flörke 2021** | 331 | Retrospective Cohort study | Germany | 63.71 ± 21.17 | 226(64) | 1. Extraoral, 19(5.4)  2. Floor of the mouth, 99(28.3) 3. Tongue,74(21.1)  4. Mandibular alveolar process, 73(20.9)  5. Lower lip, 31(8.9)  6. Maxillary alveolar process, 19(5.4)  7. Regio buccalis, 20(5.7)  8. Soft and hard palate, 14(4)  9. Oropharynx, 1(0.3) | All | Patients with intraoral squamous cell carcinomas | Tumor staging | "Metastases of the contralateral side occur most frequently in SCCs of the palate and floor of the mouth. Furthermore, tumors with a high T status result in significantly higher rates of contralateral metastases. Similarly, the midline involvement, existing ipsilateral metastases, and the infiltration depth of the tumor had a highly significant influence on the development of lymph node metastases on the opposite side." |
| **53** | **Fu 2021** | 183 | Retrospective Cohort study | China | 57.75 ± 16.45 | 105 (57.4) | 1. Buccal mucosa, 33(18)  2. Tongue, 84(45.9)  3. Gingiva, 33(18)  4. Others 33 (18.0) | 1. T1, 66(36.1) 2. T2, 91(49.7) 3. T3, 14(7.7) 4. T4, 12(6.6) | 1. Patients with primary OSCC  2. Treated in the Department of Oral and Maxillofacial Surgery at Nanjing Stomatological Hospital | Perineural invasion | "PNI tends to be an active and continuous process in which tumor cells move far away from nerves to invade the nerve sheaths, which reflects clinically worsening survival. Therefore, the WPNI scoring system, which takes the highest score to refine the traditional PNI status, may be worth further clinical evaluation and promotion." |
| **54** | **Ganly 2013** | 164 | Retrospective Cohort study | Canada | 54.25 ± 16.45 | 90(55) | OTSCC, 164(100) | Early | 1. Patients with OTSCC  2. Received treatment at Memorial Sloan-Kettering Cancer Center   and Princess Margaret Cancer Center  3. From 1985 to 2005 was established | 1. Tumor thickness  2. Margin status  3. Lymphovascular invasion  4. Degree of differentiation  5. Perineural invasion | "Patients with low-risk, pathologic T1-T2N0 OTSCC had a greater than expected rate of neck failure, with contralateral recurrence accounting for close to 40% of recurrences. Failure occurred predominantly in patients who had primary tumors that were 4 mm thick." |
| **55** | **Goodman 2009** | 339 | Retrospective Cohort study | USA | a. <64, 214(63.13%)  b. >65, 125(36.87%) | 195(57.5) | OTSCC, 339(100) | All | 1. Patients who were diagnosed with OTSCC  2. Underwent tumor resection | 1. Depth of invasion  2. Lymphovascular invasion  3. Perineural invasion | "The current results indicated the importance of reporting tumor invasion characteristics for patients diagnosed with cancer of the oral tongue. The findings also underscore the need for continuous monitoring of adherence to the CAP protocol. " |
| **56** | **Grimm 2012** | 484 | Retrospective Cohort study | Germany | 60.3 ± 0.68 | 332(68.59) | 1. Lips  2. Palate  3. Buccal mucosa  4. Alveolar ridge  4. Floor of mouth | All | 1. Patients having undergone primary surgical resection for OSCC  2. Between January 1997 and December 2010  3. With complete (R0) resection were included in our study | 1. Depth of invasion  2. Tumor Grade | "This hospital-based retrospective cohort study points out different clinicopathological prognostic factors of survival in a large patient cohort treated for OSCC. It highlights increased tumor size and microvas cular invasion as the most independent prognostic factors in predicting survival in patients with OSCC." |
| **57** | **Gueiros 2011** | 63 | Retrospective Cohort study | Brazil | 59.55 ± 17.61 | 51(80.95) | OTSCC, 63(100) | All | 1. Biopsy-proven OTSCC No previous tumor  2. Treatment absence of distant metastasis (stage M0)  3. Surgery as the first treatment used  4. At least five years of follow-up | Tumor staging | "The Anneroth and Bryne grading systems seem to be capable of predicting the biologic behavior of OTSCC, but the Bryne system seems to be better than the Anneroth system in evaluating prognosis. Proliferation markers provide important information when predicting the prognosis of patients with OTSCC, so histopathological grading systems together with proliferation marker expression may be useful tools for evaluating the biological behavior of TSCC." |
| **58** | **Haidari 2022** | 226 | Retrospective Cohort study | Germany | 64.2 ± 10.9 | 136(60) | OTSCC, 226(100) | All | 1. Age older than 18 years  2. Primary diagnosis of OSCC  3. Follow-up of more than 60 months | 1. Perineural invasion  2. Lymphovascular invasion  3. Tumor Grade  4. Tumor stage | "The study conducted here shows that occult metastases lead to shorter progression-free survival compared to previously diagnosed metastases. High grading and a low T stage were more frequently associated with the occurrence of occult metastases. The data collected here argue against neck dissection in the case of a T1 or T2 tumor and a preoperative radiological N0 neck." |
| **59** | **Hakeem 2016** | 176 | Retrospective Cohort study | India | 51.75± 18.77 | 133(75.57) | OTSCC, 176(100) | All | Patients with a diagnosis of T1‑2 N0 SCC of oral tongue | 1. Depth of invasion  2. Lymphovascular invasion  3. Degree of differentiation  4. Perineural invasion | "We recommend prophylactic selective neck dissection in early-stage SCC of the oral tongue, especially with the depth of invasion more than 5 mm, perineural and lymphovascular invasion." |
| **60** | **Hamada 2023** | 58 | Retrospective Cohort study | Japan | 59.25 ± 19.33 | 43(77) | OTSCC, 58(100) | T1 and T2 | Patients with clinical stage I/II tongue SCC | Depth of invasion | "This study indicated that it is necessary to consider contraction due to specimen fixation by subtracting the thickness of the mucosal epithelium. Clinical T1 cases with a cDOI of 5 mm or less had a pDOI of 4 mm or less, and it would be expected to have a low positive rate of neck lymph node metastasis." |
| **61** | **Ho 2019** | 200 | Retrospective Cohort study | Taiwan | 57.2 ± 11.5 | 169(84.5) | 1. Tongue, 91(45.5)  2. Mouth floor, 8(4)  3. Buccal mucosa, 47(23.5)  4. Gingivae, 33(16.5)  5. Lip, 13(6.5)  6. Retromolar, 6(3)  7. Palate, 2(1) | 1. T1, 54 (27)  2. T2, 38 (19) 3. T3, 74 (37)  4. T4a, 23 (11.5)  5. T4b, 11(5.5) | 1. Patients with OSCC  2. Retrieved from the cancer registration database in Taipei Veterans General Hospital | Tumor budding | "TB is a significant predictor of tumor aggression with locoregional failure even in the revised 8th American Joint Committee on Cancer staging system." |
| **62** | **Hoda 2021** | 254 | Retrospective Cohort study | India | 24 to 74 | 105(41.33) | OTSC, 254(100) | All | 1. Patients with SCC of the buccal mucosa  2. Treated with surgery first approach | Degree of differentiation | "The majority of buccal mucosa cases showed metastasis to level I, II, and III lymph nodes, out of which level IB and/or IA was most frequently involved. Metastasis to level IV and V lymph nodes was rare and was seen especially in patients with advanced primary tumor and poor histopathological differentiation." |
| **63** | **Hoda 2018** | 56 | Retrospective Cohort study | South Korea | Mean(57.7) | 48(86) | OTSCC, 56(100) | 1. T1, 7(13) 2. T2, 27(48) 3. T3, 3(5) 4. T4, 19(34) | Patients with OSCC | Tumor budding | "In summary, we found that tumor budding was associated with lymph node metastasis and shorter overall survival in patients with OSCC, and similarly, a significant association was observed between the expression of Snail and Twist and worse patient outcomes. In addition, tumor budding was correlated with positive expression of EMT regulators. These findings suggest that tumor budding is significantly associated with poor prognosis in patients with OSCC and histologically represents an EMT process of OSCC." |
| **64** | **Hori 2020** | 62 | Retrospective Cohort study | Japan | 60 ± 16.16 | 43(69.4) | OTSCC, 62(100) | 1. T1N0M0, 35(56.45)  2. T2N0M0, 27(43.55) | 1. Patients were diagnosed with clinical early OTSCC  2. Between September 2000 and March 2017, in the Kanagawa Cancer Center | 1. Pattern of invasion  2. Lymphovascular invasion | "The pathological invasion patterns should be considered when determining the follow-up plan for patients with clinical N0 early oral tongue carcinoma." |
| **65** | **Hori 2017** | 48 | Retrospective Cohort study | Japan | 60 ± 16.16 | 33(69) | OTSCC, 48(100) | Early | 1. Patients with clinical early OTSCC  2. Of (cT1/2N0M0)  3. Underwent primary partial glossectomy | 1. Depth of invasion  2. Tumor budding  3. Lymphovascular invasion  4. Degree of differentiation | "Tumor depth and budding grade, as well as a combination of the two, were identified as histopathological risk factors for late neck recurrence in patients with clinical N0 early oral tongue carcinoma treated by primary surgery without END." |
| **66** | **Hori 2021** | 72 | Retrospective Cohort study | Japan | 60 ± 16.16 | 43(59.7) | OTSCC, 72(100) | Early | 1. Patients with early oral tongue SCC  2. Treated with only primary surgery | 1. Depth of invasion  2. Tumor budding  3. Lymphovascular invasion  4. Degree of differentiation | "In summary, each TIL subtype may use different mechanisms during early and advanced stages of oral tongue SCC. A high density of Tumor CD163+ macrophages was determined to be a risk factor for RC and DFS as well as an additional stratification factor for RC in patients with intermediate- or high-grade budding scores. Therefore, identifying TIL subtypes in daily clinical practice can help determine a more successful and individualized therapeutic approach for early oral tongue SCC." |
| **67** | **Hosni 2017** | 914 | Retrospective Cohort study | Canada | 58 ± 21.36 | 577(63.12) | 1. OTSCC, 419(46)  2. Others, 495(54) | All | 1. Primary pT1–4, pN0–2, M0 OSCC patients  2. At least 18 years of age  3. Underwent curative surgery with neck dissection (of 6 LNs)  4. Between 1994 and 2012 | 1. Depth of invasion  2. Growth pattern  3. Tumor size  4. Lymphovascular invasion  5. Degree of differentiation  6. Perineural invasion | "High LNR is associated with higher regional-only-failure/distant-only-failure and lower OS. LNR should be assessed in future prospective trials for selection of adjuvant therapy." |
| **68** | **Huang 2019** | 151 | Retrospective Cohort study | China | 50.5 ± 16.73 | 103 (68.2) | 1. OTSCC, 95(62.9)  2. Others, 56(37.1) | Early | 1. Primary clinical early-stage (cT1/2) OSCC  2. With a cN0 status on preoperative clinical examination and radiological tests  3. Surgical excision of the primary lesion and   concomitant elective unilateral or bilateral neck dissection  4. No previous Radiotherapy or chemotherapy  5. Complete clinicopathological data available | 1. Depth of invasion  2. Lymphovascular invasion  3. Degree of differentiation  4. Perineural invasion | "The results of this study suggest that the TSR is a crucial histological predictor of occult cervical lymph node metastasis and an independent prognostic factor for OS in early-stage (cT1/2N0) OSCC. " |
| **69** | **Imai 2016** | 67 | Retrospective Cohort study | Japan | 59.7 ± 19.34 | 41(61.19) | OTSCC, 67(100) | Early | 1. Patients with T1N0 OTSCC  2. Underwent primary treatment in our department  3. From April 2001 to March 2015 4. All the patients underwent watchful waiting alone for neck management | Depth of invasion | "Patients with tumors ≥2 mm in thickness or muscle invasion developed neck metastasis, suggesting that elective neck dissection may be warranted for patients with these findings. For preoperative assessment of the need for elective neck dissection, magnetic resonance imaging would be a potential modality for T1N0 tongue cancer." |
| **70** | **Jang 2016** | 295 | Prospective Cohort Study | South Korea | 57.5 ± 4.62 | 222(75) | 1. OTSCC, 174(58.98)  2. Oropharynx, 75(25.42)  3. Hypopharynx, 46(15.59) | 1. T1, 111(38)  2. T2, 111(38)  3. T3, 33(11)  4. T4, 40(14) | 1. HNSCC patients  2. Had undergone the initial curative surgery | 1. Degree of differentiation  2. Perineural invasion | "Both dimension and biology of primary tumors have a significant, tumor subsite-dependent impact on the occurrence of LNM in HNSCC. Particularly in oro- and hypo-pharyngeal cancers, tumor biological properties, which can be estimated through a biopsy or surgical pathology of primary tumors, could guide adjuvant treatment for regional lymph nodes. Meanwhile, the primary tumor dimension should be considered in the decision-making of neck management in oral tongue cancer; as such, an accurate evaluation of tumor dimension is important." |
| **71** | **Jang 2016** | 325 | Retrospective Cohort study | South Korea | NR | NR | 1. OTSCC  2. Floor of mouth  3. Buccal area | All | 1. Patients with pathologically proven OTSCC  2. Had undergone surgical resection as an initial treatment | Lymphoplasmacytic invasion | "The cSM5 was a significant risk factor for local recurrence only in advanced oral cancers, but not in early-stage tumors, where microscopic tumor extension was not beyond 3 mm in T1 tumors. Thus, the extent of surgical safety margin can be redefined according to the primary tumor size." |
| **72** | **Jangir 2021** | 120 | Prospective Cohort Study | India | Mean(47.2) | 104(87) | 1. Buccal mucosa, 36(30) 2. GBS, 19(15.83) 3. Lips, 14(11.6) 4. OTSCC, 51(42.5) | T1,T2 and T3 | Biopsy-proven Stage I–Stage III OSCC | 1. Depth of invasion  2. Growth pattern  3. Tumor size  4. Lymphovascular invasion  5. Degree of differentiation  6. Perineural invasion | "DOI is a significant predictor of cervical nodal metastasis, and tumor depth 5 mm can be considered as a cutoff value in staging and management of early oral squamous cell carcinoma." |
| **73** | **Jardim 2021** | 88 | Retrospective Cohort study | Brazil | 55.75 ± 15.87 | 64(72.7) | OTSCC, 88(100) | All | 1. Biopsy-proven Stage I–Stage III OSCC  2. Previously untreated patients without a second primary tumor  3. Submitted for treatment in the institution | 1. Lymphovascular invasion  2. Perineural invasion | "Our results suggest that high IT LVD has a strong impact on survival outcomes in advanced stage OSCC. " |
| **74** | **Jardim 2015** | 142 | Retrospective Cohort study | Brazil | Mean(57) | 109(76.8) | OTSCC and Floor of the mouth, 142(100) | T3 and T4 | 1. Patients with OSCC  2. Advanced stage (clinical stage III and IV)  3. Treated primarily with surgery at the cancer center in Sa˜o Paulo, Brazil 4. The patient had to have been submitted to treatment at the institution  5. Between 1998 and 2009 | 1. Lymphovascular invasion  2. Perineural invasion | "The presence of PNI in oral carcinoma surgical specimens has a significant impact on survival outcomes in patients with advanced stage tumors submitted to radical surgery and adjuvant radiotherapy/ radiochemotherapy." |
| **75** | **Jayasuriya 2020** | 187 | Retrospective Cohort study | Sri Lanka | 57.46 ± 9.68 | 135(72) | Tongue and Buccal mucosa, 187(100) | All | 1. Patients diagnosed with OSCC  2. Had a clinically (TNM) and pathologically (pTNM) positive necks  3. Over a period of 5 years (2013 January–2018 December) | 1. Lymphovascular invasion  2. Perineural invasion | "The need to routinely perform a modified radical/radical neck dissection for cN+s should be stopped as the incidence of Level V positivity is significantly low. Assessing the cN+ for the N stage, the status of levels III and IV, the pattern of invasion, differentiation, and the site may be used as predictors for level V positivity." |
| **76** | **Jensen 2015** | 199 | Retrospective Cohort study | Ireland | 59 ± 0.66 | 132(66.33) | OTSCC, 199(100) | 1. T1–T2, 169(84.92) 2. T3–T4, 30(15.08) | 1. Patients with pathologically proven OTSCC  2. Had undergone surgical resection as an initial treatment | 1. Depth of invasion  2. Tumor grade  3. Tumor budding  4. Margin status | "Given the strong relationship between tumor budding and the development of lymph node metastases and an adverse prognosis, therapeutics based on inhibiting the activation of TGF𝛃 signaling may prove useful in the treatment of OSCC." |
| **77** | **Jerjes 2010** | 115 | Retrospective Cohort study | UK | Mean(61.7) | 65(56.5) | 1. Tongue  2. Floor of mouth (FOM)  3. Buccal mucosa and alveolus | T1 and T2 | Patients who presented with T1/T2 oral squamous cell carcinoma (OSCC) | 1. Depth of invasion  2. Tumor stage | "Squamous cell carcinoma of the oral cavity has a poor overall prognosis with a high tendency to recur at the primary site and extend to involve the cervical lymph nodes. Several clinicopathological parameters can be employed to assess outcome, recurrence, and overall survival." |
| **78** | **Jia 2018** | 118 | Retrospective Cohort study | China | Mean(52) | 80(72) | Tongue and Floor of mouth | All | 1. Patients with SCC of the tongue and the floor of the mouth  2. Received treatment between 2012 and 2017 were included in this study | Degree of differentiation | "LLNs are rare in patients with SCC of the tongue and the floor of the mouth, and they would be ready to be omitted. The dissection of these LLNs would be of benefit to those patients with advanced pathological grade." |
| **79** | **Jones 2009** | 72 | Retrospective Cohort study | UK | 60.7 ± 13.1 | 51(71) | 1. Tongue  2. Floor of mouth  3. Buccal mucosa  4. RMT and lower alveolus | 1. T1, 21(29.167)  2. T2, 28(38.89) 3. T3, 5(6.94) 4. T4, 21(29.167) | Patients are treated primarily with resection and concomitant neck dissection for intraoral carcinomas. | Lymphoplasmacytic invasion | "This study raises the question of whether patients with evidence of lymphovascular invasion following primary surgical management should be selected for aggressive post-operative adjuvant therapy." |
| **80** | **Kakuguchi 2023** | 89 | Retrospective Cohort study | Taiwan | Mean(63) | 54(61) | OTSCC, 89(100) | T1 and T2 | 1. Patients diagnosed with clinical and pathological T1-2N0M0 (Stage I/II) tongue SCC  2. Patients were treated only with partial glossectomy  3. Not treated with chemotherapy or radiotherapy  4. Patients with their first episode of tongue SCC | 1. Depth of invasion  2. Tumor budding  3. Pattern of invasion  4. Degree of differentiation  5. Perineural invasion | "CLNM was observed in 25.8% of early-stage tongue carcinomas (Stage I/II). YK-4c and pDOI >5 mm were the most important CLNM risk factors identified. Close follow-up is needed after partial glossectomy when patients with tongue SCC have other risk factors, particularly YK-4c and pDOI >5 mm." |
| **81** | **Kallarakkal 2022** | 1098 | Retrospective Cohort study | Malaysia | NR | 748(68.12) | Oral tongue and Buccal mucosa | Early | 1. Patients who underwent surgery for treatment of OSCC of the tongue or buccal mucosa 2. With neck dissection was used for model development and validation | 1. Depth of invasion  2. Tumor size  3. Lymphovascular invasion | "The model based on established clinicopathological variables has been internally validated on a large cohort of patients and offers practicability for use in OSCCs of the tongue and buccal mucosa." |
| **82** | **Kane 2006** | 48 | Retrospective Cohort study | India | 21 to 90 | 37(77.1) | 1. Oral tongue, 40(83.33)  2. Buccal mucosa, 7(14.58) 3. Floor of the mouth, 1(2.08) | T1 and T2 | 1. Patients with OSCC  2. Previously untreated patients with clinically T1 or T2 and N0 3. Treated with primary excision of the tumor and elective neck node dissection were selected | 1. Depth of invasion  2. Tumor budding  3. Pattern of invasion  4. Degree of differentiation  5. Perineural invasion | "Depth is the most significant predictor of cervical node metastasis in early squamous carcinomas of the oral cavity. Patients with a tumor depth of more than or equal to 5 mm are at an increased risk of harboring node metastasis. Hence, they should be taken up for elective node dissection." |
| **83** | **Kapila 2017** | 40 | Retrospective Cohort study | India | a. <40, 21(52.5%)  b. >40, 19(47.5%) | 32(80) | Tongue and Buccal mucosa | All | 1. Histopathological diagnosis of OSCC  2. Availability of data (age and site of tumor) | 1. Degree of differentiation  2. Margin status | "Reasons for documented variability in tumor characteristics between young and older patients are currently unclear. The difference in AgNOR count found in the present study is suggestive of variability in proliferative and ploidy characteristics between different age groups and supports the hypothesis of genetic and epigenetic influences in the development of oral cancer." |
| **84** | **Kato 2023** | 25 | Retrospective Cohort study | Japan | Mean(60.2) | 9(36) | OTSCC, 25(100) | Early | 1. Patients with stage I/II TSCC  2. Had undergone surgical treatment without elective neck dissection | 1. Depth of invasion  2. Tumor budding  3. Pattern of invasion  4. Degree of differentiation  5. Perineural invasion | "By examining the combination of MOI and DOI or PNI, we believe that it will be possible to predict the development of late cervical lymph node metastasis more reliably. The present patients who had MOI type 4D, a DOI≥4 mm, or PNI are considered to be at high risk of late cervical lymph node metastasis, and elective neck dissection should be considered. Suppose a conservative observation approach is to be taken for such patients. In that case, strict follow-up including periodic image examinations is essential, and if cervical lymph node metastasis is suspected, an immediate response should be taken. It is expected that the prognosis of TSCC can be improved." |
| **85** | **khan 2017** | 58 | Prospective Cohort Study | Pakistan | 46.5 ± 4.62 | 48(82.8) | 1. Cheek/buccal mucosa  2. Tongue  3. Floor of mouth | 1. T1, 7(12.1)  2. T2, 23(39.7)  3. T3, 17(29.3)  4. T4, 11(19) | Patients with SCC of oral cancer | Tumor thickness | "CT scan of neck with contrast can be used for predicting the positive presence of lymph node in the neck with primary tumors having a size of more than 4 mm." |
| **86** | **Khwaja 2016** | 182 | Retrospective Cohort study | India | Mean(52.5) | 150(82.18) | 1. Buccal  2. Cheek  3. Floor of mouth and alveolar mucosa | All | Nonrecurrent OSCC having no previous history of any malignancy | 1. Growth pattern  2. Lymphoplasmacytic infiltration | "Factor that is primarily the manifestation of tumor and its microenvironment has taken the prime seat followed by the ones that are dictated by the tumor. The factors that are basically quantified were not able to show association. Site influences the nodal status alongside PI." |
| **87** | **Kim 1993** | 90 | Retrospective Cohort study | Japan | 59 ± 19.05 | 54(60) | OTSCC | T1 and T2 | 1. Patients with OSCC  2. Between January, 1973, and December, 1990 | 1. Lymphovascular infiltration  2. Degree of differentiation | "Although size, alone, helps in the very small and very large range, it is not sufficiently accurate for the prognosis." |
| **88** | **Kim 2018** | 368 | Retrospective Cohort study | USA | 62.3 ± 13.1 | 216(58.7) | Tongue and Floor of Mouth | All | 1. Adult subjects with primary SCC of the oral cavity  2. Underwent neck dissection | Perineural invasion | "Perineural invasion is statistically correlated with tongue and floor of the mouth subsites within the oral cavity, as well as larger tumors, deeper tumors, and disease which has progressed to the lymph nodes. Whether this correlation represents causation in any either direction remains unknown." |
| **89** | **Kos 2008** | 67 | Retrospective Cohort study | Germany | 60 ± 11 | NR | 1. Floor of the mouth  2. Tongue  3. Cheek  4. Palate  5. Base of the tongue  6. Oropharynx | All | 1. Patients with OSCC  2. Treated with surgery radiotherapy and their combination | Tumor staging | "A complete resection of the tumor was the most important independent prognostic parameter for the disease-free and overall survivals in oral squamous cell carcinomas in this study. Adjuvant radiation therapy could improve the results of treatment of oral squamous cell carcinoma also in cases were so far considered only for surgical management." |
| **90** | **Kowalski 2000** | 513 | Retrospective Cohort study | Brazil | 57.95 ± 20.5 | NR | 1. Oral tongue  2. Floor of the mouth  3. Lower gingiva  4. Retromolar trigone | T1,T2,T3 and T4 | Patients with squamous cell carcinoma of the oral cavity | Tumor stage | "The level of ipsilateral lymph node involvement was the most significant prognostic factor for patients with oral cancer who underwent surgical treatment. A significant decrease in survival was also seen with regard to the involvement of multiple contralateral lymph nodes. Our results support the indication of elective neck dissections in high-risk patients because, among the cases that had metastases at follow-up, 50% were not candidates for salvage treatment." |
| **91** | **Kurokawa 2002** | 50 | Retrospective Cohort study | Japan | 55.5 ± 19.34 | 27(54) | OTSCC, 50(100) | T1 and T2 | Patients with stage 1-2 oral cell carcinoma of the tongue | 1. Depth of invasion  2. Lymphovascular invasion  3. Pattern of invasion  4. Degree of differentiation | "The findings of this study demonstrated tumor depth more than or equal to 4mm moderately differentiated squamous cell carcinoma of the tongue that have substantially higher rate of late cervical metastases." |
| **92** | **Kurokawa 2005** | 124 | Retrospective Cohort study | Japan | 57.5 ± 19.34 | 79(63.7) | OTSCC, 124(100) | All | 1. Patients with OSCC  2. Previously untreated | 1. Depth of invasion  2. Tumor Grade | "This study found that a high IFG malignancy score had a high prognostic value for squamous cell carcinoma of the tongue." |
| **93** | **Lakhera 2022** | 100 | Prospective Cohort Study | India | 47.6 ± 17.31 | 75(75) | 1. Buccal mucosa, 38(38)  2. Tongue, 27(27)  3. Gingivobuccal sulcus, 19(19) | Early | 1. Patients with OSCC  2. Previously untreated | 1. Depth of invasion  2. Lymphovascular invasion  3. Degree of differentiation  4. Lymphoplasmacytic infiltration  5. Perineural invasion | "The presence of WPOI, LVI, and poor differentiation is an independent predictor of cervical nodal metastasis in early-stage oral cavity cancers. Identification of these histological parameters can help in clinical decision-making and prognostication of the disease. Also, the inter-correlation analysis among different histological parameters can help identify some high-risk factors that might not predict nodal metastasis independently. Still, it might have a significant effect on the outcome, survival, and overall prognosis of the disease. Our study, whilst offering innovative lines of inquiry, creates a paradigm for future insight on this topic." |
| **94** | **Larsen 2009** | 144 | Retrospective Cohort study | Denmark | 58 ± 21.93 | NR | Floor of mouth and lateral tongue | All | 1. Patients with OSCC  2. Previously untreated | 1. Depth of invasion  2. Tumor Grade | "Tumour depth and grade were strong prognostic factors for nodal metastasis, independently of other histological features. Tumor diameter and margins independently predict local recurrences in the oral cavity as well as cause-specific survival. Nodal involvement and ECS were associated with adverse prognosis." |
| **95** | **Lau 2021** | 88 | Retrospective Cohort study | Germany | 54.25±19.35 | 47(53) | OTSCC, 88(100) | 1. T1, 45(51) 2. T2, 23(26) 3. T3, 5(6) 4. T4, 15(17) | Patients who were treated surgically for tongue SCC | 1. Depth of invasion  2. Lymphovascular invasion  3. Degree of differentiation  4. Lymphoplasmacytic infiltration  5. Perineural invasion | "Margin status was the sole independent predictor of local recurrence in tongue SCC, and this risk was not eliminated with the administration of adjuvant therapy. The presence of nodal metastasis, DOI>6 mm, and positive margin status were independent prognostic factors for poor overall survival. Therefore, the presence of these adverse histopathologic factors may indicate the need for adjuvant therapy." |
| **96** | **Lee 2019** | 229 | Retrospective Cohort study | Taiwan | 55.19 ± 17.90 | 212(92.6) | OTSCC, 229(100) | All | 1. Patients with OCSCC  2. PNI who underwent radical surgery  3. Between July 2003 and November 2016 | Perineural invasion | "Compared with IPNI, the presence of EPNI in patients with OCSCC portends a less favorable prognosis and is an independent adverse predictor of 5‐year LRC, DFS, and OS rates. Patients with EPNI are potential candidates for definite aggressive treatment modalities (including adjuvant radiotherapy) aimed at improving clinical outcomes." |
| **97** | **Li 2019** | 161 | Retrospective Cohort study | China | a. <60, 93(57.76%) b. ≥60, 68(42.24%) | 108(67) | 1. Tongue, 87(54.04) 2. Buccal mucosa, 38(23.6)  3. Floor of mouth, 11(6.83) 4. Gingiva, 13(8.07) 5. Hard palate, 8(4.97(  6. Retromolar, 4(2.48) | 1. T1, 60(37.28)  2. T2, 77(47.83)  3. T3, 16(9.94) 4. T4, 8(4.97) | 1. Patients with OCSCC  2. Previously untreated  3. Patients with cN0 | 1. Depth of invasion  2. Degree of differentiation  3. Perineural invasion | "The growth pattern, degree of differentiation, depth of invasion, neutrophil/lymphocyte ratio, and the short/long axis diameter ratio of lymph nodes were the independent risk factors for pathological cervical lymph node metastasis in oral squamous cell carcinoma patients with cN0. If patients with the above risk factors receive nonstandard radical neck dissection or no dissection, it may be necessary for them to receive the corresponding regional postoperative radiotherapy." |
| **98** | **Lim 2004** | 56 | Retrospective Cohort study | South Korea | NR | 31(55.36) | OTSCC, 56(100) | T1 and T2 | 1. Patients with OSCC  2. Previously untreated | 1. Depth of invasion  2. Lymphovascular invasion  3. Degree of differentiation  4. Lymphoplasmacytic infiltration  5. Perineural invasion | "Our results indicate that patients with stage I and II invasive squamous cell carcinoma of the oral tongue with tumor thickness >4 mm, mode of invasion grade 3 or 4, and low expression of E-cadherin should be considered a high-risk group for late cervical metastasis when a wait-and-see policy for the neck is adopted." |
| **99** | **Lin 2020** | 2535 | Retrospective Cohort study | Taiwan | NR | 2416(95.3) | 1. Alveolar ridge  2. Anterior tongue  3. Buccal mucosa  4. Hard palate  5. Floor of mouth  6. Retromolar trigone  7. Lips | All | 1. Patients who were diagnosed with OSCC  2. Received treatment and follow-up at Changhua Christian Hospital  3. Between January 1, 2008 and December 31, 2018 | Tumor grade | "5% of patients in our study presented with poorly differentiated OSCC at diagnosis. Furthermore, grade 3 OSCC has a worse prognosis and is more aggressive than grades 1 and 2 OSCC. In the future, we should focus on modifying individual therapy for poorly differentiated OSCC to achieve improved outcomes." |
| **100** | **Liu 2016** | 322 | Retrospective Cohort study | Canada | 63.2 ± 14.9 | 181(56.2) | OTSCC, 322(100) | 1. T1, 230 (71.4)  2. Others, 92 (28.6) | 1. Patients diagnosed as having OSCC  2. From January 11, 2001, to December 24, 2007  3. Identified from the British Columbia Cancer Agency Registry | 1. Depth of invasion  2. Tumor grade | "Commonly used pathologic factors to decide neck dissection for cN0 OSCC are not effective and can cause overtreatment or undertreatment. The need for identification of new objective approaches for risk assessment of RF is urgent." |
| **101** | **Liu 2017** | 1383 | Retrospective Cohort study | Taiwan | 52.9 ± 11.1 | 1297(93.8) | Tongue and Oral cavity | 1. T1, 378 (27.3)  2. T2, 204 (14.8)  3. T3, 184 (13.3  4. T4, 617 (44.6) | 1. Patients with OSCC  2. Previously untreated | 1. Depth of invasion  2. Tumor grade | "The prognostic impact of pathological features from various subsites on the survival of OCSCC patients is not the same. First, PNI was associated with a poor prognosis with early-stage oral cavity SCC, especially in patients with primary at the tongue. In addition, LVI was related to a poor survival rate with late-stage OCSCC, especially in patients with primary at the buccal mucosa. Further investigation is warranted to validate our findings in a multicenter study. Grouping the different markers to establish a prognostic scoring system may provide a more accurate evaluation of the prognosis in OCSCC patients." |
| **102** | **Liu 2019** | 145 | Retrospective Cohort study | Australia | 60.5 ± 18.47 | 86(59) | OTSCC, 145(100) | Early | 1. Patients for histologically proven OTSCC (<4cm)  2. Treated between 1995 and 2012 | Depth of invasion | "TT and DOI were highly correlated with nodal risk but had different cut-points for prediction. Our findings highlight the need for these parameters to be recognized as discrete entities and reported appropriately. This study supports using the 5mm DOI, currently used for staging, as also the threshold value to guide elective nodal treatment." |
| **103** | **Lodder 2010** | 65 | Retrospective Cohort study | Netherlands | Mean(65) | 34(52.3) | Tongue and Oral cavity | T1 and T2 | 1. Patients with a T1-2 oral cavity cancer  2. Were seen at a tertiary referral center  3. Between 2004 and 2010 | Tumor thickness | "Tumour thickness is an important predictive marker for lymph node metastases. As such, it can help in decision-making with regard to the management of the primary tumor and neck. Based upon our findings, a wait-and-see policy is only warranted for superficial lesions with a tumor thickness of less than 7mm, but only if regular follow-up using US-guided aspiration of the neck is ensured." |
| **104** | **Loganathan 2016** | 71 | Retrospective Cohort study | UK | 58.5 ± 19.05 | 50(70.42) | Oral cavity and Oropharynx | T1/T2N0M0, 71(100) | 1. The 16 years from 2000 to 2016  2. Patients who were treated for stage I/II SCC   involving the anterior two-thirds of the tongue | Tumor thickness | "The results of this study support recent publications associating tumor thickness with nodal disease. Therefore, it is postulated that prophylactic neck dissection should be considered when the tumor thickness of anterior tongue SCC exceeds 5 mm in order to prevent lymphatic spread and improve survival rate." |
| **105** | **Lu 2022** | 595 | Retrospective Cohort study | Taiwan | 56.6 ± 17.9 | 539(90.58) | 1. Check mucosa  2. Gum  3. Tongue  4. Lip  5. Mouth floor  6. Retromolar trigone  7. Hard palate | All | 1. Patients newly diagnosed with OCSCC  2. At Chung Shan Medical University Hospital  3. Between January 2010 and December 2016 | 1. Lymphovascular invasion  2. Degree of differentiation  3. Perineural invasion | "Effective scoring models were established for predicting DM." |
| **106** | **Luksic 2016** | 85 | Retrospective Cohort study | Croatia | 59.5 ± 13.85 | 68(80) | 1. Floor of the mouth, 50(58.8) 2. Tongue, 20(23.5) 3. Retromolar space, 4(4.7) 4. Lower gingiva, 11(13) | Early | 1. Patients with clinically T1 to T2N0 OSCC  2. Primarily surgically treated between 2000 and 2004 | 1. Depth of invasion  2. Lymphovascular invasion  3. Degree of differentiation  4. Lymphoplasmacytic infiltration  5. Perineural invasion | "Large prospective investigations with reproducibility and the clinical translatability of immunohistochemical methods are needed in order to provide new and effective therapeutic strategies in the future." |
| **107** | **Madana 2015** | 116 | Retrospective Cohort study | Canada | 63 ± 18.47 | 66(66.9) | OTSCC, 116(100) | All | 1. Patients who presented to the Jewish General Hospital with a new diagnosis of OTSCC  2. During the period of January 2001-January 2013 | Tumor thickness | "Tumor thickness assessed by CT scan may provide an accurate estimation of true thickness and can be used in treatment planning." |
| **108** | **Mafra 2018** | 56 | Retrospective Cohort study | Brazil | 62 ± 15.6 | 39(69.6) | OTSCC, 56(100) | 1. T1/T2, 37(64.07) 2. T3/T4, 19(33.93) | Cases of primary OTSCC | Lymph Node Density | "The results highlight the role of intratumoral lymphatic vessels in tumor progression. Despite the importance of lymphangiogenesis for metastatic dissemination, the aggressiveness of OTSCC does not seem to be directly influenced by the number of peritumoral lymphatic vessels. Mast cells were not correlated with LVD nor with the clinicopathological parameters of OTSCC. These findings suggest that MCD is not a determinant factor for lymphangiogenesis or for the progression of OTSCC, indicating multiple pro- and antitumor effects of these inflammatory cells." |
| **109** | **Mair 2018** | 354 | Retrospective Cohort study | India | 50.25 ± 17.9 | 275(77.7) | 1. OTSCC, 168(47.5)  2. Others, 186(52.5) | 1. T1, 157(44.4) 2. T2, 197(55.6) | 1. Early oral cancer patients  2. All patients were clinically node-negative and biopsy-proven SCC  3. Operated at our hospital (a tertiary cancer center) from January 2012 to January 2014 | 1. Depth of invasion  2. Tumor size | "A significant number of patients with metastatic nodal size less than 1 cm have ECS, which suggests aggressive behavior of the primary tumor. Thus, elective neck dissection is the only way of detecting ECS in these patients, which may warrant treatment intensification." |
| **110** | **Manjula 2014** | 33 | Prospective Cohort Study | India | 26 to 84 | 9(27.3) | Gingivo Buccal Complex | 1. T1, 5(15.15)  2. T2, 14(42.42)  3. T3, 3(9.09) 4. T4, 11(33.33) | 1. Primary GBCSCC from the Department of ENT and Head and Neck Surgery  2. Bangalore Baptist Hospital from January 2012 to January 2014 | 1. Depth of invasion  2. Lymphovascular invasion  3. Degree of differentiation  4. Lymphoplasmacytic infiltration  5. Perineural invasion | "The results of our prospective study confirm the predictive value of worst POI for positive lymph nodes in patients with GBCSCC. Tumor thickness[5 mm and positive lymph nodes are independent negative prognostic factors. Because diffuse infiltrative/worst POI correlates with positive lymph nodes; it indirectly influences survival. Our classification of POI into two groups is less confusing and easy to reproduce, and it can be easily and rapidly analyzed from the routine H & E stained histopathological examination. Tumor budding had no statistically significant association with lymph nodal metastases or prognosis in GBCSCC." |
| **111** | **Marinelli 2020** | 52 | Retrospective Cohort study | USA | 65.8 ± 12.74 | 28(53.8) | Buccal SCC, 52(100) | All | Patients were diagnosed with conventional buccal SCC | 1. Depth of invasion  2. Pattern of invasion  3. Perineural invasion | "Older age, WPOI-5, and perineural invasion are significant prognosticators of worse OS. WPOI is associated with DOI, a finding which may have important implications for the pathogenesis and biologic behavior of the disease." |
| **112** | **Marktaylor 2010** | 21 | Prospective Cohort Study | Canada | 64 ± 8.66 | 12(57.14) | Tongue and floor of mouth | All | 1. Patients with oral carcinoma 2. All patients had biopsy-proven SCC of the tongue or floor of the mouth  3. Each patient received the usual standard of care, including Investigations, treatment and follow-up | Tumor thickness | "In oral cancer, tumor thickness is a significant predictor of cervical nodal metastasis. Our findings support preoperative ultrasonography as an accurate measure of maximal tumor thickness. In cases where this measure is equal to or greater than 5 mm, we would suggest the surgeon should strongly consider performing an elective neck dissection." |
| **113** | **Marzouki 2023** | 63 | Retrospective Cohort study | Saudi Arabia | 60 ± 16.16 | 34(54) | 1. Tongue, 39(61.9)  2. Buccal mucosa, 17(26.98)  3. Hard palate, 2(3.17)  4. Inferior alveolar ridge, 2(3.17)  5. Floor of the mouth, 2(3.17) 6. Superior alveolar ridge, 1(1.59) | All | 1. All patients diagnosed with OCSCC  2. Treated and followed up at King Abdulaziz University Hospital 3. Between January 2012 and December 2019  4. All patients who underwent surgical resection of the primary tumor | Perineural invasion | "According to the data in the present study, those patients with OCSCC who had an aggressive POI or the presence of PNI had worse clinical outcomes. Moreover, WPOI and PNI were found to be significant independent prognostic indicators for local tumor control and DFS. Therefore, follow-up plans for patients, especially those with early-stage OCSCC, should consider these pathological invasion patterns on surgical specimens. In addition, multimodal treatment is likely to benefit patients with early-stage oral SCC in whom aggressive high-risk disease is found by evaluating these factors. Based on the present findings, a multicentric analysis of pooled data is recommended for better clarity on this issue." |
| **114** | **Mascitti 2020** | 211 | Retrospective Cohort study | Itay | a. <65, 111(52.6%) b. ≥65, 100(47.4%) | 137(64.9) | OTSCC, 211(100) | 1. T1-T2, 111(52.6)  2. T3-34, 100(47.4) | 1. Primary OTSCC  2. Age over 18 years  3. No preoperative chemo or radiation therapy nor adjuvant chemotherapy  4. HPV infection  5. At least three years of follow-up for living patients | Lymphovascular invasion | "Including TSR in the predictive model could improve risk stratification of OTSCC patients and aid in treatment decisions." |
| **115** | **Matos 2014** | 74 | Retrospective Cohort study | Brazil | 58.1 ± 14.1 | 50(67.6) | OTSCC, 74(100) | T1 and T2 | Patients with early squamous cell carcinoma of the oral tongue | 1. Tumor grade  2. Tumor thickness | "Tumor thickness of greater than 7 mm is predictive of a higher incidence of lymph node metastasis, and a TT >10 mm is predictive of worse disease-free survival in SCC of the oral tongue." |
| **116** | **Matsui 2015** | 90 | Retrospective Cohort study | Japan | 60 ± 20.78 | 51(56.67) | OTSCC, 90(100) | T1 and T2-N0 | 1. Patients with T1-2 N0 SCC  2. Underwent primary excision as initial treatment without preoperative radiotherapy or chemotherapy | 1. Depth of invasion  2. Pattern of invasion | "The local control rate of tongue SCC in the early stages has significantly improved as a result of the development of surgical techniques. On the other hand, we believe that tumor depth of invasion ( 4 mm), POI, and VEGF-C expression all need to be considered in the preoperative and postoperative planning stages for tongue cancer treatment." |
| **117** | **Matsushita 2015** | 89 | Retrospective Cohort study | Japan | a. <64, 41(46.1%) b. ≥64, 48(53.9%) | 50(56.2) | OTSCC, 89(100) | All | 1. Patients who underwent radical surgery   for previously untreated OTSCC  2. Between January 2001 and December 2011 | Perineural invasion | "Perineural and vascular invasion are risk factors for regional metastasis and an adverse prognosis. In particular, perineural invasion has a strong relationship with the prognosis. We recommend that elective neck dissection be considered when perineural or vascular invasion is found in tumor samples. Perineural/vascular invasion in OTSCC" |
| **118** | **Melchers 2012** | 212 | Retrospective Cohort study | Netherlands | 60.5 ± 19.92 | 119(56) | 1. Tongue, 108(51) 2. Gum =15(7)  3. Floor of mouth= 64(30)  4. Cheek mucosa, 7(3)  5. RMT, 12(6) 6. Others, 6(3) | Early | 1. All pT1 and pT2 first primary oral tumors of which clinicopathologic data regarding nodal status were available   2. Diagnosed between 1997 and 2008 | Depth of invasion | "Infiltration depth is an independent predictor for the true N status in pT1–2 OSCC." |
| **119** | **Michikawa 2012** | 63 | Retrospective Cohort study | Japan | 56.2 ± 19.91 | 49(77.8) | OTSCC, 63(100) | 1. T1, 23(36.5) 2. T2, 31(49.2)  3. T3, 9(14.3) | 1. Primary oral tongue SCCs (OTSCCs)  2. Between June 1999 and April 2008 3. Evaluated VI status by investigating lymphatic vessel invasion (LVI) and blood vessel invasion (BVI) by using immunohistochemistry (IHC) with monoclonal antibody | Lymphovascular invasion | "LVI at the primary site evaluated by IHC with D2-40 was significantly associated with cervical lymph node metastasis. In addition, the presence of BVI evaluated by EVG staining correlated with poor outcome in OTSCC patients. Evaluation of VI status using IHC with D2-40 and EVG staining may be useful for predicting lymph node metastasis and poor prognosis in OTSCC cases. To establish a standardized staining protocol and reveal the clinical significance of both LVI and BVI in patients with OTSCC, a larger multicenter prospective study is required." |
| **120** | **Mijatov 2023** | 65 | Prospective Cohort Study | Serbia | 59.65 ± 9.42 | 53(81.5) | 1. Tongue, 32(49.2)  2. Floor of the mouth, 22(33.8)  3. Hard palate, 4(6.2)  4. Gingiva, 4(6.2)  5. Buccal mucosa, 3(4.6) | 1. T1, 8(12.3)  2. T2, 29(44.6)  3. T3, 27(41.5)  4. T4, 1(1.5) | 1. Patients who were surgically treated for oral cancer  2. Between January 2013 and December 2015 at the Clinic for maxillofacial surgery | Depth of invasion | "PTV was an important pathological prognostic factor for survival in patients with OSCC." |
| **121** | **Mneimneh 2021** | 147 | Retrospective Cohort study | USA | 32 ± 6.91 | 89(59) | 1. OTSCC, 131(87)  2. Gingiva 9(6.12)  3. Buccal mucosa, 8(5.44)  4. Lip, 2(1.36) | All | 1. Patients with OSCC  2. At the age of 40years or younger | 1. Perineural invasion  2. Lymphovascular invasion | "Our study showed that OSCCY most commonly occurred in non-smokers and did not always occur in the setting of known genetic predisposition syndromes. FA was found to be associated with OSCC in 5% of the cases with the gingiva being the most frequent site of the tumor. Young age (30 years or less) was found to be an independent prognostic factor for worse OS and DSS, while histologic grade was an independent prognostic factor for DSS. Additional studies of OSCCY are still needed for a better understanding of this disease in young patients, and to investigate its molecular and pathophysiologic mechanisms." |
| **122** | **Morand 2019** | 92 | Retrospective Cohort study | Switzerland | 58 ± 13 | 62(67.4) | OTSCC, 92(100) | Early | 1. Patients with histologically proven OSCC  2. With small primary tumors (T1-T2) without  evidence of neck disease (cN0) on ultrasonography with fine   needle aspiration biopsy and/or on CT and/or MRI | Depth of invasion | "CD44 expression correlates with DOI, which predicts occult lymph node metastasis. Preoperative CT and/or MRI provides an accurate estimation of histopathological DOI. Both pieces of information gained preoperatively can help surgeons tailor their operation in regard to the surgical management of the neck." |
| **123** | **Morimoto 2006** | 27 | Retrospective Cohort study | Japan | 57 ± 17.31 | 15(55.55) | 1. Tongue  2. Lower gingiva  3. Upper gingiva  4. Floor of mouth  5. Buccal mucosa | All | 1. Patients with SCC of the oral cavity  2. Underwent radical neck dissection at Kyushu Dental College hospital  3. Between 1987 and 2000 | Degree of differentiation | "The results indicated that if a patient had SCC with low-grade differentiation, CNN in small lymph nodes would be difficult to detect on CT scan. Therefore, noting changes in lymph node density in the absence of CNN on CT scans is necessary in case the primary tumour is lowgrade SCC." |
| **124** | **Mucke 2011** | 334 | Retrospective Cohort study | Germany | 60.3 ± 12.14 | 224(67.1) | 1. Tongue  2. Alveolar crest  3. Floor of mouth  4. Buccal region | 1. T1, 95(28.4) 2. T2, 108(32.3)  3. T3, 25(7.5)  4. T4, 106(31.7) | 1. Patients with OSCC  2. Treated during 1992–2006  3. All patients were previously untreated and underwent mandibulectomy   for histologically proven oral squamous cell carcinoma | Depth of invasion | "If bone invasion is identified histologically in a resected specimen, the prognosis is not worsened and additional surgery need not be undertaken in adequately resected margins. Although the mandible should be preserved if feasible, the choice of treatment should always provide safe resection margin. The high rates of unsuspected bone invasion found in this study should be kept in mind in patients with OSCC close to the mandible." |
| **125** | **Mücke 2016** | 492 | Retrospective Cohort study | Germany | 59.14 ± 12.13 | 329(66.9) | OTSCC, 492(100) | 1. T1, 207(42.07) 2. T2, 159(32.32)  3. T3, 29(5.89)  4. T4, 97(19.72) | 1. All patients undergoing radical tumor resection for OTSCC  2. Between 2000 and 2012 | Tumor Thickness | "We highlight the importance of tumor thickness as a predictive variable in tongue cancer. Specifically, a cut-off point of 8 mm allowed for a more accurate and statistically precise prediction of lymph node metastasis. These findings could supplement the current classification of tongue cancers and form the basis for treatment." |
| **126** | **Muhammad 2021** | 80 | Retrospective Cohort study | Pakistan | 49.7 ± 14.1 | 49(61.25) | OTSCC, 80(100) | 1. T1, 34(42.5) 2. T2, 46(57.5) | 1. Patients presenting with early-stage OTSCC  2. Patients with tumor size of ≤4 cm  3. Underwent elective neck dissection at the time of surgery | Depth of invasion | "Occult neck node metastasis is significantly associated with the DOI. The risk of neck metastasis is higher in patients with a DOI >5 mm." |
| **127** | **Muttagi 2016** | 106 | Retrospective Cohort study | India | 51.95 ± 11.72 | 51.95 ± 11.72 | Mandibular Gingivo-Buccal sulcus, 106(100) | 1. T3, 38(35.84) 2. T4, 68(64.15) | 1. Patients comprised biopsy proven T3/T4 SCC of mandibular GBS  2. Underwent unilateral surgery at our center  3. Between January 2012 and October 2014 | Lymph Node Density | "With the single surgeon, pathologist and same surgical procedure, the mean LNY in Indian patients with T3/T4 SCC of mandibular GBS is 21.97 ± 5.57. Although clinicopathological factors affect the estimation of LNY, further studies are needed to validate the findings of this study." |
| **128** | **Naha 2023** | 63 | Retrospective Cohort study | USA | 61.5 ± 13.3 | 45(71.4) | Tongue and Floor of the mouth | All | 1. Patients with histologically proven oral cavity SCC  2. Between January 2014 and July 2019  3. Underwent preoperative contrast-enhanced CT and/or PET/CT  4. Pathological assessment of DOI was determined by a review of pathology reports | Depth of invasion | "DOI measurement is feasible with routine preoperative CT and PET/CT images and is comparable to pathological measurement in patients with oral cavity SCC." |
| **129** | **Nair 2021** | 24 | Prospective cohort study | India | 52 ± 15.58 | 16(66.7) | OTSCC, 24(100) | Early | The patient population of any age or sex with:  1. Biopsy proven T1N0 or T2N0 primary OTSCC  2. Tumors located on lateral tongue in anterior two-third | Tumor thickness | "Ultrasonographic evaluation is reliable and cost‑effective tool to measure the TT preoperatively, which will be of help in deciding the management in early OTSCC. TT of 4 mm and above was predictor of occult cervical nodal metastasis." |
| **130** | **Nair 2018** | 1524 | Retrospective Cohort study | India | 51.25 ± 19.34 | 1226(80.4) | 1. OTSCC, 533(34.77) 2. Buccal mucosa cancers, 991(65.02) | 1. T1, 337(22.1) 2. T2, 559(36.7) 3. T3, 114(7.5) 4. T4, 514(33.7) | 1. Patients with oral cavity SCC  2. Underwent surgery from January 2012 to March 2015 was conducted | Perineural invasion | Aggressive treatment of the primary cancer with the coincident management of the neck is important in the presence of PNI. The PNI worsens survival and warrants intensification of adjuvant treatment." |
| **131** | **Nayanar 2019** | 160 | Retrospective Cohort study | India | a. 30-44, 12(7%) b. 45-59, 46(29%) c. 60 and above, 102(64%) | 106(66) | 1. OTSCC, 63(39) 2. Buccal mucosa, 80(50)  3. Others, 17(24) | 1. T1, 22(14) 2. T2, 48(30) 3. T3, 38(24) 4. T4, 52(32) | Cases of oral squamous cell carcinoma | 1. Depth of invasion  2. Lymphovascular invasion  3. Degree of differentiation  4. Lymphoplasmacytic infiltration  5. Perineural invasion | "The clinicopathologic parameters such as site of cancer (P = 0.03), histologic differentiation (P = 0.03), shape of rete pegs (P = 0.002), pattern of invasion (P = 0.0001) and depth of invasion >3 mm (P = 0.016) were significantly associated with the risk of lymph node metastasis. The risk score devised based on these predictors serves as an efficient tool in aiding clinical decision-making regarding the extent of neck dissection." |
| **132** | **Nguyen 2021** | 70 | Retrospective Cohort study | Newzeland | 62.75 ± 17.60 | 40(57.1) | OTSCC, 70(100) | T1N0M0 | 1. Patients with stage I oral SCC at Waikato Hospital, New Zealand  2. Between 2008 and 2018 | Depth of invasion | "There is little data published for management outcomes of the node-negative neck in stage I oral squamous cell carcinoma. Given salvage neck dissection carries a poorer prognosis, END should be recommended for all T1N0 oral SCC with DOI $ 3 mm. In cases of DOI < 3 mm undergoing primary ablation only, a staging neck dissection as a second procedure should be considered in the presence of poor tumor differentiation or PNI on final histology." |
| **133** | **Niu 2016** | 207 | Retrospective Cohort study | China | 57.25 ± 20.48 | 121(58.5) | Mandibular gingiva, 207(100) | 1. T1, 37(17.9) 2. T2, 81(39.1) 3. T3, 22(10.6) 4. T4, 62(30) 5. Missing, 5(2.4) | 1. Patients with primary MGSCC  2. From January 2000 to September 2009 | Tumor stage | "MGSCC has unique clinical and pathological characteristics. MGSCC is not aggressive, and the survival outcomes of MGSCC are better than those of SCC at other sites. It is suggested that patients with T1 tumours are subject to a ‘wait and watch’ policy for the neck, while patients with T2–T4 tumours undergo elective neck dissection. Pathological neck metastasis, perineural invasion, and ECS were the most significant predictive factors of the 5-year overall survival rate. This study establishes a risk model based on six clinical and pathological prognostic factors, which might be of clinical benefit in classifying patients into low-, moderate-, and high-risk groups. It is suggested that these prognostic factors should be regularly shown in pathology reports." |
| **134** | **Noda 2022** | 186 | Retrospective Cohort study | Japan | 64.95 ± 17.31 | 115(61.8) | 1. Palate 4(2.15)  2. Oral floor, 3(1.61) 3. Gingiva 29(15.59)  4. Tongue, 129(69.35)  5. Buccal mucosa, 21(11.29) | All | 1. Patients with primary OSCC  2. With no clinical history of SCC   in other parts of the body and  3. Had not received any therapy before surgery | 1. Tumor budding  2. Lymphoplasmacytic infiltration | "The tumor microenvironment status in primary OSCC was signifcantly associated with that of ENE, and TB-H was an independent risk factor for ENE. The histological status of DR-I/TILs-L/cDOI>10 mm in biopsy specimens and TB-H/pDOI>10 mm in resection specimens is a useful predictor of ENE." |
| **135** | **Nomura 2009** | 33 | Retrospective Cohort study | Japan | 63 ± 13.4 | 23(69.7) | 1. Tongue, 17(51.52)  2. Gingiva, 11(33.33)  3. Oral floor, 1(3.03) 4. Buccal mucosa, 4(9.09) | 1. T1, 7(21.21)  2. T2, 6(18.18) 3. T3, 8(24.24) 4. T4, 12(36.36) | Thirty-three pairs of typical keratinizing-type OSCC samples | 1. Depth of invasion  2. Lymphovascular invasion  3. Degree of differentiation  4. Lymphoplasmacytic infiltration  5. Perineural invasion | "Our study provides the first documentation that human ATG16L1 protein is expressed heterogeneously in several subcellular components in both OPLs and OSCCs and that positive stromal ATG16L1 expression is correlated with lymphovascular invasion of tumor cells and lymph node metastasis in patients with OSCC. Our findings suggest a potential role of ATG16L1 in OSCCs. However, the ultimate molecular mechanisms linked to the dysregulation and aberrant subcellular localization of ATG16L1 in OSCCs remain unclear. Further studies are warranted to elucidate the molecular alterations involved in ATG16L1 expression in oral carcinogenesis." |
| **136** | **Nseir 2019** | 104 | Retrospective Cohort study | Israel | Mean(60) | 50(48.07) | 1. Tongue, 71(68.3)  2. Base, 13(12.5)  3. Ventral surface, 13(12.5) 4. Dorsal surface, 5(4.8)  5. Tip, 3(2.9)  6. Other extraglossal, 14(13.5) | All | 1. Patients with OSCC  2. Previously untreated | Tumor stage | "In addition to other previously known prognostic factors, LN density may serve as a strong prognostic factor for survival and recurrence in patients with advanced- and earlystage OTSCC." |
| **137** | **O’Brien 2003** | 145 | Prospective cohort study | Australia | 61.5 ± 17.89 | 98(67.5) | OTSCC, 72(100) | 1. T1, 62(42.76)  2. T2, 83(57.24) | 1. Patients of T1, and T2 with oral cavity cancer  2. Treated in the Department of Head and Neck Surgery, Royal Prince Alfred Hospital, Sydney | 1. Tumor thickness  2. Lymphoplasmacytic infiltration | "Tumor thickness is a highly significant, objectively measurable prognostic factor in early stage oral cancers. There is a need to standardize techniques of measurement to allow a multi-institutional study to be carried out. This will facilitate the development of strategies aimed at improving the outcome of higher risk patients." |
| **138** | **O-charoenrat 2003** | 50 | Retrospective Cohort study | UK | 57.25 ± 14.71 | 31(62) | OTSCC, 50(100) | 1. T1, 13(26) 2. T2, 37(74) | 1. Between January 1981 and December 1998  2. Patients with previously untreated Stage I/II OTSCC  3. Treated at the Head and Neck Unit, Royal  Marsden Hospital was reviewed. | Depth of invasion | "The evaluation of the tumour thickness in early oral tongue cancer at the time of presentation may allow the identification of a subset of patients who are more susceptible to metastatic spread via lymphatic pathways and permit therapy to be offered accordingly." |
| **139** | **Okada 2003** | 38 | Retrospective Cohort study | Japan | NR | NR | 1. Tongue  2. Lower gingiva  3. Oral floor  4. Buccal mucosa  5. Upper gingiva | All | 1. Patients with oral squamous cell carcinoma  2. Treated at our department from 1993 to 1997 | Lymphoplasmacytic infiltration | "When the sum of the degree of histological malignancy exceeds 15, metastasis in the cervical lymph nodes should be considered." |
| **140** | **Okada 2010** | 30 | Retrospective Cohort study | Japan | 55.85 ± 15.58 | 18(60) | 1. Tongue  2. Oral floor  3. Maxillary gingiva  4. Mandibular gingiva  5. Buccal mucosa  6. Palate | 1. T1, 5(16.67)  2. T2, 10(33.33) 3. T3, 13(43.33) 4. T4, 3(10) | 1. Patients with primary OTSCC  2. Treated at our hospital during the past ten years | 1. Tumor thickness  2. Lymphoplasmacytic infiltration | "The present study suggested that histological malignancy grade and tumor blood vessel density can be predictive factors for CLN metastasis in tongue SCC. Also, the results indicated that patients with tongue SCC showing Anneroth’s histological malignancy grade14 of 16 or more, tumor blood vessel density of more than 37, and a finding of lymphatic invasion by tumor cells should be considered a high-risk group for CLN metastasis and should be treated with the utmost attention." |
| **141** | **Okuyama 2018** | 25 | Retrospective Cohort study | Japan | 75 ± 8.7 | 13(52) | Tongue is divided into: 1. Anterior, 2(8)  2. Posterior, 14(56)  3. Both, 9(36) | 1. cT1-3, 3(12) 2. cT4, 22(88) | 1. Patients with mandibular invasive OSCC patients  2. Underwent marginal or segmental or hemi-mandibulectomy  3. Without neoadjuvant chemotherapy or radiotherapy  4. Between January 2010 and December 2016  5. All patients were followed up over one year after surgery | 1. Lymphovascular invasion  2. Degree of differentiation  3. Perineural invasion | "The present study first revealed that the CD44v6 expression and the amount of tumor budding can be used as prognostic markers of CLNM. The results basically supported the recommendation of adjuvant treatment by the NCCN Guideline. After adjuvant treatment, such cases continuously need frequent and strict follow-up using several modalities over a long period. To evaluate further applications of those factors and to determine definite cut-off points of IHC and the number of tumor buds, it will be necessary to conduct further studies of the mandibular invasive OSCC in the future." |
| **142** | **Oneyama 2009** | 46 | Retrospective Cohort study | Japan | 61.2 ± 10.9 | 27(58.7) | 1. Tongue, 17(36.96)  2. Lower gingiva, 13(28.26)  3. Oral floor, 5(10.87) 4. Buccal mucosa, 9(19.57) 5. Upper gingiva, 2(4.35) | 1. T1, 9(19.6)  2. T2, 21(45.7)  3. T3, 9(19.6)  4. T4, 7(15.2) | Patients with primary OSCC were examined and graded histologically | Tumor stage | "Histological grading of malignant primary tumors may predict cervical lymph node metastasis. However, positive findings are influenced by multiple factors, ranging from p53 positivity to the density of microvessels. Further detailed study of the association between these factors is warranted." |
| **143** | **Ong 2015** | 99 | Retrospective Cohort study | Australia | 60.25 ± 20.49 | 56 (56.6) | OTSCC, 99(100) | 1. T1, 39(39.3) 2. T2, 44(44.4) 3. T3, 8(8.1) 4. T4, 6(6.1) 5. Unknown, 2(2) | Patients with primary SCC of the tongue treated by surgery | Lymph Node Density | "Our data suggest that lymph node density is a reliable and applicable predictor of prognosis in patients with tongue SCC." |
| **144** | **Somya 2020** | 40 | Retrospective Cohort study | Denmark | >45, 31(77.5%) | 16(40) | 1. Buccal mucosa, 30(75)  2. Tongue, 6(15)  3. Alveolus, 4(10) | All | 1. Patients with histopathologically confirmed primary OSCC  2. Had undergone surgical resection with neck dissection  3. From 2014 to 2017 | 1. Depth of invasion  2. Lymphovascular invasion  3. Pattern of invasion  4. Tumor body | "Metastasis to the lower neck, and local recurrence were risk factors of DM in oral cancer patient." |
| **145** | **Pandit 2023** | 462 | Retrospective Cohort study | India | 50.75 ± 19.64 | 407(88.1) | 1. Lip, 9(1.95) 2. Hard palate, 4(0.866)  3. Alveolus, 10(2.16)  4. Buccal mucosa, 228(49.35)  5. Tongue, 207(44.81) 6. Floor of mouth, 3(0.65) | Early | 1. Oral squamous cell carcinoma (OSCC) patients  2. Presented to our centre from January 2014 to December 2021 | 1. Depth of invasion  2. Tumor grade  3. Lymphovascular invasion  4. Perineural invasion | "Reporting and studying the clinico-pathological features of primary tumors can give vital information in predicting the neck node metastasis in OSCC patients." |
| **146** | **Patel 2009** | 356 | Retrospective Cohort study | Canada | 63.5 ± 20.78 | 226(63) | 1. Tongue  2. Alveolus  3. Floor of mouth  4. Buccal mucosa  5. Retromolar | 1. T1, 108(32.14) 2. T2, 132(39.29) 3. T3, 42(12.50 4. T4, 74(22.02) | Patients with oral carcinoma treated at the Sydney Head and Neck Cancer Institute | Tumor stage | "Thickness is an independent predictor of nodal metastases and disease-related death in oral cavity carcinomas, and its position as an important clinicopathological prognostic factor is now well established. The risk of occult metastases in oral carcinoma is high enough to consider elective dissection for all patients with tumors greater than 2 mm thick. Levels I–III should be dissected routinely, and levels IV and V should only be encompassed in patients with clinically evident nodal disease at other levels." |
| **147** | **Pedersen 2015** | 253 | Retrospective Cohort study | Denmark | 62.75 ± 18.76 | 145(57) | 1. FOM  2. Tongue  3. Buccal mucosa | 1. T1, 170(67) 2. T2, 83(33) | 1. All patients diagnosed with cT1 to T2N0  2. Underwent a diagnostic SLNB between 2007 and 2013 | 1. Depth of invasion  2. Tumor stage  3. Lymphovascular invasion  4. Degree of differentiation  5. Perineural invasion | "These data support the use of the SLNB technique as an accurate and safe staging tool in patients with OSCC with a cN0 neck." |
| **148** | **Perisanidis 2013** | 97 | Retrospective Cohort study | Austria | a. <60, 64(65.98%)  b. >60, 33(34.02%) | 67(69) | OTSCC, 97(100) | 1. T3, 9(9)  2. T4, 88(91) | 1. Patients with primary locally advanced OSCC  2. Treated with neoadjuvant chemoradiotherapy followed by locoregional resection  3. At the Medical University of Vienna, between 2001 and 2009 | Lymph Node Density | "We provide evidence that a high pretreatment NLR is a significant independent predictor of poor cancer-specific survival in patients with advanced oral cancer receiving preoperative chemoradiotherapy. Further studies are warranted to validate our results and to determine whether circulating markers of cancer-related inflammation might represent novel therapeutic targets in oral cancer." |
| **149** | **Petrovic 2016** | 326 | Retrospective Cohort study | USA | Mean(63.5) | 192(59) | 1. Buccal mucosa, 50(15.34) 2. Floor of mouth, 113(34.66) 3. Gum, 107(32.82) 4. RMT, 34(10.43) 5. Tongue, 22(6.75) | 1. T1, 94(28.83) 2. T2, 164(50.31) 3. T3, 34(10.43) 4. T4, 28(8.59) 5. Unknown, 6(2) | 1. Previously untreated patients who underwent   marginal mandibulectomy for oral cavity SCC  2. Between the years 1985 and 2012 | Bone invasion | "Microscopic bone involvement does not adversely influence outcomes, but medullary bone involvement does confer a higher risk of positive bone margins. MM and appropriate adjuvant treatment is an effective strategy for the treatment of OCSCC in selected patients with primary tumors adherent to or proximity to the mandible." |
| **150** | **Monevska 2013** | 75 | Retrospective Cohort study | Macedonia | NR | NR | OTSCC, 75(100) | All | 1. Patients with OSCC  2. All of whom were hospitalized at the clinic for maxillofacial surgery  in Skopje in five years  3. Limited to patients with II, III, and IV clinical-stage oral carcinoma | Depth of invasion | "Tumour depth, vascular invasion, and mode of invasion are the most reliable predictive factors for subclinical nodal metastases. A malignancy grading system based on significant histological parameters is helpful in everyday practice for selecting patients with a high risk of occult metastasis and predicting the aggressive behavior of the tumor." |
| **151** | **Rahman 2021** | 85 | Retrospective Cohort study | UK | 63.4 ± 13.3 | 54(64) | OSCC,85(100) | 1. T1, 34(40)  2. T2, 51(60) | 1. Patients treated for primary OSCC  2. With primary excision, including neck dissection  3. Diagnosed between 2009 and 2014 | 1. Depth of invasion  2. Tumor stage  3. Lymphovascular invasion  4. Degree of differentiation  5. Perineural invasion | "Risk assessment of low-stage oral squamous cell carcinoma primary tumors may be predictive of the presence or absence of metastasis at presentation. Knowledge of the  Risk score and its constituent parts may inform treatment decisions at multidisciplinary meetings. Low-risk squamous cell carcinoma may be a rare variant with low metastatic potential and excellent long-term survival." |
| **152** | **Reddy 2018** | 35 | Retrospective Cohort study | India | 52 ± 17.61 | 30(85.7) | Buccal MucosA, 35(100) | 1. T1, 7(20) 2. T2, 28(80) | 1. SCC cases (T1/T2)  2. At buccal mucosa site | 1. Depth of invasion  2. Tumor stage  3. Lymphovascular invasion  4. Degree of differentiation  5. Perineural invasion | "TT is a highly significant, objectively measurable prognostic factor in early-stage oral cancers, and elective neck therapy is indicated for tumors exceeding 1.5 mm invasion." |
| **153** | **Rhutso 2022** | 80 | Retrospective Cohort study | India | a. >40, 66(82.5%) b. <40, 14(17.5%) | 57(71.3) | OTSCC, 80(100) | 1. T1, 6(7.5) 2. T2, 27(33.8) 3. T3, 26(32.5) 4. T4, 21(26.3) | 1. OTSCC cases 2. From January 2015 to December 2017  3. Studied by two pathologists for different patterns using the Brandwein-Gensler scoring system | Pattern of invasion | "We concluded from our study that worst POI is significantly associated with number of lymph nodes metastasis and perineural infiltration and hence can be used as an independent prognostic factor." |
| **154** | **Safi 2017** | 518 | Retrospective Cohort study | Germany | 62 ± 21.07 | 302(58.4) | 1. Floor of mouth, 168(32.5)  2. Tongue, 126(24.37)  3. Mandible, 97(18.76) 4. Maxilla/hard palate, 58(11.22)  5. Soft palate, 11(2.13) 6. Cheek, 57(11.03) | All | 1. Patients with treatment-naive OSCC  2. Primarily curative intended surgery, based on radical tumor resection   and neck dissection, with negative resection margins | Degree of differentiation | "Within the analyzed clinicopathological characteristics, histological grading has been demonstrated as an independent risk factor for locoregional recurrence in multivariate. Furthermore, univariate analysis indicates the number of resected and positive lymph nodes, postoperative radiation, and extracapsular spread as significant risk factors. Taking these results into account, the mentioned parameters, especially histological grading, need to be considered for the individualized therapy management of patients with OSCC." |
| **155** | **Safi 2018** | 89 | Retrospective Cohort study | Germany | 63.37 ± 11.91 | 59(66.28) | OTSCC, 89(100) | All | 1. Patients who were diagnosed with OSCC  2. Treated between 2002 and 2013 at our Department for Oral and Craniomaxillofacial Plastic Surgery | 1. Lymphovascular invasion  2. Degree of differentiation  3. Perineural invasion | "LNR predicted locoregional recurrence better than the conventional nodal staging system and, therefore, might serve as a more precise risk stratification tool. LNR > 7% led to an 11.419-fold higher risk for locoregional recurrence of patients with mandibular infiltration due to OSCC." |
| **156** | **Sagowski 2004** | 115 | Retrospective Cohort study | Germany | NR | NR | Oral cavity and OSCC | All | 1. Patients treated for primary OSCC  2. With primary excision, including neck dissection | Depth of invasion | "In squamous cell carcinomas of the oral cavity and the oropharynx features of the lymph node metastasis such as the capsular rupture generally have a greater prognostic impact than features of the primary tumor. It is not the difference between N0 and N+, but the differentiation between intra- and extracapsular spread of the lymph node contains the essential discriminatory power. Therefore, it seems to be justified to include the criterion extranodal spread into TNM classification for squamous cell carcinomas of the oral cavity and the oropharynx. There is no significant relation between the dimensions of the primary tumor, such as the size and depth of invasion, and the metastatic extent in regional lymph nodes, i.e., intra- or extranodal spread." |
| **157** | **Sahoo 2019** | 150 | Retrospective Cohort study | India | a. >60, 110(73.33%) b.<60, 40(26.67%) | 99(66) | 1. GB sulcus, 113(75.33) 2. Tongue, 29(19.33) 3. Floor of mouth, 2(1.33) 4. Retromolar, 4(2.67) 5. Maxilla, 2(1.67) | All | Patients with biopsy-proven oral squamous cell carcinoma were done from the Department of Oral Pathology | 1. Lymphovascular invasion  2. Perineural invasion | "With the suggestion of a standardized reference point to measure DOI for the first time, this study has shown an association of TT1, TT2, PNI, and LVI with LNM in the Indian Population. The mathematical model can help in identifying high-risk cases in OSCC." |
| **158** | **Sakamoto 2016** | 252 | Retrospective Cohort study | Japan | a. ≧65, 155(73.11%)  b. ＜65, 97(45.75%) | 141(55.95) | Tongue and FOM | All | 1. Patients with OSCC  2. Underwent surgery of resection | Perineural invasion | "Metastasis to the lower neck and local recurrence were risk factors of DM in oral cancer patient." |
| **159** | **Sakata 2018** | 97 | Retrospective Cohort study | Japan | 64 ± 19 | 56(57.7) | OTSCC, 97(100) | All | 1. Patients with cT2N0 TSCC  2. Underwent transoral partial glossectomy alone as a primary treatment  3. Between January 2001 and September 2015, were enrolled in this study | 1. Depth of invasion  2. Growth pattern  3. Tumor budding  4. Perineural invasion | "We conclude that the evaluation of tumor budding is effective for identifying populations at high risk of occult neck metastasis, which will enable the planning of appropriate therapeutic strategies for patients with cT2N0 TSCC. Furthermore, cytokeratin staining is recommended over HE staining for simpler and more accurate evaluation of tumor budding." |
| **160** | **Salama 2021** | 293 | Retrospective Cohort study | USA | NR | NR | OTSCC, 293(100) | T1 and T2 | 1. Patients underwent primary resection  2. From 2000 to 2012  3. A diagnosis of OTSCC  4. The slides of primary resection were available for review 5. Pathologic staging of T1 or T2 | 1. Depth of invasion  2. Tumor stage  3. Lymphovascular invasion  4. Degree of differentiation  5. Perineural invasion | "We therefore propose to replace DOI, a complicated measurement with many challenges, with TT in the pT staging system." |
| **161** | **Sarioglu 2010** | 64 | Retrospective Cohort study | Turkey | 58.5 ± 14.44 | 60(93.75) | 1. Supraglottic, 22(34.38) 2. Glottic, 5(7.81) 3. Subglottic, 4(6.25) 4. Transglottic, 9(14.06) | 1. T2, 13(20.31) 2. T3, 33(51.56) 3. T4, 18(28.13) | 1. Patients with laryngeal carcinoma  2. All diagnosed and treated at Dokuz Eylul University Hospital  3. Had complete follow-up information  4. Patients were treated by partial or total laryngectomy and neck dissection,  followed by adjuvant radiotherapy | Tumor budding | "These results suggest that budding might be a valuable prognostic factor, particularly for distant metastasis in laryngeal carcinomas." |
| **162** | **Sawazaki-calone 2015** | 113 | Retrospective Cohort study | Finland | 31 to 84 | 95(84.1) | 1. Tongue  2. FOM  3. Palate 4. Retromolar region | T1, T2, T3 and T4 | 1. Patients with primary OSCC  2. Treatment based on radical surgery with or   without postoperative radiotherapy and/or chemotherapy, and availability of all paraffin-embedded blocks | 1. Tumor budding  2. Degree of differentiation | "A significant association between the BD model and outcome of OSCC patients was observed, indicating this new histopathological grading system as a possible prognostic tool." |
| **163** | **Seki 2016** | 209 | Retrospective Cohort study | Japan | 62.25 ± 19.33 | 150(71.77) | 1. Tongue, 107(51.2)  2. FOM, 15(7.18) 3. Gingiva, 72(34.45) 4. Buccal mucosa, 13(6.22)  5. Palate, 1(0.48) 6. Lip, 1(0.48) | All | 1. Cases of SCC in the Gunma University Hospital  2. Between 2009 and 2014 | 1. Depth of invasion  2. Tumor stage  3. Lymphovascular invasion  4. Degree of differentiation  5. Perineural invasion | "The assessment of tumor budding is effective in predicting prognosis in cN0 early stage OSCC. In T1/2 stage and cN0 cancer, prophylactic neck dissection to prevent LNM should be considered when the tumor budding score regarding preoperative biopsy specimens is intermediate or high." |
| **164** | **Sekikawa 2019** | 402 | Retrospective Cohort study | Japan | 63 ± 20.78 | 177(44) | 1. Tongue, 192(47.76)  2. FOM, 28(6.97) 3. Upper gingiva, 55(13.68)  4. Buccal mucosa, 22(5.47) 5. Others, 12(2.98) 6. Lower gingiva, 93(23.13) | 1. T1-T2, 236(58.7)  2. T3-T4, 166(41.3) | Patients with OSCC at a single institution | Degree of differentiation | "In the patients with DMs, stage cN0 and a late interval to DM diagnosis were associated with long-term survival. Pulmonary metastasectomy could be worth considering to improve survival." |
| **165** | **Shan 2020** | 101 | Retrospective Cohort study | China | 59.86 ± 12.14 | 47(46.5) | OTSCC, 101(100) | 1. T1, 31(30.69)  2. T2, 70(69.31) | 1. Histology-proven OTSCC through preoperative biopsy  2. Primary cT1N0 or cT2N0 OTSCC that had not been treated  3. No radiotherapy or chemotherapy in the clinical history  4. No clinical conditions that might affect the NLR  5. All patients treated with neck dissection | 1. Depth of invasion  2. Tumor stage  3. Lymphovascular invasion  4. Degree of differentiation  5. Perineural invasion | "ML shows a better performance in predicting lymph node metastasis of early-stage OTSCC than conventional prediction methods of DOI, NLR, or tumor budding." |
| **166** | **Sharma 2017** | 60 | Retrospective Cohort study | India | NR | NR | OTSCC, 60(100 | All | Cases of OSCC of known nodal status were selected for immunohistochemical staining of tumor | 1. Tumor budding  2. Degree of differentiation | "Expression successfully identifies the motility profile of a tumor, irrespective of the pattern of invasion. The presence of larger motile islands in the tumor cohort supports the concept of ‘Collective Cell Migration.’ Podoplanin also aids in the evasion of immune responses by inducing platelet aggregation over tumor cells, thereby favoring distant metastasis. A multivariate model using immunohistochemical staining with D2-40 renders greater sensitivity to the prediction of lymph node metastasis." |
| **167** | **Shen 2013** | 116 | Retrospective Cohort study | Taiwan | 53 ± 14.44 | 99(85.34) | Tongue and Oral cavity | 1. T1, 45(38.8) 2. T2, 45(38.8) 3. T3, 17(14.6) 4. T4, 9(7.8) | 1. OTSCC cases were reviewed  2. Histopathological features, including tumor differentiation, tumor thickness, section margin status, and cancer invasion front pattern, were checked and recorded | Perineural invasion | "Anti-S-100 immunostaining is an effective technique to detect occult PNI. Both the positive PNI and NGF expression level are valuable biomarkers that can predict the progression of OTSCC and prognosis of OTSCC patients." |
| **168** | **Shetty 2020** | 30 | Retrospective Cohort study | India | 49.95 ± 16.15 | 17(56.7) | OTSCC, 30(100) | All | 1. Cases with OSCC 2. During the period from 2012 to 2014, were considered for histopathological grading | 1. Tumor budding  2. Degree of differentiation | "The study found that the degree of keratinization, nuclear polymorphism, the pattern of invasion, and the host response showed significant variation at the invasive front of the tumor as compared to superficial parts, which makes Bryne’s grading system more reliable than Broder’s grading system." |
| **169** | **Shimizu 2018** | 91 | Retrospective Cohort study | Japan | 64.25 ± 15.87 | NR | OTSC, 91(100) | Early | 1. Diagnostic tissue blocks for patients with cT1,2N0M0 OSCC  2. Had undergone transoral tumor ablation surgery with or without elective neck dissection 3. Between January 2004 and December 2013 | 1. Tumor budding  2. Margin status  3. Lymphovascular invasion  4. Degree of differentiation  5. Perineural invasion | "Our results show that tumor budding with more than five buds and a grade 4C or 4D mode of invasion is observed more frequently in patients with cT1,2N0M0 OSCC with regional metastasis, though only a high intensity of tumor budding showed a significant correlation with regional metastasis in multivariate analysis." |
| **170** | **Shinn 2018** | 93 | Retrospective Cohort study | USA | 59 ± 5.77 | 57(61.3) | 1. Oral cavity  2. FOM  3. Tongue | 1. T1, 83(89.25)  2. T2, 10(10.65) | 1. Patients undergoing primary surgical resection of T1 or T2 oral tongue cancer 2. Slected against neck treatment between 2000 and 2015 | Depth of invasion | "Depth of invasion is significantly associated with nodal metastasis and has been added to the 8th AJCC staging guidelines. Variable depths of invasion have been associated with regional metastasis; however, there is likely not a critical depth that predicts neck recurrence due to progressive hazards and cumulative risk of occult metastasis. The risk of regional metastasis is likely much greater than previously believed and increases progressively with increasing depth." |
| **171** | **Simonetti 2017** | 50 | Retrospective Cohort study | Italy | 58 ± 15.59 | 26(52) | OTSCC, 50(100) | All | Patients with squamous cell carcinoma of the oral cavity who underwent surgery | Tumor Grade | "The emerging picture of the involvement of CEACAM1 is thus very complex. Nonetheless, the CEACAM1 expression pattern in OSCC has been confirmed, and our most important finding was that CEACAM1 seems to be a marker of carcinoma in situ. However, our study was limited to a small series. It is still difficult for pathologists to make objective differential diagnoses only on histological characteristics of hematoxylin–eosin-stained sections; therefore, an immunohistochemical marker could help pathologists to distinguish CIS from severe dysplasia more easily." |
| **172** | **Sindhura 2023** | 32 | Retrospective Cohort study | India | 22 to 80 | 29(91) | Oral tongue and Buccal mucosa | 1. T1, 5(16) 2. T2, 19(59) 3. T3, 5(16) 4. T4, 3(9 | 1. Excision biopsy was done for 59 cases  2. Of these, lymph node dissection was done in 32 cases  3. Lesions from the tongue and buccal mucosa were included in the study | 1. Tumor budding  2. Margin status  3. Lymphovascular invasion  4. Degree of differentiation  5. Perineural invasion | "The potential of morphological features, such as Tumour Budding (TB) evaluated in OSCC diagnostic preoperative biopsies may aid in identifying patients who may benefit from more aggressive treatments." |
| **173** | **Singh 2019** | 400 | Retrospective Cohort study | India | Mean(52.67) | 315(78.8) | OTSCC, 400(100) | T4, 400(100) | 1. Consecutive surgically treated pT4 OTSCC patients  2. Between January 2012 and December 2015 | 1. Margin status  2. Tumor stage | "The presence of lymphovascular emboli may impact the status of bony margins. Based on our results, to achieve an “adequate margin in bone,” we propose cutting the bony at least 15 mm away from the clinically discernible tumor when treating advanced oral cancers." |
| **174** | **Siriwardena 2018** | 465 | Prospective cohort study | Sri Lanka | Mean(57.8) | 329(70.75) | 1. Buccal mucosa  2. Floor of the mouth  3. Lower alveolar ridge  4. Palate  5. Tongue  6. Upper alveolar ridge | All | 1. All the patients with histologically confirmed OSCC  2. Had undergone surgical resection with neck dissection  3. From 1999 to 2012 | 1. Tumor budding  2. Margin status  3. Lymphovascular invasion  4. Degree of differentiation  5. Perineural invasion | "The proposed predictive model may provide some guidance for maxillofacial surgeons to decide the appropriate treatment plan for OSCC, especially in developing countries. This model appears to be reliable and simple and may guide surgeons in planning surgical management of neck nodes." |
| **175** | **Solomon 2021** | 187 | Prospective cohort study | Canada | Mean(61.7) | 115(61.5) | 1. Tongue, 90(48.13) 2. Floor of mouth, 34(18.18) 3. mandibular/maxillary   alveolus/gingiva, 23(12.3) 4. Others, 40(21.39) | All | 1. All adult patients aged 18 or older  2. Underwent primary surgical resection of OSCC  3. Patients treated between the dates of January 1, 2009 – December 31, 2013 | Marginal status | "While most conventional risk factors for OSCC conferred a worsened outcome, close margins did not. One explanation for this would be that close margins (< 5 mm) are equivalent to clear margins, and the cutoff definition for a close margin should be re-evaluated. Lack of standardized pathology could also reduce the accuracy of reporting of close surgical margins." |
| **176** | **Son 2017** | 211 | Retrospective Cohort study | South Korea | 54.75 ± 18.19 | 101(47.87) | 1. Tongue, 188 (89.2)  2. Oral cavity, 23 (10.8) | 1. T1, 59(37.6) 2. T2, 14(8.9) 3. T3, 19(12.1) 4. T4, 65(41.4) | 1. Patients who underwent definitive surgery for OSCC  2. Between 1994 and 2006 | Depth of invasion | "LN ratio is an independent and predictive determinant of post-treatment recurrence and survival." |
| **177** | **Sparano 2004** | 45 | Retrospective Cohort study | USA | NR | 28(62.22) | OTSCC, 45(100) | Early | Patients with early oral tongue cancer and a clinically negative neck (T1/T2N0) | 1. Tumor budding  2. Margin status  3. Lymphovascular invasion  4. Degree of differentiation  5. Perineural invasion | "The clinical and histopathologic factors studied herein permit greater selectivity and more informed decision-making than does the presurgical evaluation when addressing elective neck treatment for early N0 oral tongue cancer. The multivariate model derived from this study appears to be a more reliable method for determining the patients most likely to benefit from elective neck dissection." |
| **178** | **Spoerl 2021** | 717 | Retrospective Cohort study | Germany | 60.15 ± 18.18 | 515(71.8) | 1. Buccal mucosa, 51(7.1) 2. Upper alveolus and gingiva, 22(3.1) 3. Lower alveolus and gingiva , 106(14.8) 4. Hard palate, 48(6.7) 5. Tongue, 210(29.3) 6. Floor of mouth, 280(39.1) | 1. T1, 290(40.4) 2. T2, 236(32.9) 3. T3, 56(7.8) 4. T4, 135(18.8) | Patients with primarily resected OSCC | 1. Tumor size  2. Lymph node ratio | "LNR was shown to be an independent prognostic factor for the outcome of OSCC in a population-based cohort in uni as well as multivariate analysis. Hereby, an LNR ≥ 0.055 predicted a shorter OAS and RFS in our cohort." |
| **179** | **Spoerl 2022** | 445 | Retrospective Cohort study | Germany | NR | 312(70.6) | 1. Buccal mucosa, 33(7.42) 2. Upper alveolus and gingiva, 19(4.27)  3. Lower alveolus and gingiva, 71(15.96) 4. Hard palate, 32(7.19)  5. Tongue,132(29.66) 6. Floor of mouth. 158(34.83) | 1. T1, 183(41.1) 2. T2, 141(31.7)  3. T3, 37(8.3)  4. T4, 84(18.9) | 1. Patients with newly diagnosed OSCC surgical treatment  2. Between January 2010 and December 2017 | Perineural invasion | "PNI appears to be a relevant prognostic factor for the survival and recurrence of OSCC patients having received tumor resection to negative margins combined with a cervical lymphadenectomy. When focusing on recurrence in detail, the development of distant metastasis was significantly increased in Pn1 patients. Currently, an indication of adjuvant treatment for verified PNI in OSCC patients is under debate. So far, the present multicenter cohort study could not derive reliable implications of a potential outcome benefit after adjuvant RT or RCT. Additional in vivo, as well as in vitro, studies are necessary to unveil the molecular basis of PNI in OSCC and to develop targeted therapeutical approaches to address this special tropism of tumor spread. Based on our findings, we highly recommend emphasizing PNI in the TNM staging concept." |
| **180** | **Stoop 2020** | 210 | Retrospective Cohort study | Netherlands | 65.99 ± 11.39 | 127(60.4) | OTSCC, 210(100) | All | 1. Patients who had undergone marginal or segmental resection  2. Between January 2000 and December 2017 for the first primary OSCC  3. With mandibular invasion confirmed by histopathological examination of the resection specimen | Marginal status | "There was no difference in outcome between the two types of mandibulectomy." |
| **181** | **Sundaram 2023** | 122 | Retrospective Cohort study | India | 50.5 ± 11.77 | 85(69.7) | OTSCC, 122(100) | 1. T1, 15(12.3) 2. T2, 44(36.1) 3. T3, 63(51.6) | 1. Patients with OTSCC  2. Managed primarily by surgery with curative intent  3. From January 2014 to December 2016 | 1. Perineural invasion  2. Lymph node ratio | "The LNR could be an important prognosis factor for OTSCC that helps in determining better clinical outcomes." |
| **182** | **Suresh 2015** | 105 | Retrospective Cohort study | India | 49.2 ± 12.98 | 15(14.28) |  | 1. T1, 6(5.7) 2. T2, 22(21) 3. T3, 31(29.5) 4. T4, 46(43.8) | Patients of OSCC | 1. Tumor budding  2. Margin status  3. Lymphovascular invasion  4. Degree of differentiation  5. Perineural invasion | "Significant association of cervical LN metastasis with high grade of differentiation, lack of E-cadherin expression, high Ki-67, and cyclin D1 expression was seen." |
| **183** | **Tai 2013** | 212 | Retrospective Cohort study | Taiwan | 53.35 ± 18.19 | 187(88.2) | Oral Tongue and Buccal mucosa | 1. T1, 106(50) 2. T2, 106(50) | 1. Patients with OSCC 2. Follow-up period up to 24 months | 1. Tumor budding  2. Margin status  3. Lymphovascular invasion  4. Degree of differentiation  5. Perineural invasion | "PNI can be a major determinant for higher LN metastasis and poor 5-year DSS rates associated with increased tumor thickness in T1–2 oral tongue and buccal SCC. Careful evaluation of PNI should be mandatory in routine pathologic examination, aside from the measurement of tumor thickness." |
| **184** | **Tam 2018** | 212 | Retrospective Cohort study | USA | 55.25 ± 20.49 | 92(43) | OTSCC, 212(100) | 1. T1, 81(38) 2. T2, 131(62) | 1. Patients with lateral OTSCC  2. Treated at the MD Anderson Cancer Center  3. Between March 1997 and March 2012 | Depth of invasion | "The optimal DOI cut-point for detection of occult nodal metastasis was 7.25 and 8 mm for OS and DSS at five years. DOI is an independent predictor of OS and DSS." |
| **185** | **Tan 2023** | 73 | Retrospective Cohort study | Turkey | 59.06 ± 16.45 | 37(50.7) | 1. Tongue, 42(57.5)  2. Buccal mucosa, 26(35.6)  3. Floor of mouth, 4(5.5) 4. Palate, 1(1.4) | 1. T1, 22(30.1) 2. T2, 34(46.6) 3. T3, 11(15.1) 4. T4, 6(8.2) | Patients with oral carcinoma | 1. Tumor–stroma ratio  2. Tumor budding | "We concluded that tumor budding and single-cell invasion should be considered prognostic histopathologic parameters in OSCC." |
| **186** | **Tarsitano 2016** | 67 | Retrospective Cohort study | Italy | NR | 45(67.16) | Tongue and Floor of the mouth | 1. T1, 31(46.27) 2. T2, 36(53.73) | 1. Previously untreated patients  2. Underwent primary surgical extirpation with elective neck dissection 3. Owing to the retrospective nature of this study | Tumor stage | "Infiltration depth was identified as an important predictor for neck nodal status. In this specific population, mortality was associated with increasing tumor infiltration depth." |
| **187** | **Thiagarajan 2014** | 586 | Retrospective Cohort study | India | Mean(48) | 416(70.98) | OTSCC, 586(100) | All | 1. Patients with pathologically proven OTSCC-operated  2. Between January 2007 and June 2010, treatment-naive surgery was the first‐line treatment | Tumor stage | "Other than nodal metastasis, tumor‐related factors like thickness and perineural invasion are adverse prognostic factors and can influence survival. These patients, especially in case of early-stage cancers, may potentially benefit from postoperative adjuvant radiotherapy." |
| **188** | **Thompson 1986** | 21 | Retrospective Cohort study | South Africa | 61.51 ± 12.72 | NR | Oral tongue and Oral cavity | All | Patients with oral carcinoma | Depth of invasion | "Carcinomas affecting other oral sites exhibited confirmed nodal metastasis without infiltration beyond the deep lamina propria when evaluated as a single group. Tongue carcinomas showed an almost equal distribution between lesions involving the lamina propria and lesions infiltrating the skeletal muscle. Infiltrative invasion of tongue carcinomas into the underlying skeletal muscle does not appear to be more prognostically significant in comparison to invasion confined to the deep lamina propria. It would appear that the prognostic value for any single site for oral carcinoma in relation to the depth of invasion is significant only when the primary lesion is confined to the superficial half of the lamina propria (LI). " |
| **189** | **Ting 2021** | 98 | Retrospective Cohort study | Taiwan | 57.75 ± 17.04 | 93(94.89) | 1. Buccal, 34(34)  2. Tongue, 29(29.6)  3. Gum, 19(19.4)  4. Hard palate, 8(8.2)  5. Others, 8(8.2) | 1. T3, 28(28.57) 2. T4, 70(71.43) | 1. Patients with T3–4 OSCC  2. Underwent curative surgery  3. Between Jan 2002 and Dec 2010 | 1. Lymphovascular invasion  2. Perineural invasion | "combined consideration of PNI and LVI is important in prognostication and potential clinical application of T3–4 OSCC. PNI/LVI double positive independently predicts LN+, DM, and poor DSS in T3–4 OSCC and can be used as a good marker to select DM high-risk patients for future novel adjuvant therapy trials with the goal of reducing DM and improving the survival for T3–4 OSCC." |
| **190** | **Toom 2019** | 199 | Prospective cohort study | Netherlands | 60 ± 17.31 | 100(50.1) | 1. Tongue, 121(61)  2. Floor of mouth, 53(27)  3. Buccal mucosa, 16(8)  4. Inferior alveolar process, 5(3) 5. Other, 4(2) | 1. T1, 132(66)  2. T2, 67(34) | OSCC patients, DOI measurements, and SLNB were performed | 1. Depth of invasion  2. Tumor stage | "DOI seems to be a poor predictor for regional metastasis in patients with cT1-2N0 OSCC. Therefore, staging of the neck using SLNB in patients with early-stage oral cancer should also be performed in tumors with limited DOI and probably in T3 (8th TNM) OSCC ≤4 cm diameter." |
| **191** | **Van Lanschot 2020** | 300 | Retrospective Cohort study | Netherlands | 62.75 ± 20.20 | 158(52.7) | 1. Buccal mucosa 2. Upper alveolus and gingiva 3. Lower alveolus and gingiva  4. Hard palate  5. Tongue  6. Floor of mouth 7. Lips | Early | 1. All patients with primary OCSCC (pT1 or pT2)  2. Surgically treated between January 2013 and May 2018 | Depth of invasion | "A DOI of ≥ 4 mm is an accurate cut-off value for performing an END in early-stage OCSCC. END results in higher survival rates and lower regional recurrence rates in patients with DOI ≥ 4 mm." |
| **192** | **Varsha 2015** | 117 | Retrospective Cohort study | India | NR | NR | OTSCC, 117(100) | 1. T1, 9(7.7) 2. T2, 23(19.8) 3. T3, 13(11.2) 4. T4, 71(61.3) | 1. Patients with primary and recurrent cases of OSCC  2. PNI was correlated with tumor thickness, lymph node status, and the different histologic grades | Perineural invasion | "Our study showed that the incidence of PNI was as high as 40% in OSCC. PNI was present in both primary and recurrent tumors, irrespective of its histologic grading. Tumor thickness and lymph node status correlated well with PNI. Therefore, the presence of PNI should be checked in every surgical specimen with OSCC, as it gives significant predictive value and influences treatment decisions, recurrence, and distant metastasis. The presence of PNI necessitates more aggressive resection, coincident management of neck lymph nodes, and the addition of adjuvant therapy. Also, targeted drug therapy for this type of tumor spread can open up new avenues in the treatment of OSCC." |
| **193** | **Verma 2021** | 78 | Retrospective Cohort study | India | 48 ± 12.9 | 70(89.9) | 1. Alveolar Process  2. Buccal Mucosa  3. Gingivo-buccal sulcus  4. Lips  5. Maxilla  6. Palate  7. Paranasal sinus  8. Retromolar | Early | Early-stage OSCC patients | 1. Tumor budding  2. Margin status  3. Lymphovascular invasion  4. Degree of differentiation  5. Perineural invasion | "The prognostic information supplied by evaluating DOI, LVI, and WPOI warrants the inclusion of these parameters in the standard reporting format for all cases of OSCC." |
| **194** | **Vidiri 2019** | 43 | Retrospective Cohort study | Italy | 60.75 ± 14.71 | 18(41.9) | OTSCC, 43(100) | 1. T1, 10(23.26)  2. T2, 12(27.91)  3. T3, 21(48.84) | 1. OTSCC patients  2. Underwent curative surgery  3. From 2013 to 2018  4. Preoperative MRI study performed within 3–4 weeks before surgery  5. The presence of a measurable tumor on MRI | 1. Depth of invasion  2. Tumor stage | "MRI-derived depth of invasion should be measured in the pretreatment assessment of oral tongue squamous cell carcinoma as it has an excellent inter-reader reliability and nearly excellent radiological–pathological correlation." |
| **195** | **Wang 2016** | 345 | Retrospective Cohort study | Taiwan | 49.43 ± 10.68 | 325(94.2) | 1. Buccal mucosa 2. Upper alveolus and gingiva 3. Lower alveolus and gingiva  4. Hard palate  5. Tongue  6. Floor of mouth 7. Lips | Late | Patients with oral squamous cell carcinoma | Tumor thickness | "We conclude that genetic information from NGS may improve the prognostic stratification offered by traditional prognosticators in resected OSCC patients with ECE. Our findings will contribute to the implementation of precision medicine in OSCC patients." |
| **196** | **Wang 2021** | 112 | Retrospective Cohort study | Italy | 57.89 ± 11.42 | 75(66.96) | 1. Tongue  2. Lower gingiva  3. Upper gingiva  4. Buccal mucosa  5. Floor of the mouth  6. Hard palate | 1. T1, 19(17)  2. T2, 79(70.5) 3. T3, 14(12.5) | 1. A pathological diagnosis of SCC  2. A tumor located in the tongue, lower gingiva, upper gingiva, buccal mucosa, floor of the mouth, or hard palate  3. A primary tumor without evidence of distant metastasis  4. Underwent radical resection of the primary tumor with or without neck dissection 5. No previous treatment, such as neoadjuvant chemotherapy or prior radiotherapy 6. Had undergone complete follow-up | 1. Depth of invasion  2. Tumor stage | "High expression of CDKN2A and PLAU was associated with lymph node metastasis in OSCC. The prediction model, including CDKN2A, PLAU, T stage, and pathological grade, can be used as the best diagnostic model for lymph node metastasis in OSCC." |
| **197** | **Wang 2016 (2)** | 112 | Retrospective Cohort study | Australia | 66 ± 14.9 | 66(59) | OTSCC, 112(100) | 1. T1, 48(44.9) 2. T2, 42(39.3) 3. T3, 15(14.0) 4. T4, 2(1.7) | Patients by the Northern Sydney Cancer Centre (Australia) with primary oral tongue SCC were conducted. | 1. Tumor thickness  2. Tumor stage | "Despite some previous evidence for a 4-mm tumor thickness cut-off in oral tongue SCCs, thinner tumors (2–3.9 mm) can also have a propensity for cervical node metastasis. Patients in this category require close monitoring for regional recurrence if they do not have a neck dissection." |
| **198** | **Warburton 2007** | 29 | Retrospective Cohort study | USA | 63.25 ± 13.57 | 18(62.08) | Oral Tongue and Floor of the mouth | Early | 1. Patients diagnosed with early-stage (T1N0M0 or T2N0M0) OTSCC  2. Treated at the University of Maryland between 1994 and 2000  3. Only patients treated by primary tumor resection without neck dissection or neck irradiation were included in the study | 1. Tumor stage  2. Pattern of invasion  3. Lymphoplasmacytic infiltration | "Analysis of these parameters may help to identify patients who would benefit from a neck dissection or irradiation by predicting the likelihood of lymph node metastasis." |
| **199** | **Wedemeyer 2013** | 63 | Retrospective Cohort study | Germany | 56.9 ± 9.8 | 48(76.2) | 1. Floor of mouth, 34(54) 2. Tongue, 12(19) 3. Alveolar gingiva of the upper and lower jaw, 13(20.6) 4. Hard palate, 3(4.8) 5. Cheek, 1(1.6) | All | 1. Patients with primary OSCC of stages II-IV  2. Treated with a concomitant neoadjuvant radiochemotherapy followed by radical surgery | Marginal status | "Tumour regression grading, nodal stage and status of resection margins predict prognosis in patients after neoadjuvant treatment. Currently, there are no pretreatment clinicopathological parameters that predict good tumor response to therapy. Thus, identifying non-responding patients who might benefit from intensified systemic therapy requires surgical resection and consecutive histopathological assessment. Therefore, further investigation and validation of new, especially molecular predictors of tumor response to radiochemotherapy remain an unmet, future clinical need." |
| **200** | **Wei 2018** | 314 | Retrospective Cohort study | Taiwan | 53.75 ± 18.18 | 275(87.6) | 1. OTSCC, 193(61.5)  2. Buccal SCC, 69(22) | 1. T1, 145(46.18)  2. T2, 169(53.82) | 1. Patients with T1-T2 OSCC  2. Underwent curative surgery  3. From June 2001 to August 2009 | Perineural invasion | "PNI focus number can be a novel parameter for PNI quantification in early OSCC. Although optimal quantification methods still require further investigation, this study offers clear clinical support for the nerve-tumor interaction hypothesis and advocates further mechanistic research for the exploration of PNI-related treatment concepts for OSCC." |
| **201** | **Weimar 2018** | 335 | Retrospective Cohort study | Canada | 60.5 ± 21.36 | 206(61) | Oral tongue and other subsites | 1. T1, 119(36) 2. T2, 161(48)  3. T3, 55(16) | Patients with all OSCC subsites except the lip (typically different etiology [i.e., sun exposure] rather than smoking/drinking) | Tumor thickness | "This study demonstrates a good radiologic-pathologic tumor thickness correlation. Interrater and interrater reliability for radiologic tumor thickness were excellent. The radiologically thicker tumor was predictive of inferior survival." |
| **202** | **Woolgar 2003** | 173 | Retrospective Cohort study | UK | 62 ± 11.6 | 124(72) | 1. Floor of mouth, 48(28)  2. Oral tongue, 49(28) 3. Orophar ynx, 30(17)  4. RMT, 21(12)  5. Buccal mucosa, 10(6)  6. Alveolar ridge/gum, 15(9) | All | 1. Patients undergoing surgery as the primary treatment for OSCC  2. Between October 1989 and December 1999 | Tumor stage | "The Cox model showed the most predictive factor was extracapsular spread followed by the status of resection margins. The extracapsular spread should be incorporated into pathological staging systems. Even microscopic extracapsular spread is of critical importance and must be sought, especially in small-volume metastatic disease." |
| **203** | **Wu 2019** | 141 | Retrospective Cohort study | China | Mean(55) | 75(53.19) | OTSCC, 141(100) | 1. T1, 60(42.56)  2. T2, 81(57.44) | TSCC patients staged as T1–2N0 | 1. Tumor budding  2. Margin status  3. Lymphovascular invasion  4. Degree of differentiation  5. Perineural invasion | "Trabes growth pattern of the invasive front was a potent risk factor for TSCC cervical lymph node relapse and indicated poor prognosis. Preventive therapy, including selective neck dissection, was thus suggested for certain patients." |
| **204** | **Xie 2014** | 195 | Retrospective Cohort study | China | 52± 17.90 | 95(48.71) | OTSCC, 145(100) | Early | 1. Patients with TSCC in the T1/2 stage  2. They were all in clinical T1/2N0M0 stage and confirmed pathologically with T1 or T2 stage lesions | 1. Depth of invasion  2. Tumor budding | "Tumor budding is a frequent event in tongue squamous cell carcinoma. It independently predicted prognosis of patients with T1/2 stage tongue squamous cell carcinoma and may be used for routing pathological diagnosis and the decision of elective lymph node dissection." |
| **205** | **Xie 2019** | 255 | Retrospective Cohort study | China | 51.13 ± 19-83 | 130(50.98) | OTSCC, 255(100) | All | 1. Patients with OTSCC  2. Patients underwent resection of the primary tumor and/ or neck dissection  3. Between January 2001 and December 2015 | 1. Depth of invasion  2. Tumor budding | "International Tumor Budding Consensus Conference scoring system is a simple, reliable, and reproducible method to measure tumor budding in OTSCC, which should be included in the routine pathological report." |
| **206** | **Xu 2021** | 329 | Retrospective Cohort study | USA | NR | NR | OTSCC,329(100) | Early | 1. Patients who underwent primary resection  2. From 2000 to 2012  3. A diagnosis of OTSCC with a tumor greatest dimension of 4 cm or less  4. The slides of primary resection were available for review | 1. Tumor budding  2. Margin status  3. Lymphovascular invasion  4. Degree of differentiation  5. Perineural invasion | "We compared the performance of three related histologic parameters, the multifactorial descriptive histologic grade, the qualitative WPOI, and the semi-quantitative tumor budding, and found that WPOI and tumor budding were superior to histologic grade in OTSCC. While WPOI 5 was an independent adverse prognostic factor for OS, high tumor budding was associated with a high risk of nodal metastasis on multivariable logistic regression analysis. Therefore, we propose to include tumor budding and WPOI in the routine pathologic reports of patients with OTSCC." |
| **207** | **Yamada 2017** | 216 | Retrospective Cohort study | Germany | 64.7 ± 19.62 | 131(60.6) | 1. Tongue, 111(51.4)  2. Lower gingiva, 32(14.8)  3. Upper gingiva, 26(12)  4. Oral floor, 23(10.6) | 1. T1, 40(18.5) 2. T2, 91(42.1) 3. T3, 23(10.6) 4. T4, 62(28.7) | Patients with ECS who underwent initial surgery without neoadjuvant therapy were included in the study population. | Tumor budding | "These results suggest that the progression level of ECS and tumor budding are useful prognostic factors in OSCC patients." |
| **208** | **Yamagata 2019** | 95 | Retrospective Cohort study | Japan | 63.5 ± 15.3 | 52(54.73) | 1. Tongue  2. Lower gingiva  3. Floor of mouth  4. Buccal mucosa  5. Upper gingiva  6. Hard palate | All | Patients who were diagnosed with OSCC and treated by neck dissection | 1. Perineural invasion  2. Tumor Grade | "As a predictive factor, high LNR (≥0.04) was associated with diminished survival, and intensive adjuvant therapy may improve the prognosis for patients with high LNR." |
| **209** | **Yamakawa 2018** | 337 | Retrospective Cohort study | Japan | 63.5 ± 15.30 | 192(56.97) | OTSCC, 337(100) | Early | 1. Patients with Stage I‐II TSCC  2. Underwent surgical resection of the primary tumor without elective neck dissection (END)  3. Between January 2008 and December 2014  4. All the patients were at least 15 years of age | 1. Tumor budding  2. Depth of invasion  3. Tumor stage | "In addition to conventional predictors, high-grade tumor budding and adjacent tissue at the invasive front can serve as useful predictors of DNM in early tongue cancer." |
| **210** | **Yamamoto 2014** | 193 | Retrospective Cohort study | Japan | 56.5 ± 20.49 | 57(34) | OTSCC, 193(100) | All | 1. Patients with OTSCC  2. Treated at a hospital between 2000 and 2010 | Depth of invasion | "The results suggest that primary tumor depth may be used as a prognostic factor in tongue squamous cell carcinoma." |
| **211** | **Yang 2021** | 541 | Retrospective Cohort study | South Korea | NR | 235(65.5) | 1. Buccal cheek, 36(10) 2. Floor of mouth, 25(7) 3. Lip, 2(0.6) 4. Mandible, 120(33.4) 5. Maxilla, 45(12.5) 6. RMT, 36(10) 7. Tongue, 95(26.5) | Early | 1. Patients who had undergone surgical treatment for OSCC  2. From January 2000 to December 2020 | 1. Tumor budding  2. Margin status  3. Lymphovascular invasion  4. Degree of differentiation  5. Perineural invasion | "Based on multivariate analysis, we built a model to predict level IIb lymph node metastasis. The equation was as follows: logit[P]=-4.708+2.026×LVP+2.656×Level IIa metastasis." |
| **212** | **Yang 2018** | 34 | Prospective cohort study | China | NR | NR | OTSCC, 34(100) | T1 and T2 | Patients with early-stage oral tongue squamous cell carcinoma | Perineural invasion | "This study demonstrated that PNI was an invaluable pathological parameter to independently predict cervical metastasis, local relapse, neck relapse, and poor survival outcomes, but END could not improve benefits compared to observation for the PNI-positive patients." |
| **213** | **Yoshida 2018** | 78 | Retrospective Cohort study | Japan | 68.1± 14.71 | 29(37.2) | 1. Upper gingiva, 32(41) 2. Lower gingiva, 46(59) | 1. T1, 10(12.8) 2. T2, 43(55.1) 3. T3, 2(2.6) 4. T4a, 21(26.9) 5. T4b, 2(2.6) | 1. Patients diagnosed and treated for upper or lower gingival SCC  2. From January 2005 through December 2015 | Bone invasion | "Pathologically-proven medullary bone invasion and lymphovascular invasion were found to be key prognostic factors in gingival SCC. The results suggest that it is necessary to consider adjuvant therapy in patients with medullary bone invasion." |
| **214** | **Yoshizawa 2022** | 78 | Retrospective Cohort study | Japan | 66.4 ± 13.8 | 47(60.26) | OTSCC, 78(100) | 1. T1, 36(46.15)  2. T2, 25(32.05) 3. T3, 6(7.69) 4. T4, 11(14.1) | 1. Patients with OSCC  2. The clinicopathological features were characterized from January 2013 to January 2020 | Depth of invasion | "Loss of intercellular bridges in the DOI measurement area is a negative prognostic factor for OSCC and may be useful in selecting treatment." |
| **215** | **Yu 2019** | 246 | Retrospective Cohort study | USA | 52.75 ± 19.34 | 136(55.3) | OTSCC, 246(100) | 1. T1-T2, 166(67.5) 2. T3-T4, 80(32.5) | Patients with TSCC | 1. Tumor budding  2. Pattern of invasion | "IBD scoring model is strongly associated with lymph node metastasis and recurrence in TSCC and could be a promising survival predictor for TSCC patients. " |
| **216** | **Zenga 2019** | 4771 | Retrospective Cohort study | USA | a. <40, 358(8)  b. ≥40, 4413(92) | 2716(57) | 1. OTSCC, 3477(73)  2. Others, 1294(27) | 1. T1, 3219(68) 2. T2, 1552(33) | 1. Patients with early-stage clinically node-negative OCSCC  2. Underwent upfront surgery  3. Between 2004 and 2015 | Depth of invasion | "END with 18 or more nodes is associated with improved survival outcomes in patients with early-stage OCSCC regardless of DOI. END with less than 18 nodes, however, does not appear significantly different than observation of the neck alone. Achieving a lymph node yield of 18 or more is multifactorial and includes both patient and provider factors." |
| **217** | **Zhang 2019** | 80 | Retrospective Cohort study | China | a. ≥48, 45(56.25%)  b. <48, 35(43.75%) | 62(77.5) | OSCC, 80(100) | 1. T1-2, 42(52.5)   2. T3-4, 38(47.5) | 1. Patients from 35 to 55 years of age  2 Pathologically diagnosed with OSCC  3. From October 2015 to May 2018 | Tumor budding | "We found enhanced expression of IL-17 in the tumor and tumor margins, which was positively associated with tumor budding in the TIF. In addition, IL-17 combined with tumor budding was an independent predictor of prognosis for patients with OSCC." |

**References**

1. A Zaidan H. <http://www.omicsonline.org/open-access/natural-history-of-ovarian-cancer-1948-5956.1000278.php?aid=27726>. J Cancer Sci Ther. 2014;06(07).

2. Aaboubout Y, Van Der Toom QM, De Ridder MAJ, De Herdt MJ, Van Der Steen B, Van Lanschot CGF, et al. Is the Depth of Invasion a Marker for Elective Neck Dissection in Early Oral Squamous Cell Carcinoma? Front Oncol. 2021;11:628320.

3. Acharya S, Padmini A, Shetty S. Cervical lymph node metastasis in oral squamous cell carcinoma: A correlative study between histopathological malignancy grading and lymph node metastasis. Indian J Dent Res. 2013;24(5):599.

4. Acharya S, Raj M, Hallikeri K, Desai A. Histological assessment of budding and depth of invasion (BD) model in biopsies of oral squamous cell carcinoma. J Oral Maxillofac Pathol. 2020;24(3):581.

5. Adel M, Kao H-K, Hsu C-L, Huang J-J, Lee L-Y, Huang Y, et al. Evaluation of Lymphatic and Vascular Invasion in Relation to Clinicopathological Factors and Treatment Outcome in Oral Cavity Squamous Cell Carcinoma. Medicine. 2015;94(43):e1510.

6. Adel M, Tsao C-K, Wei F-C, Chien H-T, Lai C-H, Liao C-T, et al. Preoperative SCC Antigen, CRP Serum Levels, and Lymph Node Density in Oral Squamous Cell Carcinoma. Medicine. 2016;95(14):e3149.

7. Aires Felipe T, Lin Chin S, Matos Leandro L, Kulcsar Marco Aurélio V, Cernea Claudio R. Risk Factors for Distant Metastasis in Patients with Oral Cavity Squamous Cell Carcinoma Undergoing Surgical Treatment. ORL. 2017;79(6):347-55.

8. Aivazian K, Ebrahimi A, Low TH, Gao K, Clifford A, Shannon K, et al. Perineural invasion in oral squamous cell carcinoma: Quantitative subcategorisation of perineural invasion and prognostication. Journal of Surgical Oncology. 2015;111(3):352-8.

9. Akhter M, Hossain S, Rahman Q, Molla M. A study on histological grading of oral squamous cell carcinoma and its co-relationship with regional metastasis. J Oral Maxillofac Pathol. 2011;15(2):168.

10. Angadi PV, Patil PV, Hallikeri K, Mallapur MD, Hallikerimath S, Kale AD. Tumor Budding Is an Independent Prognostic Factor for Prediction of Lymph Node Metastasis in Oral Squamous Cell Carcinoma. Int J Surg Pathol. 2015;23(2):102-10.

11. Arora A, Husain N, Bansal A, Neyaz A, Jaiswal R, Jain K, et al. Development of a New Outcome Prediction Model in Early-stage Squamous Cell Carcinoma of the Oral Cavity Based on Histopathologic Parameters With Multivariate Analysis: The Aditi-Nuzhat Lymph-node Prediction Score (ANLPS) System. American Journal of Surgical Pathology. 2017;41(7):950-60.

12. Arun P, Arun I, Jain P, Manikantan K, Sharan R. Determinants of prognosis in patients with oral squamous cell carcinoma metastasizing to a single cervical lymph node. Oral Oncology. 2021;123:105586.

13. Asakage T, Yokose T, Mukai K, Tsugane S, Tsubono Y, Asai M, et al. Tumor thickness predicts cervical metastasis in patients with stage I/II carcinoma of the tongue. Cancer. 1998;82(8):1443-8.

14. Bachar G, Goldstein DP, Barker E, Lea J, O'Sullivan B, Brown DH, et al. Squamous cell carcinoma of the buccal mucosa: Outcomes of Treatment in the modern era. The Laryngoscope. 2012;122(7):1552-7.

15. Bae MR, Roh J-L, Kim JS, Choi S-H, Nam SY, Kim SY. Prediction of cervical metastasis and survival in cN0 oral cavity cancer using tumour 18F-FDG PET/CT functional parameters. J Cancer Res Clin Oncol. 2020;146(12):3341-8.

16. Balasubramanian D, Ebrahimi A, Gupta R, Gao K, Elliott M, Palme CE, et al. Tumour thickness as a predictor of nodal metastases in oral cancer: Comparison between tongue and floor of mouth subsites. Oral Oncology. 2014;50(12):1165-8.

17. Balla H, Uppala D, Majumdar S, Kotina S, Kodati S, Namana M. Evaluation of immunomorphological patterns of cervical lymph nodes in oral squamous cell carcinoma. J Oral Maxillofac Pathol. 2020;24(2):285.

18. Beggan C, Fives C, O'Leary G, Sheahan P, Heffron CC, Feeley L. Pattern of invasion and lymphovascular invasion in squamous cell carcinoma of the floor of the mouth: an interobserver variability study. Histopathology. 2016;69(6):914-20.

19. Bera RN, Singh AK, Tripathi R, Sharma NK. Influence of Site, Size, Depth of Invasion and Histologic Grading on the Occurrence of Cervical Level IIb Metastasis and Extranodal Extension in Clinically N0 Neck of Patients with OSCC: A Single Center Retrospective Analysis. J Maxillofac Oral Surg. 2022;21(4):1078-87.

20. Berger M, Grau E, Saure D, Ristow O, Thiele O, Hofele C, et al. Occurrence of cervical lymph node metastasis of maxillary squamous cell carcinoma – A monocentric study of 171 patients. Journal of Cranio-Maxillofacial Surgery. 2015;43(10):2195-9.

21. Bhatlawande HC, Kale AD, Desai KM, Hallikerimath S, Belaldavar C, Mane D, et al. Role of immunoreactive patterns of lymph nodes in neck dissection cases of oral squamous cell carcinoma: a clinical and histopathological study. J Korean Assoc Oral Maxillofac Surg. 2019;45(5):267.

22. Bhatta A, Rath R, Das SN, Mishra SS, Sethi D. Interaction between peritumoral reactivity and histomorphological pattern of lymph node reactivity in oral squamous cell carcinoma: A host immune status indicator. Journal of Cancer Research and Therapeutics. 2022;18(1):124-32.

23. Bjerkli I-H, Laurvik H, Nginamau ES, Søland TM, Costea D, Hov H, et al. Tumor budding score predicts lymph node status in oral tongue squamous cell carcinoma and should be included in the pathology report. PLoS ONE. 2020;15(9):e0239783.

24. Boxberg M, Jesinghaus M, Dorfner C, Mogler C, Drecoll E, Warth A, et al. Tumour budding activity and cell nest size determine patient outcome in oral squamous cell carcinoma: proposal for an adjusted grading system. Histopathology. 2017;70(7):1125-37.

25. Brockhoff HC, Kim RY, Braun TM, Skouteris C, Helman JI, Ward BB. Correlating the depth of invasion at specific anatomic locations with the risk for regional metastatic disease to lymph nodes in the neck for oral squamous cell carcinoma. Head & Neck. 2017;39(5):974-9.

26. Brown JS, Lowe D, Kalavrezos N, D'Souza J, Magennis P, Woolgar J. Patterns of invasion and routes of tumor entry into the mandible by oral squamous cell carcinoma. Head & Neck. 2002;24(4):370-83.

27. Caponio VCA, Troiano G, Togni L, Zhurakivska K, Santarelli A, Laino L, et al. Pattern and localization of perineural invasion predict poor survival in oral tongue carcinoma. Oral Diseases. 2023;29(2):411-22.

28. Chandavarkar V, Uma K, Sangeetha R, Mishra M. Immunomorphological patterns of cervical lymph nodes in oral squamous cell carcinoma. J Oral Maxillofac Pathol. 2014;18(3):349.

29. Chandler K, Vance C, Budnick S, Muller S. Muscle Invasion in Oral Tongue Squamous Cell Carcinoma as a Predictor of Nodal Status and Local Recurrence: Just as Effective as Depth of Invasion? Head and Neck Pathol. 2011;5(4):359-63.

30. Chang W-C, Chang C-F, Li Y-H, Yang C-Y, Su R-Y, Lin C-K, et al. A histopathological evaluation and potential prognostic implications of oral squamous cell carcinoma with adverse features. Oral Oncology. 2019;95:65-73.

31. Chang W-C, Lin C-S, Yang C-Y, Lin C-K, Chen Y-W. Lymph node density as a prognostic predictor in patients with betel nut-related oral squamous cell carcinoma. Clin Oral Invest. 2018;22(3):1513-21.

32. Chang YC, Nieh S, Chen SF, Jao SW, Lin YL, Fu E. Invasive pattern grading score designed as an independent prognostic indicator in oral squamous cell carcinoma. Histopathology. 2010;57(2):295-303.

33. Chatterjee D, Bansal V, Malik V, Bhagat R, Punia RS, Handa U, et al. Tumor Budding and Worse Pattern of Invasion Can Predict Nodal Metastasis in Oral Cancers and Associated With Poor Survival in Early-Stage Tumors. Ear Nose Throat J. 2019;98(7):E112-E9.

34. Chaudhary N, Verma R, Agarwal U, Gupta S, Jaitly S. Incidence of occult metastasis in clinically N0 oral tongue squamous cell carcinoma and its association with tumor staging, thickness, and differentiation. J Head Neck Physicians Surg. 2017;5(2):75.

35. Chen YW, Yu EH, Wu TH, Lo WL, Li WY, Kao SY. Histopathological factors affecting nodal metastasis in tongue cancer: analysis of 94 patients in Taiwan. Int J Oral Maxillofac Surg. 2008;37(10):912-6.

36. Chuang S-T, Chen C-C, Yang S-F, Chan L-P, Kao Y-H, Huang M-Y, et al. Tumor histologic grade as a risk factor for neck recurrence in patients with T1-2N0 early tongue cancer. Oral Oncology. 2020;106:104706.

37. Chung MK, Min JY, So YK, Ko YH, Jeong HS, Son YI, et al. Correlation between lymphatic vessel density and regional metastasis in squamous cell carcinoma of the tongue. Head & Neck. 2010;32(4):445-51.

38. Cracchiolo JR, Xu B, Migliacci JC, Katabi N, Pfister DG, Lee NY, et al. Patterns of recurrence in oral tongue cancer with perineural invasion. Head & Neck. 2018;40(6):1287-95.

39. D'Cruz AK, Dhar H, Vaish R, Hawaldar R, Gupta S, Pantvaidya G, et al. Depth of invasion in early oral cancers- is it an independent prognostic factor? European Journal of Surgical Oncology. 2021;47(8):1940-6.

40. De Silva RK, Siriwardena BSMS, Samaranayaka A, Abeyasinghe WAMUL, Tilakaratne WM. A model to predict nodal metastasis in patients with oral squamous cell carcinoma. PLoS ONE. 2018;13(8):e0201755.

41. Den Toom IJ, Janssen LM, Van Es RJJ, Karagozoglu KH, De Keizer B, Van Weert S, et al. Depth of invasion in patients with early stage oral cancer staged by sentinel node biopsy. Head & Neck. 2019;41(7):2100-6.

42. Dik EA, Willems SM, Ipenburg NA, Adriaansens SO, Rosenberg AJWP, Van Es RJJ. Resection of early oral squamous cell carcinoma with positive or close margins: Relevance of adjuvant treatment in relation to local recurrence. Oral Oncology. 2014;50(6):611-5.

43. Dillon JK, Brown CB, McDonald TM, Ludwig DC, Clark PJ, Leroux BG, et al. How Does the Close Surgical Margin Impact Recurrence and Survival When Treating Oral Squamous Cell Carcinoma? J Oral Maxillofac Surg. 2015;73(6):1182-8.

44. Doll C, Mrosk F, Wuester J, Runge A-S, Neumann F, Rubarth K, et al. Pattern of cervical lymph node metastases in squamous cell carcinoma of the upper oral cavity – How to manage the neck. Oral Oncology. 2022;130:105898.

45. Dourado MR, Miwa KYM, Hamada GB, Paranaíba LMR, Sawazaki‐Calone Í, Domingueti CB, et al. Prognostication for oral squamous cell carcinoma patients based on the tumour–stroma ratio and tumour budding. Histopathology. 2020;76(6):906-18.

46. Ebihara Y, Yoshida S, Nakahira M, Kogashiwa Y, Enoki Y, Kuba K, et al. Importance of tumor budding grade as independent prognostic factor for early tongue squamous cell carcinoma. Head & Neck. 2019;41(6):1809-15.

47. Ermer MA, Kirsch K, Bittermann G, Fretwurst T, Vach K, Metzger MC. Recurrence rate and shift in histopathological differentiation of oral squamous cell carcinoma – A long-term retrospective study over a period of 13.5 years. Journal of Cranio-Maxillofacial Surgery. 2015;43(7):1309-13.

48. Faisal M, Abu Bakar M, Sarwar A, Adeel M, Batool F, Malik KI, et al. Depth of invasion (DOI) as a predictor of cervical nodal metastasis and local recurrence in early stage squamous cell carcinoma of oral tongue (ESSCOT). PLoS ONE. 2018;13(8):e0202632.

49. Faustino SES, Tjioe KC, Assao A, Pereira MC, Carvalho AL, Kowalski LP, et al. Association of lymph vessel density with occult lymph node metastasis and prognosis in oral squamous cell carcinoma. BMC Oral Health. 2021;21(1):114.

50. Flörke C, Gülses A, Altmann C-R, Wiltfang J, Wieker H, Naujokat H. Clinicopathological Risk Factors for Contralateral Lymph Node Metastases in Intraoral Squamous Cell Carcinoma: A Study of 331 Cases. Current Oncology. 2021;28(3):1886-98.

51. Fu Y, Zhang X, Ding Z, Zhu N, Song Y, Zhang X, et al. Worst Pattern of Perineural Invasion Redefines the Spatial Localization of Nerves in Oral Squamous Cell Carcinoma. Front Oncol. 2021;11:766902.

52. Ganly I, Goldstein D, Carlson DL, Patel SG, O'Sullivan B, Lee N, et al. Long‐term regional control and survival in patients with “low‐risk,” early stage oral tongue cancer managed by partial glossectomy and neck dissection without postoperative radiation: The importance of tumor thickness. Cancer. 2013;119(6):1168-76.

53. Goodman M, Liu L, Ward K, Zhang J, Almon L, Su G, et al. Invasion characteristics of oral tongue cancer: Frequency of reporting and effect on survival in a population‐based study. Cancer. 2009;115(17):4010-20.

54. Grimm M. Prognostic value of clinicopathological parameters and outcome in 484 patients with oral squamous cell carcinoma: microvascular invasion (V+) is an independent prognostic factor for OSCC. Clin Transl Oncol. 2012;14(11):870-80.

55. Gueiros LA, Coletta RD, Kowalski LP, Lopes MA. Clinicopathological features and proliferation markers in tongue squamous cell carcinomas. Int J Oral Maxillofac Surg. 2011;40(5):510-5.

56. Haidari S, Obermeier KT, Kraus M, Otto S, Probst FA, Liokatis P. Nodal Disease and Survival in Oral Cancer: Is Occult Metastasis a Burden Factor Compared to Preoperatively Nodal Positive Neck? Cancers. 2022;14(17):4241.

57. Hakeem A, Pradhan S, Kannan R, Tubachi J. Clinical outcome of surgical treatment of T1-2 N0 squamous cell carcinoma of oral tongue with observation for the neck: Analysis of 176 cases. Ann Maxillofac Surg. 2016;6(2):235.

58. Hamada M, Ebihara Y, Yoshida S, Saito N, Enoki Y, Inoue H, et al. Prediction Formula for Pathological Depth of Invasion From Clinical Depth of Invasion in Tongue Squamous Cell Carcinoma (SCC) Stage I/II Cases. Cureus. 2023.

59. Ho YY, Wu TY, Cheng HC, Yang CC, Wu CH. The significance of tumor budding in oral cancer survival and its relevance to the eighth edition of the American Joint Committee on Cancer staging system. Head & Neck. 2019;41(9):2991-3001.

60. Hoda N, Bc R, Ghosh S, Ks S, B V, Nathani J. Cervical lymph node metastasis in squamous cell carcinoma of the buccal mucosa: a retrospective study on pattern of involvement and clinical analysis. Med Oral. 2021:e84-e9.

61. Hong K-O, Oh K-Y, Shin W-J, Yoon H-J, Lee J-I, Hong S-D. Tumor budding is associated with poor prognosis of oral squamous cell carcinoma and histologically represents an epithelial-mesenchymal transition process. Human Pathology. 2018;80:123-9.

62. Hori Y, Kubota A, Yokose T, Furukawa M, Matsushita T, Katsumata N, et al. Prognostic Role of Tumor‐Infiltrating Lymphocytes and Tumor Budding in Early Oral Tongue Carcinoma. The Laryngoscope. 2021;131(11):2512-8.

63. Hori Y, Kubota A, Yokose T, Furukawa M, Matsushita T, Oridate N. Association between pathological invasion patterns and late lymph node metastases in patients with surgically treated clinical No early oral tongue carcinoma. Head & Neck. 2020;42(2):238-43.

64. Hori Y, Kubota A, Yokose T, Furukawa M, Matsushita T, Takita M, et al. Predictive Significance of Tumor Depth and Budding for Late Lymph Node Metastases in Patients with Clinical N0 Early Oral Tongue Carcinoma. Head and Neck Pathol. 2017;11(4):477-86.

65. Hosni A, McMullen C, Huang SH, Xu W, Su J, Bayley A, et al. Lymph node ratio relationship to regional failure and distant metastases in oral cavity cancer. Radiotherapy and Oncology. 2017;124(2):225-31.

66. Huang S, Cai H, Song F, Zhu Y, Hou C, Hou J. Tumor–stroma ratio is a crucial histological predictor of occult cervical lymph node metastasis and survival in early-stage (cT1/2N0) oral squamous cell carcinoma. Int J Oral Maxillofac Surg. 2022;51(4):450-8.

67. Imai T, Satoh I, Matsumoto K, Asada Y, Yamazaki T, Morita S, et al. Retrospective observational study of occult cervical lymph-node metastasis in T1N0 tongue cancer. Japanese Journal of Clinical Oncology. 2017;47(2):130-6.

68. Jang JY, Choi N, Ko Y-H, Chung MK, Son Y-I, Baek C-H, et al. Differential Impact of Close Surgical Margin on Local Recurrence According to Primary Tumor Size in Oral Squamous Cell Carcinoma. Ann Surg Oncol. 2017;24(6):1698-706.

69. Jang JY, Kim MJ, Ryu G, Choi N, Ko Y-H, Jeong H-S. Prediction of Lymph Node Metastasis by Tumor Dimension Versus Tumor Biological Properties in Head and Neck Squamous Cell Carcinomas. Cancer Res Treat. 2016;48(1):54-62.

70. Jangir N, Singh A, Jain P, Khemka S. The predictive value of depth of invasion and tumor size on risk of neck node metastasis in squamous cell carcinoma of the oral cavity: A prospective study. Journal of Cancer Research and Therapeutics. 2022;18(4):977.

71. Jardim JF, Francisco ALN, Gondak R, Damascena A, Kowalski LP. Prognostic impact of perineural invasion and lymphovascular invasion in advanced stage oral squamous cell carcinoma. Int J Oral Maxillofac Surg. 2015;44(1):23-8.

72. Jardim JF, Galvis MM, Fabelo IR, Soares FA, Pinto CAL, Kowalski LP. Intratumoral lymphatic vascular density is an independent factor for disease-free and overall survival in advanced stage oral squamous cell carcinoma. Oral Surgery, Oral Medicine, Oral Pathology and Oral Radiology. 2021;132(5):580-8.

73. Jayasuriya NSS, Mannapperuma NT, Siriwardana S, Attygalla AM, DeSilva S, Jinadasa H, et al. Incidence of metastasis to level V lymph nodes in clinically positive necks among Sri Lankan patients with oral squamous cell carcinoma. British Journal of Oral and Maxillofacial Surgery. 2021;59(7):771-5.

74. Jensen D, Dabelsteen E, Specht L, Fiehn A, Therkildsen M, Jønson L, et al. Molecular profiling of tumour budding implicates TGFβ‐mediated epithelial–mesenchymal transition as a therapeutic target in oral squamous cell carcinoma. The Journal of Pathology. 2015;236(4):505-16.

75. Jerjes W, Upile T, Petrie A, Riskalla A, Hamdoon Z, Vourvachis M, et al. RCesleianrcihcopathological parameters, recurrence, locoregional and distant metastasis in 115 T1-T2 oral squamous cell carcinoma patients. 2010.

76. Jia J, Jia Mq, Zou Hx. Lingual lymph nodes in patients with squamous cell carcinoma of the tongue and the floor of the mouth. Head & Neck. 2018;40(11):2383-8.

77. Jones HB, Sykes A, Bayman N, Sloan P, Swindell R, Patel M, et al. The impact of lymphovascular invasion on survival in oral carcinoma. Oral Oncology. 2009;45(1):10-5.

78. Kakuguchi W, Ashikaga Y, Yanagawa-Matsuda A, Kuribayashi K, Shinohara S, Ogawa N, et al. Significant association of Yamamoto-Kohama classification and pathological depth of invasion with cervical lymph node metastasis in early-stage tongue squamous cell carcinoma (Stage I/II). Journal of Dental Sciences. 2023;18(4):1663-8.

79. Kallarakkal TG, Siriwardena BSMS, Samaranayaka A, De Silva R, Tilakaratne WM. A validated predictive model for risk of nodal metastasis in node negative oral squamous cell carcinoma of the buccal mucosa and tongue. J Oral Pathology Medicine. 2022;51(5):436-43.

80. Kane SV, Gupta M, Kakade AC, D' Cruz A. Depth of invasion is the most significant histological predictor of subclinical cervical lymph node metastasis in early squamous carcinomas of the oral cavity. European Journal of Surgical Oncology (EJSO). 2006;32(7):795-803.

81. Kapila SN. A Comparison of Clinicopathological Differences in Oral Squamous Cell Carcinoma in Patients Below and Above 40 Years of Age. J Clin Diagn Res. 2017.

82. Kato K, Miyazawa H, Kobayashi H, Kishikawa Y, Funaki H, Noguchi N, et al. The pattern and spread of invasion can predict late cervical lymph node metastasis in early tongue squamous cell carcinoma. Diagn Pathol. 2023;18(1):87.

83. Khan SA, Zia S, Naqvi SU, Adel H, Adil SO, Hussain M. Relationship of Oral Tumor Thickness with the rate of lymph node metastasis in Neck based on CT Scan. Pak J Med Sci. 2017;33(2).

84. Khwaja T, Tayaar AS, Acharya S, Bhushan J, Muddapur MV. Pattern of invasion as a factor in determining lymph node metastasis in oral squamous cell carcinoma. Journal of Cancer Research and Therapeutics. 2018;14(2):382-7.

85. Kim HC, Kameyama A, Kusukawa J. Clinicopathologic Parameters in Predicting Cervical Nodal Metastasis in Early Squamous Cell Carcinoma of the Oral Cavity. THE KURUME MEDICAL JOURNAL. 1993;40:183-92.

86. Kim RY, Helman JI, Braun TM, Ward BB. Increased Presence of Perineural Invasion in the Tongue and Floor of the Mouth: Could It Represent a More Aggressive Oral Squamous Cell Carcinoma, or Do Larger Aggressive Tumors Cause Perineural Invasion? J Oral Maxillofac Surg. 2019;77(4):852-8.

87. Kos M, Łuczak K, Brusco D, Engelke W. Impact of tumour characteristic and treatment modality on the local recurrence and the survival in patients with oral squamous cell carcinoma. Otolaryngologia Polska. 2008;62(6):722-6.

88. Kowalski LP, Bagietto Rr, Lara JRL, Santos RrL, Silva JoF, Magrin J. Prognostic significance of the distribution of neck node metastasis from oral carcinoma. Head & Neck. 2000;22(3):207-14.

89. Kurokawa H, Yamashita Y, Takeda S, Zhang M, Fukuyama H, Takahashi T. Risk factors for late cervical lymph node metastases in patients with stage I or II carcinoma of the tongue. Head & Neck. 2002;24(8):731-6.

90. Kurokawa H, Zhang M, Matsumoto S, Yamashita Y, Tomoyose T, Tanaka T, et al. The high prognostic value of the histologic grade at the deep invasive front of tongue squamous cell carcinoma. J Oral Pathology Medicine. 2005;34(6):329-33.

91. Lakhera KK, Nama Y, Maan P, Jindal R, Patel P, Singh S, et al. Worst Pattern of Invasion as a Predictor of Nodal Metastasis in Early-Stage Oral Squamous Cell Carcinoma. Indian J Surg Oncol. 2023;14(1):160-8.

92. Larsen SR, Johansen J, Sørensen JA, Krogdahl A. The prognostic significance of histological features in oral squamous cell carcinoma. J Oral Pathology Medicine. 2009;38(8):657-62.

93. Lau L, Eu D, Loh T, Ahmed Q, Lim CM. Histopathologic prognostic indices in tongue squamous cell carcinoma. European Archives of Oto-Rhino-Laryngology. 2021;278(7):2461-71.

94. Lee LY, De Paz D, Lin CY, Fan KH, Wang HM, Hsieh CH, et al. Prognostic impact of extratumoral perineural invasion in patients with oral cavity squamous cell carcinoma. Cancer Medicine. 2019;8(14):6185-94.

95. Li Y, Liu K, Ke Y, Zeng Y, Chen M, Li W, et al. Risk Factors Analysis of Pathologically Confirmed Cervical Lymph Nodes Metastasis in Oral Squamous Cell Carcinoma Patients with Clinically Negative Cervical Lymph Node: Results from a Cancer Center of Central China. J Cancer. 2019;10(13):3062-9.

96. Lim S-C, Zhang S, Ishii G, Endoh Y, Kodama K, Miyamoto S, et al. Predictive Markers for Late Cervical Metastasis in Stage I and II Invasive Squamous Cell Carcinoma of the Oral Tongue. Clinical Cancer Research. 2004;10(1):166-72.

97. Lin N-C, Hsu J-T, Tsai K-Y. Survival and clinicopathological characteristics of different histological grades of oral cavity squamous cell carcinoma: A single-center retrospective study. PLoS ONE. 2020;15(8):e0238103.

98. Liu B, Amaratunga R, Veness M, Wong E, Abdul-Razak M, Coleman H, et al. Tumor depth of invasion versus tumor thickness in guiding regional nodal treatment in early oral tongue squamous cell carcinoma. Oral Surgery, Oral Medicine, Oral Pathology and Oral Radiology. 2020;129(1):45-50.

99. Liu KYP, Durham JS, Wu J, Anderson DW, Prisman E, Poh CF. Nodal Disease Burden for Early-Stage Oral Cancer. JAMA Otolaryngol Head Neck Surg. 2016;142(11):1111.

100. Liu S-A, Wang C-C, Jiang R-S, Lee F-Y, Lin W-J, Lin J-C. Pathological features and their prognostic impacts on oral cavity cancer patients among different subsites – A singe institute’s experience in Taiwan. Scientific Reports. 2017;7(1):7451.

101. Lodder WL, Teertstra HJ, Tan IB, Pameijer FA, Smeele LE, Van Velthuysen M-LF, et al. Tumour thickness in oral cancer using an intra-oral ultrasound probe. Eur Radiol. 2011;21(1):98-106.

102. Loganathan P, Sayan A, Hsu DWK, Paraneetharan S, Ilankovan V. Squamous cell carcinoma of the anterior tongue: is tumour thickness an indicator for cervical metastasis? Int J Oral Maxillofac Surg. 2017;46(4):407-12.

103. Lu H-J, Chiu Y-W, Lan W-S, Peng C-Y, Tseng H-C, Hsin C-H, et al. Prediction Model of Distant Metastasis in Oral Cavity Squamous Cell Carcinoma With or Without Regional Lymphatic Metastasis. Front Oncol. 2022;11:713815.

104. Luksic I, Suton P. Predictive markers for delayed lymph node metastases and survival in early-stage oral squamous cell carcinoma: Predictive markers for delayed lymph node metastases and survival in early-stage oral cancer. Head & Neck. 2017;39(4):694-701.

105. Madana J, Laliberté F, Morand GB, Yolmo D, Black MJ, Mlynarek AM, et al. Computerized tomography based tumor-thickness measurement is useful to predict postoperative pathological tumor thickness in oral tongue squamous cell carcinoma. J of Otolaryngol - Head & Neck Surg. 2015;44(1):49.

106. Mafra RP, Serpa MS, Lima KCD, Silveira ÉJDD, Souza LBD, Pinto LP. Immunohistochemical analysis of lymphatic vessel density and mast cells in oral tongue squamous cell carcinoma. Journal of Cranio-Maxillofacial Surgery. 2018;46(12):2234-9.

107. Mair MD, Shetty R, Nair D, Mathur Y, Nair S, Deshmukh A, et al. Depth of invasion, size and number of metastatic nodes predicts extracapsular spread in early oral cancers with occult metastases. Oral Oncology. 2018;81:95-9.

108. Manjula BV, Augustine S, Selvam S, Mohan AM. Prognostic and Predictive Factors in Gingivo Buccal Complex Squamous Cell Carcinoma: Role of Tumor Budding and Pattern of Invasion. Indian J Otolaryngol Head Neck Surg. 2015;67(S1):98-104.

109. Marinelli LM, Chatzopoulos K, Marinelli JP, Chen TY, Collins AR, Sotiriou S, et al. Clinicopathologic predictors of survival in buccal squamous cell carcinoma. J Oral Pathology Medicine. 2020;49(9):857-64.

110. Mark Taylor S, Drover C, MacEachern R, Bullock M, Hart R, Psooy B, et al. Is preoperative ultrasonography accurate in measuring tumor thickness and predicting the incidence of cervical metastasis in oral cancer? Oral Oncology. 2010;46(1):38-41.

111. Marzouki H, Bukhari A, Al‑Ghamdi D, Abdullah R, Al‑Hajeili M, Khayyat S, et al. Worst pattern of invasion and other histopathological features in oral cancer as determinants of prognosis and survival rate: A retrospective cohort analysis. Oncol Lett. 2023;25(2):75.

112. Mascitti M, Zhurakivska K, Togni L, Caponio VCA, Almangush A, Balercia P, et al. Addition of the tumour–stroma ratio to the 8th edition American Joint Committee on Cancer staging system improves survival prediction for patients with oral tongue squamous cell carcinoma. Histopathology. 2020;77(5):810-22.

113. Matos LLD, Manfro G, Santos RVD, Stabenow E, Mello ESD, Alves VAF, et al. Tumor thickness as a predictive factor of lymph node metastasis and disease recurrence in T1N0 and T2N0 squamous cell carcinoma of the oral tongue. Oral Surgery, Oral Medicine, Oral Pathology and Oral Radiology. 2014;118(2):209-17.

114. Matsui T, Shigeta T, Umeda M, Komori T. Vascular endothelial growth factor C (VEGF-C) expression predicts metastasis in tongue cancer. Oral Surgery, Oral Medicine, Oral Pathology and Oral Radiology. 2015;120(4):436-42.

115. Matsushita Y, Yanamoto S, Takahashi H, Yamada S, Naruse T, Sakamoto Y, et al. A clinicopathological study of perineural invasion and vascular invasion in oral tongue squamous cell carcinoma. Int J Oral Maxillofac Surg. 2015;44(5):543-8.

116. Melchers LJ, Schuuring E, Van Dijk BAC, De Bock GH, Witjes MJH, Van Der Laan BFAM, et al. Tumour infiltration depth ⩾4mm is an indication for an elective neck dissection in pT1cN0 oral squamous cell carcinoma. Oral Oncology. 2012;48(4):337-42.

117. Michikawa C, Uzawa N, Kayamori K, Sonoda I, Ohyama Y, Okada N, et al. Clinical significance of lymphatic and blood vessel invasion in oral tongue squamous cell carcinomas. Oral Oncology. 2012;48(4):320-4.

118. Mijatov I, Kiralj A, Ilić M, Vučković N, Spasić A, Nikolić J, et al. Pathological tumor volume as a simple quantitative predictive factor of survival in oral squamous cell carcinoma. Oncol Lett. 2023;25(3):94.

119. Mneimneh WS, Xu B, Ghossein C, Alzumaili B, Sethi S, Ganly I, et al. Clinicopathologic Characteristics of Young Patients with Oral Squamous Cell Carcinoma. Head and Neck Pathol. 2021;15(4):1099-108.

120. Monevska DP, Janevska V, Naumovski S, Popovski V, Benedetti A, Bozovich S, et al. MULTIPLE PATHOHISTHOLOGICAL PARAMETERS INFLUENCING PROGNOSIS AND SURVIVAL OF ORAL CANCER PATIENTS.

121. Morand GB, Ikenberg K, Vital DG, Cardona I, Moch H, Stoeckli SJ, et al. Preoperative assessment of CD44‐mediated depth of invasion as predictor of occult metastases in early oral squamous cell carcinoma. Head & Neck. 2019;41(4):950-8.

122. Morimoto Y, Kurokawa H, Tanaka T, Yamashita Y, Kito S, Okabe S, et al. Correlation between the incidence of central nodal necrosis in cervical lymph node metastasis and the extent of differentiation in oral squamous cell carcinoma. Dentomaxillofacial Radiology. 2006;35(1):18-23.

123. Mücke T, Hölzle F, Wagenpfeil S, Wolff K-D, Kesting M. The role of tumor invasion into the mandible of oral squamous cell carcinoma. J Cancer Res Clin Oncol. 2011;137(1):165-71.

124. Mücke T, Kanatas A, Ritschl LM, Koerdt S, Tannapfel A, Wolff K-D, et al. Tumor thickness and risk of lymph node metastasis in patients with squamous cell carcinoma of the tongue. Oral Oncology. 2016;53:80-4.

125. Muhammad AY, Dhanani R, Salman S, Shaikh Z, Ghaloo SK, Ikram M. Depth of Invasion as a Predictor of Cervical Nodal Metastasis of Oral Tongue Squamous Cell Carcinoma: Findings From a Tertiary Care Center in Pakistan. Cureus. 2021.

126. Muttagi S, Patil B, Godhi A, Arora D, Hallikerimath, Sr., Kale A. Clinico-pathological factors affecting lymph node yield in Indian patients with locally advanced squamous cell carcinoma of mandibular Gingivo-Buccal sulcus. Indian J Cancer. 2016;53(2):239.

127. Naha K, Biedermann G, Nada A, Cousins J, Layfield L, Schnabel J. Preoperative Determination of Depth of Invasion in Oral Cavity Squamous Cell Carcinoma by Standard Cross-Sectional Imaging With Computed Tomography and Positron Emission Tomography/Computed Tomography. Cureus. 2023.

128. Nair AV, Meera M, Rajamma BM, Anirudh S, Nazer PK, Ramachandran PV. Preoperative ultrasonography for tumor thickness evaluation in guiding management in patients with early oral tongue squamous cell carcinoma. Indian Journal of Radiology and Imaging. 2018;28(02):140-5.

129. Nair D, Mair M, Singhvi H, Mishra A, Nair S, Agrawal J, et al. Perineural invasion: Independent prognostic factor in oral cancer that warrants adjuvant treatment. Head & Neck. 2018;40(8):1780-7.

130. Navarro Cuéllar I, Espías Alonso S, Alijo Serrano F, Herrera Herrera I, Zamorano León JJ, Del Castillo Pardo De Vera JL, et al. Depth of Invasion: Influence of the Latest TNM Classification on the Prognosis of Clinical Early Stages of Oral Tongue Squamous Cell Carcinoma and Its Association with Other Histological Risk Factors. Cancers. 2023;15(19):4882.

131. Nayanar S, Tripathy J, Duraisamy K, Babu S. Prognostic efficiency of clinicopathologic scoring to predict cervical lymph node metastasis in oral squamous cell carcinoma. J Oral Maxillofac Pathol. 2019;23(1):36.

132. Nguyen E, McKenzie J, Clarke R, Lou S, Singh T. The Indications for Elective Neck Dissection in T1N0M0 Oral Cavity Squamous Cell Carcinoma. J Oral Maxillofac Surg. 2021;79(8):1779-93.

133. Niu LX, Feng ZE, Wang DC, Zhang JY, Sun ZP, Guo CB. Prognostic factors in mandibular gingival squamous cell carcinoma: A 10-year retrospective study. Int J Oral Maxillofac Surg. 2017;46(2):137-43.

134. Noda Y, Ishida M, Ueno Y, Fujisawa T, Iwai H, Tsuta K. Novel pathological predictive factors for extranodal extension in oral squamous cell carcinoma: a retrospective cohort study based on tumor budding, desmoplastic reaction, tumor-infiltrating lymphocytes, and depth of invasion. BMC Cancer. 2022;22(1):402.

135. Nomura H, Uzawa K, Yamano Y, Fushimi K, Ishigami T, Kouzu Y, et al. Overexpression and altered subcellular localization of autophagy-related 16-like 1 in human oral squamous-cell carcinoma: correlation with lymphovascular invasion and lymph-node metastasis. Human Pathology. 2009;40(1):83-91.

136. Nseir S, Zeineh N, Capucha T, Israel Y, Emodi O, Abu El-Naaj I, et al. The impact of lymph node density as a predictive factor for survival and recurrence of tongue squamous cell carcinoma. Int J Oral Maxillofac Surg. 2022;51(4):441-9.

137. O-charoenrat P, Pillai G, Patel S, Fisher C, Archer D, Eccles S, et al. Tumour thickness predicts cervical nodal metastases and survival in early oral tongue cancer. Oral Oncology. 2003;39(4):386-90.

138. O'Brien CJ, Lauer CS, Fredricks S, Clifford AR, McNeil EB, Bagia JS, et al. Tumor thickness influences prognosis of T1 and T2 oral cavity cancer—but what thickness? Head & Neck. 2003;25(11):937-45.

139. Okada Y. Relationships of cervical lymph node metastasis to histopathological malignancy grade, tumor angiogenesis, and lymphatic invasion in tongue cancer. Odontology. 2010;98(2):153-9.

140. Okada Y, Mataga I, Katagiri M, Ishii K. An analysis of cervical lymph nodes metastasis in oral squamous cell carcinoma. Int J Oral Maxillofac Surg. 2003;32(3):284-8.

141. Okuyama K, Fukushima H, Naruse T, Yanamoto S, Tsuchihashi H, Umeda M. CD44 Variant 6 Expression and Tumor Budding in the Medullary Invasion Front of Mandibular Gingival Squamous Cell Carcinoma Are Predictive Factors for Cervical Lymph Node Metastasis. Pathol Oncol Res. 2019;25(2):603-9.

142. Oneyama T, Mataga I, Katagiri M. Cervical Lymph Node Metastasis and Histopathological Malignancy Grading of Primary Oral Squamous Cell Carcinoma. Asian Journal of Oral and Maxillofacial Surgery. 2009;21(3-4):81-7.

143. Ong W, Zhao R, Lui B, Tan W, Ebrahimi A, Clark JR, et al. Prognostic significance of lymph node density in squamous cell carcinoma of the tongue. Head & Neck. 2016;38(S1).

144. Pandit P, Patil R, Palwe V, Gandhe S, Manek D, Patil R, et al. Depth of Invasion, Lymphovascular Invasion, and Perineural Invasion as Predictors of Neck Node Metastasis in Early Oral Cavity Cancers. Indian J Otolaryngol Head Neck Surg. 2023;75(3):1511-6.

145. Patel RS, Clark JR, Dirven R, Wyten R, Gao K, O’Brien CJ. Prognostic factors in the surgical treatment of patients with oral carcinoma. ANZ J Surg. 2009;79(1-2):19-22.

146. Pedersen NJ, Jensen DH, Hedbäck N, Frendø M, Kiss K, Lelkaitis G, et al. Staging of early lymph node metastases with the sentinel lymph node technique and predictive factors in T1/T2 oral cavity cancer: A retrospective single‐center study. Head & Neck. 2016;38(S1).

147. Perisanidis C, Kornek G, Pöschl PW, Holzinger D, Pirklbauer K, Schopper C, et al. High neutrophil-to-lymphocyte ratio is an independent marker of poor disease-specific survival in patients with oral cancer. Med Oncol. 2013;30(1):334.

148. Petrovic I, Montero PH, Migliacci JC, Palmer FL, Ganly I, Patel SG, et al. Influence of bone invasion on outcomes after marginal mandibulectomy in squamous cell carcinoma of the oral cavity. Journal of Cranio-Maxillofacial Surgery. 2017;45(2):252-7.

149. Rahman N, Conn B. Evaluation of Histopathological Risk Model in a Cohort of Oral Squamous Cell Carcinoma Patients Treated with Accompanying Neck Dissection. Head and Neck Pathol. 2021;15(4):1156-61.

150. Reddy V, Wadhwan V, Reddy M, Venkatesh A. Controversies on Tumor Thickness Versus Nodal Metastasis in Oral Squamous Cell Carcinomas Revealed: A Histopathologist’s Perspective. Indian Journal of Medical and Paediatric Oncology. 2018;39(01):18-22.

151. Rhutso Y, Kakoti LM, Sharma JD, Kalita M. Significance of Pattern of Invasion in Tongue Squamous Cell Carcinoma—A Retrospective Study from a Regional Cancer Center of North-East India. South Asian J Cancer. 2022;11(02):140-5.

152. Rocchetti F, Tenore G, Montori A, Cassoni A, Cantisani V, Di Segni M, et al. Preoperative evaluation of tumor depth of invasion in oral squamous cell carcinoma with intraoral ultrasonography: a retrospective study. Oral Surgery, Oral Medicine, Oral Pathology and Oral Radiology. 2021;131(1):130-8.

153. Safi A-F, Kauke M, Grandoch A, Nickenig H-J, Zöller J, Kreppel M. The importance of lymph node ratio for patients with mandibular infiltration of oral squamous cell carcinoma. Journal of Cranio-Maxillofacial Surgery. 2018;46(6):1007-12.

154. Safi A-F, Kauke M, Grandoch A, Nickenig H-J, Zöller JE, Kreppel M. Analysis of clinicopathological risk factors for locoregional recurrence of oral squamous cell carcinoma – Retrospective analysis of 517 patients. Journal of Cranio-Maxillofacial Surgery. 2017;45(10):1749-53.

155. Sagowski C, Kehrl W, Metternich FU, Wenzel S. The prognostic impact of metastatic pattern of lymph nodes in patients with oral and oropharyngeal squamous cell carcinomas. European Archives of Oto-Rhino-Laryngology. 2004;261(5):270-5.

156. Sahoo A, Panda S, Mohanty N, Jena D, Mishra N, Surabhi, et al. Perinerural, lymphovascular and depths of invasion in extrapolating nodal metastasis in oral cancer. Clin Oral Invest. 2020;24(2):747-55.

157. Sakamoto Y, Matsushita Y, Yamada S-i, Yanamoto S, Shiraishi T, Asahina I, et al. Risk factors of distant metastasis in patients with squamous cell carcinoma of the oral cavity. Oral Surgery, Oral Medicine, Oral Pathology and Oral Radiology. 2016;121(5):474-80.

158. Sakata J, Yamana K, Yoshida R, Matsuoka Y, Kawahara K, Arita H, et al. Tumor budding as a novel predictor of occult metastasis in cT2N0 tongue squamous cell carcinoma. Human Pathology. 2018;76:1-8.

159. Salama AM, Valero C, Katabi N, Khimraj A, Yuan A, Zanoni DK, et al. Depth of invasion versus tumour thickness in early oral tongue squamous cell carcinoma: which measurement is the most practical and predictive of outcome? Histopathology. 2021;79(3):325-37.

160. Sarioglu S, Acara C, Akman FC, Dag N, Ecevit C, Ikiz AO, et al. Tumor budding as a prognostic marker in laryngeal carcinoma. Pathology - Research and Practice. 2010;206(2):88-92.

161. Sawazaki‐Calone I, Rangel A, Bueno A, Morais C, Nagai H, Kunz R, et al. The prognostic value of histopathological grading systems in oral squamous cell carcinomas. Oral Diseases. 2015;21(6):755-61.

162. Seki M, Sano T, Yokoo S, Oyama T. Tumour budding evaluated in biopsy specimens is a useful predictor of prognosis in patients with cN0 early stage oral squamous cell carcinoma. Histopathology. 2017;70(6):869-79.

163. Sekikawa S, Kawachi H, Ogane S, Saito H, Takano M, Nomura T, et al. Which Factors Affect the Long-Term Survival of Patients With Oral Squamous Cell Carcinoma With Distant Metastasis? J Oral Maxillofac Surg. 2020;78(3):469-78.

164. Shan J, Jiang R, Chen X, Zhong Y, Zhang W, Xie L, et al. Machine Learning Predicts Lymph Node Metastasis in Early-Stage Oral Tongue Squamous Cell Carcinoma. J Oral Maxillofac Surg. 2020;78(12):2208-18.

165. Sharma A, Boaz K, Natarajan S. Understanding patterns of invasion: a novel approach to assessment of podoplanin expression in the prediction of lymph node metastasis in oral squamous cell carcinoma. Histopathology. 2018;72(4):672-8.

166. Shen WR, Wang YP, Chang JYF, Yu SY, Chen HM, Chiang CP. Perineural invasion and expression of nerve growth factor can predict the progression and prognosis of oral tongue squamous cell carcinoma. J Oral Pathology Medicine. 2014;43(4):258-64.

167. Shetty DR. Histologic grading of the tumor/tissue interface to predict lymph node metastasis in squamous cell carcinoma of the tongue. Dent Med Probl. 2020;57(3):233-8.

168. Shimizu S, Miyazaki A, Sonoda T, Koike K, Ogi K, Kobayashi J-i, et al. Tumor budding is an independent prognostic marker in early stage oral squamous cell carcinoma: With special reference to the mode of invasion and worst pattern of invasion. PLoS ONE. 2018;13(4):e0195451.

169. Shinn JR, Wood CB, Colazo JM, Harrell FE, Rohde SL, Mannion K. Cumulative incidence of neck recurrence with increasing depth of invasion. Oral Oncology. 2018;87:36-42.

170. Simonetti O, Lucarini G, Rubini C, Zizzi A, Aspriello SD, Di Primio R, et al. Correlation between immunohistochemical staining of CEACAM1 and clinicopathological findings in oral pre-neoplastic lesions and squamous cell carcinoma. Med Mol Morphol. 2018;51(1):41-7.

171. Sindhura N, Vijayasree M, Sreedhar B, Sonia C, Dharani DK. Tumour Budding as a Predictive Factor for Lymph Node Metastases in Preoperative Oral Cancer Biopsies: A Retrospective Study. J Clin Diagn Res. 2023.

172. Singh A, Mair M, Singhvi H, Mahuvakar A, Nair D, Nair S, et al. Incidence, predictors and impact of positive bony margins in surgically treated T4 stage cancers of the oral cavity. Oral Oncology. 2019;90:8-12.

173. Siriwardena BSMS, Rambukewela IK, Pitakotuwage TN, Udagama MNGPK, Kumarasiri PVR, Tilakaratne WM. A Predictive Model to Determine the Pattern of Nodal Metastasis in Oral Squamous Cell Carcinoma. BioMed Res Int. 2018;2018:1-7.

174. Solomon J, Hinther A, Matthews TW, Nakoneshny SC, Hart R, Dort JC, et al. The impact of close surgical margins on recurrence in oral squamous cell carcinoma. J of Otolaryngol - Head & Neck Surg. 2021;50(1):9.

175. Son HJ, Roh JL, Cho KJ, Choi SH, Nam SY, Kim SY. Nodal factors predictive of recurrence and survival in patients with oral cavity squamous cell carcinoma. Clinical Otolaryngology. 2018;43(2):470-6.

176. Sowmya SV, Rao RS, Prasad K. Development of clinico-histopathological predictive model for the assessment of metastatic risk of oral squamous cell carcinoma. J Carcinog. 2020;19:2.

177. Sparano A, Weinstein G, Chalian A, Yodul M, Weber R. Multivariate Predictors of Occult Neck Metastasis in Early Oral Tongue Cancer. Otolaryngol--head neck surg. 2004;131(4):472-6.

178. Spoerl S, Gerken M, Mamilos A, Fischer R, Wolf S, Nieberle F, et al. Lymph node ratio as a predictor for outcome in oral squamous cell carcinoma: a multicenter population-based cohort study. Clin Oral Invest. 2021;25(4):1705-13.

179. Spoerl S, Spoerl S, Reil S, Gerken M, Ludwig N, Taxis J, et al. Prognostic Value of Perineural Invasion on Survival and Recurrence in Oral Squamous Cell Carcinoma. Diagn. 2022;12(5):1062.

180. Stoop CC, De Bree R, Rosenberg AJWP, Van Gemert JTM, Forouzanfar T, Van Cann EM. Locoregional recurrence rate and disease‐specific survival following marginal vs segmental resection for oral squamous cell carcinoma with mandibular bone invasion. Journal of Surgical Oncology. 2020;122(4):646-52.

181. Sundaram GA, Chokkattu JJ, Krishnan M, Kumar SP, M S, Lakshmanan S. Lymph Node Ratio as a Prognostic Factor for Oral Tongue Squamous Cell Carcinoma: A Retrospective Study. Cureus. 2023.

182. Suresh T, Hemalatha A, Harendra Kumar M, Azeem Mohiyuddin S. Evaluation of histomorphological and immunohistochemical parameters as biomarkers of cervical lymph node metastasis in squamous cell carcinoma of oral cavity: A retrospective study. J Oral Maxillofac Pathol. 2015;19(1):18.

183. Tai S-K, Li W-Y, Yang M-H, Chu P-Y, Wang Y-F, Chang PM-H. Perineural Invasion as a Major Determinant for the Aggressiveness Associated with Increased Tumor Thickness in T1–2 Oral Tongue and Buccal Squamous Cell Carcinoma. Ann Surg Oncol. 2013;20(11):3568-74.

184. Tam S, Amit M, Zafereo M, Bell D, Weber RS. Depth of invasion as a predictor of nodal disease and survival in patients with oral tongue squamous cell carcinoma. Head & Neck. 2019;41(1):177-84.

185. Tan A, Taskin T. Tumor Budding Should Be in Oral Cavity Cancer Reporting: A Retrospective Cohort Study Based on Tumor Microenvironment. Cancers. 2023;15(15):3905.

186. Tarsitano A, Del Corso G, Tardio ML, Marchetti C. Tumor Infiltration Depth as Predictor of Nodal Metastasis in Early Tongue Squamous Cell Carcinoma. J Oral Maxillofac Surg. 2016;74(3):523-7.

187. The International Consortium for Outcome Research in H, Neck C, Amit M, Yen TC, Liao CT, Binenbaum Y, et al. Clinical Nodal Stage is a Significant Predictor of Outcome in Patients with Oral Cavity Squamous Cell Carcinoma and Pathologically Negative Neck Metastases: Results of the International Consortium for Outcome Research. Ann Surg Oncol. 2013;20(11):3575-81.

188. Thiagarajan S, Nair S, Nair D, Chaturvedi P, Kane SV, Agarwal JP, et al. Predictors of prognosis for squamous cell carcinoma of oral tongue. Journal of Surgical Oncology. 2014;109(7):639-44.

189. Thompson SH. Cervical lymph node metastases of oral carcinoma related to the depth of invasion of the primary lesion. Journal of Surgical Oncology. 1986;31(2):120-2.

190. Ting K-C, Lee T-L, Li W-Y, Chang C-F, Chu P-Y, Wang Y-F, et al. Perineural invasion/lymphovascular invasion double positive predicts distant metastasis and poor survival in T3–4 oral squamous cell carcinoma. Scientific Reports. 2021;11(1):19770.

191. Van Lanschot CGF, Klazen YP, De Ridder MAJ, Mast H, Ten Hove I, Hardillo JA, et al. Depth of invasion in early stage oral cavity squamous cell carcinoma: The optimal cut-off value for elective neck dissection. Oral Oncology. 2020;111:104940.

192. Varsha B, Radhika M, Makarla S, Kuriakose M, Satya Kiran G, Padmalatha G. Perineural invasion in oral squamous cell carcinoma: Case series and review of literature. J Oral Maxillofac Pathol. 2015;19(3):335.

193. Verma R, Singh A, Chowdhury N, Joshi PP, Durgapal P, Rao S, et al. Evaluation of histomorphological parameters to predict occult nodal metastasis in early-stage oral squamous cell carcinoma. TJPATH. 2021.

194. Vidiri A, Panfili M, Boellis A, Cristalli G, Gangemi E, Pellini R, et al. The role of MRI-derived depth of invasion in staging oral tongue squamous cell carcinoma: inter-reader and radiological–pathological agreement. Acta Radiol. 2020;61(3):344-52.

195. Wang H-M, Liao C-T, Yen T-C, Chen S-J, Lee L-Y, Hsieh C-H, et al. Clues toward precision medicine in oral squamous cell carcinoma: utility of next-generation sequencing for the prognostic stratification of high-risk patients harboring neck lymph node extracapsular extension. Oncotarget. 2016;7(39):63082-92.

196. Wang K, Veivers D. Tumour thickness as a determinant of nodal metastasis in oral tongue carcinoma. ANZ J Surg. 2017;87(9):720-4.

197. Wang S, Li T, Liu H, Wei W, Yang Y, Wang C, et al. A Combined Prediction Model for Lymph Node Metastasis Based on a Molecular Panel and Clinicopathological Factors in Oral Squamous Cell Carcinoma. Front Oncol. 2021;11:660615.

198. Warburton G, Nikitakis NG, Roberson P, Marinos NJ, Wu T, Sauk JJ, et al. Histopathological and Lymphangiogenic Parameters in Relation to Lymph Node Metastasis in Early Stage Oral Squamous Cell Carcinoma. J Oral Maxillofac Surg. 2007;65(3):475-84.

199. Wedemeyer I, Kreppel M, Scheer M, Zöller J, Büttner R, Drebber U. Histopathological assessment of tumour regression, nodal stage and status of resection margins determines prognosis in patients with oral squamous cell carcinoma treated with neoadjuvant radiochemotherapy. Oral Diseases. 2014;20(3).

200. Wei PY, Li WY, Tai SK. Discrete Perineural Invasion Focus Number in Quantification for T1‐T2 Oral Squamous Cell Carcinoma. Otolaryngol--head neck surg. 2019;160(4):635-41.

201. Weimar EAM, Huang SH, Lu L, O'Sullivan B, Perez-Ordonez B, Weinreb I, et al. Radiologic-Pathologic Correlation of Tumor Thickness and Its Prognostic Importance in Squamous Cell Carcinoma of the Oral Cavity: Implications for the Eighth Edition Tumor, Node, Metastasis Classification. American Journal of Neuroradiology. 2018;39(10):1896-902.

202. Woolgar JA, Rogers SN, Lowe D, Brown JS, Vaughan ED. Cervical lymph node metastasis in oral cancer: the importance of even microscopic extracapsular spread. Oral Oncology. 2003;39(2):130-7.

203. Wu K, Wei J, Liu Z, Yu B, Yang X, Zhang C, et al. Can pattern and depth of invasion predict lymph node relapse and prognosis in tongue squamous cell carcinoma. BMC Cancer. 2019;19(1):714.

204. Xie N, Wang C, Liu X, Li R, Hou J, Chen X, et al. Tumor budding correlates with occult cervical lymph node metastasis and poor prognosis in clinical early‐stage tongue squamous cell carcinoma. J Oral Pathology Medicine. 2015;44(4):266-72.

205. Xie N, Yu P, Liu H, Liu X, Hou J, Chen X, et al. Validation of the International Tumor Budding Consensus Conference (2016) recommendations in oral tongue squamous cell carcinoma. J Oral Pathology Medicine. 2019;48(6):451-8.

206. Xu B, Salama AM, Valero C, Yuan A, Khimraj A, Saliba M, et al. The prognostic role of histologic grade, worst pattern of invasion, and tumor budding in early oral tongue squamous cell carcinoma: a comparative study. Virchows Arch. 2021;479(3):597-606.

207. Yamada S-i, Otsuru M, Yanamoto S, Hasegawa T, Aizawa H, Kamata T, et al. Progression level of extracapsular spread and tumor budding for cervical lymph node metastasis of OSCC. Clin Oral Invest. 2018;22(3):1311-8.

208. Yamagata K, Fukuzawa S, Kanno N, Uchida F, Yanagawa T, Bukawa H. Is Lymph Node Ratio a Prognostic Factor for Patients With Oral Squamous Cell Carcinoma? J Oral Maxillofac Surg. 2019;77(7):1510-9.

209. Yamakawa N, Kirita T, Umeda M, Yanamoto S, Ota Y, Otsuru M, et al. Tumor budding and adjacent tissue at the invasive front correlate with delayed neck metastasis in clinical early‐stage tongue squamous cell carcinoma. Journal of Surgical Oncology. 2019;119(3):370-8.

210. Yamamoto N, Osaka R, Ogane S, Sugahara K, Yamamoto M, Muramatsu K, et al. Clinicopathological study of tumor depth in tongue squamous cell carcinoma. Journal of Oral and Maxillofacial Surgery, Medicine, and Pathology. 2014;26(2):118-21.

211. Yang H, Son N-H, Lee SH, Kim D, Kim HJ, Cha I-h, et al. Predictive modelling of level IIb lymph node metastasis in oral squamous cell carcinoma. Scientific Reports. 2021;11(1):17562.

212. Yang X, Tian X, Wu K, Liu W, Li S, Zhang Z, et al. Prognostic impact of perineural invasion in early stage oral tongue squamous cell carcinoma: Results from a prospective randomized trial. Surgical Oncology. 2018;27(2):123-8.

213. Yoshid S, Shimo T, Murase Y. The Prognostic Implications of Bone Invasion in Gingival Squamous Cell Carcinoma. AR. 2018;38(2).

214. Yoshizawa K, Kimura Y, Moroi A, Ishii H, Sakurai D, Saitoh M, et al. Loss of intercellular bridges in the depth of invasion measurement area is a novel negative prognostic factor for oral squamous cell carcinoma: A retrospective study. Oral Surgery, Oral Medicine, Oral Pathology and Oral Radiology. 2022;134(1):84-92.

215. Yu P, Wang W, Zhuang Z, Xie N, Xu J, Wang C, et al. A novel prognostic model for tongue squamous cell carcinoma based on the characteristics of tumour and its microenvironment: iBD score. Histopathology. 2019;74(5):766-79.

216. Zenga J, Divi V, Stadler M, Massey B, Campbell B, Shukla M, et al. Lymph node yield, depth of invasion, and survival in node-negative oral cavity cancer. Oral Oncology. 2019;98:125-31.

217. Zhang S, Wang X, Gupta A, Fang X, Wang L, Zhang C. Expression of IL-17 with tumor budding as a prognostic marker in oral squamous cell carcinoma.
