# Supplementary material for: Histopathological predictors of lymph node metastasis in oral cavity squamous cell carcinoma: a systematic review and meta-analysis
Source: Front Oncol. 2024 May 14;14:1401211. doi: 10.3389/fonc.2024.1401211 (PMC11148647; doi:10.3389/fonc.2024.1401211)
Supplement: Supplementary file 3 [file Table_2.docx]

| **Study ID** | **Supplementary Table 2: The Cochrane Collaboration’s tool for assessing risk of bias of RCT** | | | | | | |  |
| --- | --- | --- | --- | --- | --- | --- | --- | --- |
|  | **Random sequence generation  (Selection bias)** | **Allocation concealment (Selection bias)** | **Blinding of participants and personnel (Performance bias)** | **Blinding of outcome assessment  (Detection bias)** | **Incomplete outcome data (Attrition bias)** | **Selective reporting (Reporting bias)** | **Other Bias** |  |
|  | Low \ High \ Unclear risk of bias | Low \ High \ Unclear risk of bias | Low \ High \ Unclear risk of bias | Low \ High \ Unclear risk of bias | Low \ High \ Unclear risk of bias | Low \ High \ Unclear risk of bias | Low \ High \ Unclear risk of bias |  |
|  |  |  |  |  |  |  |  |  |
| Yang 2018 | Low risk | Low risk | Unclear risk | Low risk | Low risk | Low risk | High risk |  |
