# Supplementary material for: Histopathological predictors of lymph node metastasis in oral cavity squamous cell carcinoma: a systematic review and meta-analysis
Source: Front Oncol. 2024 May 14;14:1401211. doi: 10.3389/fonc.2024.1401211 (PMC11148647; doi:10.3389/fonc.2024.1401211)
Supplement: Supplementary file 4 [file Table_3.docx]

| **ID** | **Supplementary Table 3: NIH Quality Assessment Tool for Observational Cohort and Cross-Sectional Studies** | | | | | | | | | | | | | | | **Quality rating: Good  (11-14 )  or  Fair  (7.5-10.5) or Poor  (0-7),  Yes = 1 // No = 0.5 // NR & NA & CD = 0** |
| --- | --- | --- | --- | --- | --- | --- | --- | --- | --- | --- | --- | --- | --- | --- | --- | --- |
|  | **1. Was the research question or objective in this paper clearly stated?** | **2. Were eligibility/selection criteria for the study population prespecified and clearly described?** | **3. Were the participants in the study representative of those who would be eligible for the test/service/intervention in the general or clinical population of interest?** | **4. Were all eligible participants that met the prespecified entry criteria enrolled?** | **5. Was the sample size sufficiently large to provide confidence in the findings?** | **6. For the analyses in this paper, were the exposure(s) of interest measured prior to the outcome(s) being measured?** | **7. Was the time frame sufficient so that one could reasonably expect to see an association between exposure and outcome if it existed?** | **8. For exposures that can vary in amount or level, did the study examine different levels of the exposure as related to the outcome (e.g., categories of exposure or exposure measured as continuous variable)?** | **9. Were the exposure measures (independent variables) clearly defined, valid, reliable, and implemented consistently across all study participants?** | **10. Was the exposure(s) assessed more than once over time?** | **11. Were the outcome measures prespecified, clearly defined, valid, reliable, and assessed consistently across all study participants?** | **12. Were the people assessing the outcomes blinded to the participants' exposures/interventions?** | **13. Was the loss to follow-up after baseline 20% or less? Were those lost to follow-up accounted for in the analysis?** | **14. Were key potential confounding variables measured and adjusted statistically for their impact on the relationship between exposure(s) and outcome(s)?** | **Total scores** |  |
|  | **Yes / No / Not reported (NR) or cannot determine (CD) or not applicable (NA)** | **Yes / No / Not reported (NR) or cannot determine (CD) or not applicable (NA)** | **Yes / No / Not reported (NR) or cannot determine (CD) or not applicable (NA)** | **Yes / No / Not reported (NR) or cannot determine (CD) or not applicable (NA)** | **Yes / No / Not reported (NR) or cannot determine (CD) or not applicable (NA)** | **Yes / No / Not reported (NR) or cannot determine (CD) or not applicable (NA)** | **Yes / No / Not reported (NR) or cannot determine (CD) or not applicable (NA)** | **Yes / No / Not reported (NR) or cannot determine (CD) or not applicable (NA)** | **Yes / No / Not reported (NR) or cannot determine (CD) or not applicable (NA)** | **Yes / No / Not reported (NR) or cannot determine (CD) or not applicable (NA)** | **Yes / No / Not reported (NR) or cannot determine (CD) or not applicable (NA)** | **Yes / No / Not reported (NR) or cannot determine (CD) or not applicable (NA)** | **Yes / No / Not reported (NR) or cannot determine (CD) or not applicable (NA)** | **Yes / No / Not reported (NR) or cannot determine (CD) or not applicable (NA)** |  |  |
| **Aaboubout 2021** | Yes | Yes | Yes | NR | NR | Yes | Yes | Yes | Yes | NR | Yes | NR | Yes | Yes | **10** | **Fair** |
| **Acharya 2021** | Yes | Yes | Yes | NR | NR | Yes | NR | Yes | Yes | NR | Yes | NR | Yes | Yes | **9** | **Fair** |
| **Acharya 2023** | Yes | Yes | Yes | No | NR | Yes | Yes | Yes | Yes | NR | NR | Yes | Yes | Yes | **10.5** | **Fair** |
| **Adel 2015** | Yes | Yes | Yes | Yes | Yes | Yes | Yes | Yes | Yes | NR | Yes | NR | Yes | Yes | **12** | **Good** |
| **Adel 2016** | Yes | Yes | Yes | Yes | NR | Yes | Yes | NR | Yes | NR | Yes | NR | Yes | Yes | **10** | **Fair** |
| **Aires 2017** | Yes | Yes | Yes | Yes | NR | Yes | Yes | Yes | Yes | NR | NR | NR | Yes | Yes | **10** | **Fair** |
| **Aivazian 2014** | Yes | Yes | Yes | Yes | Yes | Yes | Yes | Yes | Yes | NR | NR | NR | Yes | Yes | **11** | **Good** |
| **Akhter 2011** | Yes | Yes | Yes | Yes | NR | Yes | Yes | Yes | Yes | NR | Yes | NR | Yes | Yes | **11** | **Good** |
| **Amit 2013** | Yes | Yes | Yes | NR | Yes | Yes | Yes | Yes | Yes | NR | Yes | NR | Yes | Yes | **11** | **Good** |
| **Angadi 2015** | Yes | Yes | Yes | Yes | NR | Yes | Yes | Yes | Yes | NR | Yes | Yes | Yes | Yes | **12** | **Good** |
| **Arora 2017** | Yes | Yes | Yes | NR | Yes | Yes | Yes | Yes | Yes | NR | Yes | NR | Yes | Yes | **11** | **Good** |
| **Arun 2021** | Yes | Yes | Yes | NR | Yes | Yes | Yes | Yes | Yes | NR | Yes | NR | Yes | Yes | **11** | **Good** |
| **Asakage 1998** | Yes | Yes | Yes | Yes | NR | Yes | Yes | Yes | NR | NR | NR | NR | Yes | Yes | **9** | **Fair** |
| **Azaidan 2014** | Yes | Yes | Yes | NR | NR | Yes | Yes | Yes | Yes | NR | Yes | Yes | Yes | Yes | **11** | **Good** |
| **Bachar 2012** | Yes | Yes | Yes | NR | NR | Yes | Yes | Yes | Yes | NR | Yes | NR | Yes | Yes | **10** | **Fair** |
| **Bae 2020** | Yes | Yes | Yes | Yes | NR | Yes | Yes | NR | Yes | NR | Yes | NR | Yes | Yes | **10** | **Fair** |
| **Balasubramanian 2014** | Yes | Yes | Yes | Yes | Yes | Yes | Yes | Yes | Yes | NR | Yes | NR | Yes | Yes | **12** | **Good** |
| **Balla 2020** | Yes | NR | Yes | NR | NR | Yes | Yes | Yes | Yes | NR | Yes | NR | Yes | Yes | **9** | **Fair** |
| **Beggan 2016** | Yes | Yes | Yes | Yes | NR | Yes | Yes | Yes | Yes | NR | NR | NR | Yes | Yes | **10** | **Fair** |
| **Bera 2022** | Yes | Yes | Yes | NR | Yes | Yes | Yes | Yes | Yes | NR | Yes | Yes | Yes | Yes | **12** | **Good** |
| **Berger 2015** | Yes | Yes | Yes | NR | NR | Yes | Yes | Yes | NR | NR | NR | NR | Yes | Yes | **8** | **Fair** |
| **Bhatlawande 2019** | Yes | Yes | Yes | NR | NR | Yes | Yes | Yes | Yes | NR | Yes | NR | Yes | Yes | **10** | **Fair** |
| **Bhatta 2020** | Yes | Yes | Yes | NR | NR | Yes | Yes | Yes | Yes | NR | Yes | NR | Yes | Yes | **10** | **Fair** |
| **Bjerkli 2020** | Yes | Yes | Yes | NR | Yes | Yes | Yes | Yes | Yes | NR | Yes | NR | Yes | Yes | **11** | **Good** |
| **Boxberg 2017** | Yes | Yes | Yes | NR | NR | Yes | Yes | Yes | Yes | NR | Yes | NR | Yes | Yes | **10** | **Fair** |
| **Brockhoff 2017** | Yes | Yes | Yes | Yes | NR | Yes | Yes | Yes | Yes | NR | NR | NR | Yes | Yes | **10** | **Fair** |
| **Brown 2002** | Yes | Yes | Yes | NR | NR | Yes | Yes | Yes | Yes | NR | NR | NR | Yes | Yes | **9** | **Fair** |
| **Caponio 2021** | Yes | Yes | Yes | NR | NR | Yes | Yes | Yes | Yes | NR | NR | Yes | Yes | Yes | **10** | **Fair** |
| **Chandavarkar 2015** | Yes | Yes | Yes | NR | NR | Yes | Yes | Yes | Yes | NR | Yes | NR | Yes | Yes | **10** | **Fair** |
| **Chandler 2011** | Yes | NR | Yes | NR | NR | Yes | Yes | Yes | Yes | NR | Yes | NR | Yes | NR | **8** | **Fair** |
| **Chang 2010** | Yes | Yes | Yes | NR | NR | Yes | Yes | Yes | Yes | NR | NR | Yes | No | Yes | **9.5** | **Fair** |
| **Chang 2017** | Yes | Yes | Yes | NR | NR | Yes | Yes | Yes | NR | NR | NR | NR | Yes | Yes | **8** | **Fair** |
| **Chang 2019** | Yes | Yes | Yes | NR | NR | Yes | Yes | Yes | Yes | NR | NR | NR | Yes | Yes | **9** | **Fair** |
| **Chatterjee 2019** | Yes | Yes | Yes | Yes | NR | Yes | Yes | Yes | Yes | NR | Yes | Yes | Yes | Yes | **12** | **Good** |
| **Chaudhary 2018** | Yes | Yes | Yes | Yes | NR | Yes | Yes | Yes | Yes | NR | Yes | NR | Yes | Yes | **11** | **Good** |
| **Chen 2008** | Yes | Yes | Yes | Yes | NR | Yes | Yes | Yes | Yes | NR | Yes | NR | Yes | Yes | **11** | **Good** |
| **Chuang 2020** | Yes | Yes | Yes | NR | NR | Yes | Yes | Yes | Yes | NR | Yes | NR | Yes | Yes | **10** | **Fair** |
| **Chung 2010** | Yes | Yes | Yes | NR | NR | Yes | Yes | Yes | Yes | NR | Yes | NR | Yes | Yes | **10** | **Fair** |
| **Cracchiolo 2018** | Yes | Yes | Yes | NR | NR | Yes | Yes | Yes | Yes | NR | NR | Yes | Yes | Yes | **10** | **Fair** |
| **Cuéllar 2023** | Yes | Yes | Yes | NR | NR | Yes | Yes | Yes | NR | NR | NR | NR | Yes | Yes | **8** | **Fair** |
| **D_Cruz 2021** | Yes | Yes | Yes | NR | Yes | Yes | Yes | Yes | Yes | NR | NR | NR | Yes | Yes | **9** | **Fair** |
| **Desilva 2018** | Yes | NR | Yes | NR | Yes | Yes | Yes | Yes | NR | NR | NR | NR | Yes | NR | **7** | **Poor** |
| **Dik 2014** | Yes | Yes | Yes | NR | NR | Yes | Yes | NR | Yes | NR | NR | NR | Yes | Yes | **8** | **Fair** |
| **Dillon 2015** | Yes | Yes | Yes | Yes | NR | Yes | Yes | Yes | Yes | NR | NR | NR | Yes | Yes | **10** | **Fair** |
| **Doll 2022** | Yes | Yes | Yes | Yes | NR | Yes | NR | Yes | NR | NR | NR | NR | Yes | Yes | **8** | **Fair** |
| **Dourado 2020** | Yes | Yes | Yes | NR | NR | Yes | Yes | Yes | Yes | NR | Yes | NR | Yes | Yes | **10** | **Fair** |
| **Ebihara 2019** | Yes | Yes | Yes | NR | NR | Yes | Yes | Yes | Yes | NR | Yes | NR | Yes | Yes | **10** | **Fair** |
| **Ermer 2015** | Yes | Yes | Yes | NR | Yes | Yes | Yes | Yes | NR | NR | NR | NR | Yes | Yes | **9** | **Fair** |
| **Faisal 2018** | Yes | Yes | Yes | NR | NR | Yes | Yes | Yes | Yes | NR | Yes | NR | Yes | Yes | **10** | **Fair** |
| **Faustino 2021** | Yes | Yes | Yes | NR | NR | Yes | Yes | Yes | Yes | NR | Yes | Yes | Yes | Yes | **11** | **Good** |
| **Flörke 2021** | Yes | Yes | Yes | NR | NR | Yes | Yes | Yes | Yes | NR | NR | NR | Yes | Yes | **9** | **Fair** |
| **Fu 2021** | Yes | Yes | Yes | NR | NR | Yes | Yes | Yes | Yes | NR | Yes | NR | Yes | Yes | **10** | **Fair** |
| **Ganly 2013** | Yes | Yes | Yes | NR | NR | Yes | Yes | Yes | Yes | NR | Yes | NR | Yes | Yes | **10** | **Fair** |
| **Goodman 2009** | Yes | Yes | Yes | Yes | NR | Yes | Yes | NR | NR | NR | NR | NR | Yes | Yes | **8** | **Fair** |
| **Grimm 2012** | Yes | Yes | Yes | Yes | Yes | Yes | Yes | Yes | Yes | NR | Yes | Yes | Yes | Yes | **13** | **Good** |
| **Gueiros 2011** | Yes | Yes | Yes | NR | NR | Yes | Yes | Yes | Yes | NR | Yes | NR | Yes | Yes | **10** | **Fair** |
| **Haidari 2022** | Yes | Yes | Yes | NR | NR | Yes | Yes | Yes | NR | NR | Yes | NR | Yes | Yes | **9** | **Fair** |
| **Hakeem 2016** | Yes | Yes | Yes | NR | NR | Yes | Yes | Yes | Yes | NR | Yes | NR | Yes | Yes | **10** | **Fair** |
| **Hamada 2023** | Yes | Yes | Yes | NR | NR | Yes | Yes | Yes | Yes | NR | NR | NR | Yes | Yes | **9** | **Fair** |
| **Ho 2019** | Yes | Yes | Yes | NR | NR | Yes | Yes | Yes | Yes | NR | Yes | Yes | Yes | Yes | **11** | **Good** |
| **Hoda 2021** | Yes | Yes | Yes | NR | NR | Yes | Yes | Yes | Yes | NR | Yes | NR | Yes | Yes | **10** | **Fair** |
| **Hong 2018** | Yes | Yes | Yes | NR | NR | Yes | Yes | Yes | Yes | NR | NR | NR | Yes | Yes | **9** | **Fair** |
| **Hori 2017** | Yes | Yes | Yes | NR | NR | Yes | Yes | Yes | Yes | NR | Yes | Yes | Yes | Yes | **11** | **Good** |
| **Hori 2020** | Yes | Yes | Yes | NR | NR | Yes | Yes | Yes | Yes | NR | Yes | Yes | Yes | Yes | **11** | **Good** |
| **Hori 2021** | Yes | Yes | Yes | NR | NR | Yes | Yes | Yes | Yes | NR | Yes | Yes | Yes | Yes | **11** | **Good** |
| **Hosni 2017** | Yes | Yes | Yes | Yes | Yes | Yes | Yes | Yes | Yes | NR | Yes | NR | Yes | Yes | **12** | **Good** |
| **Huang 2019** | Yes | Yes | Yes | NR | Yes | Yes | Yes | Yes | Yes | NR | Yes | NR | Yes | Yes | **11** | **Good** |
| **Imai 2016** | Yes | Yes | Yes | NR | NR | Yes | Yes | Yes | Yes | NR | Yes | Yes | No | Yes | **10.5** | **Fair** |
| **Jang 2016** | Yes | Yes | Yes | Yes | NR | Yes | Yes | Yes | Yes | NR | NR | NR | Yes | Yes | **10** | **Fair** |
| **Jang 2016 (2)** | Yes | Yes | Yes | Yes | Yes | Yes | Yes | Yes | Yes | NR | NR | NR | Yes | Yes | **11** | **Good** |
| **Jangir 2021** | Yes | Yes | Yes | NR | NR | Yes | Yes | Yes | Yes | NR | Yes | NR | Yes | Yes | **10** | **Fair** |
| **Jardim 2021** | Yes | Yes | Yes | NR | NR | Yes | Yes | Yes | Yes | NR | Yes | Yes | Yes | Yes | **11** | **Good** |
| **Jardim 2015** | Yes | Yes | Yes | NR | NR | Yes | Yes | Yes | Yes | NR | NR | NR | Yes | Yes | **9** | **Fair** |
| **Jayasuriya 2020** | Yes | Yes | Yes | NR | Yes | Yes | Yes | Yes | Yes | NR | Yes | NR | Yes | Yes | **11** | **Good** |
| **Jensen 2015** | Yes | Yes | Yes | NR | NR | Yes | Yes | Yes | Yes | NR | Yes | NR | Yes | Yes | **10** | **Fair** |
| **Jerjes 2010** | Yes | Yes | Yes | Yes | NR | Yes | Yes | Yes | Yes | NR | Yes | NR | Yes | Yes | **11** | **Good** |
| **Jia 2018** | Yes | Yes | Yes | NR | NR | Yes | Yes | Yes | Yes | NR | Yes | NR | Yes | Yes | **10** | **Fair** |
| **Jones 2009** | Yes | Yes | Yes | Yes | NR | Yes | Yes | Yes | Yes | NR | Yes | NR | Yes | Yes | **11** | **Good** |
| **Kakuguchi 2023** | Yes | Yes | Yes | Yes | NR | Yes | Yes | Yes | Yes | NR | Yes | NR | Yes | Yes | **11** | **Good** |
| **Kallarakkal 2022** | Yes | Yes | Yes | Yes | NR | Yes | Yes | Yes | NR | NR | Yes | NR | Yes | Yes | **10** | **Fair** |
| **Kane 2006** | Yes | Yes | Yes | NR | NR | Yes | Yes | Yes | Yes | NR | Yes | NR | Yes | Yes | **10** | **Fair** |
| **Kapila 2017** | Yes | Yes | Yes | NR | NR | Yes | Yes | Yes | Yes | NR | Yes | NR | Yes | Yes | **10** | **Fair** |
| **Kato 2023** | Yes | Yes | Yes | NR | NR | Yes | Yes | Yes | NR | NR | NR | NR | Yes | Yes | **8** | **Fair** |
| **Khan 2017** | Yes | Yes | Yes | NR | NR | Yes | Yes | Yes | NR | NR | Yes | NR | Yes | Yes | **9** | **Fair** |
| **Khwaja 2016** | Yes | Yes | Yes | NR | NR | Yes | Yes | Yes | NR | NR | NR | NR | Yes | Yes | **8** | **Fair** |
| **Kim 1993** | Yes | Yes | Yes | NR | NR | Yes | Yes | Yes | NR | NR | NR | NR | Yes | Yes | **8** | **Fair** |
| **Kim 2018** | Yes | Yes | Yes | Yes | Yes | Yes | Yes | Yes | NR | NR | NR | NR | Yes | Yes | **10** | **Fair** |
| **Kos 2008** | Yes | Yes | Yes | NR | NR | Yes | Yes | Yes | NR | NR | NR | NR | Yes | Yes | **8** | **Fair** |
| **Kowalski 2000** | Yes | Yes | Yes | Yes | Yes | Yes | Yes | Yes | NR | NR | NR | NR | Yes | Yes | **10** | **Fair** |
| **Kurokawa 2002** | Yes | Yes | Yes | NR | NR | Yes | Yes | Yes | Yes | NR | Yes | NR | Yes | Yes | **10** | **Fair** |
| **Kurokawa 2005** | Yes | Yes | Yes | Yes | NR | Yes | Yes | Yes | Yes | NR | Yes | Yes | Yes | Yes | **12** | **Good** |
| **Lakhera 2022** | Yes | Yes | Yes | NR | NR | Yes | Yes | Yes | Yes | NR | NR | Yes | Yes | Yes | **10** | **Fair** |
| **Larsen 2009** | Yes | Yes | Yes | NR | NR | Yes | Yes | Yes | Yes | NR | Yes | NR | Yes | Yes | **10** | **Fair** |
| **Lau 2021** | Yes | Yes | Yes | Yes | NR | Yes | Yes | Yes | Yes | NR | Yes | NR | Yes | Yes | **11** | **Good** |
| **Lee 2019** | Yes | Yes | Yes | Yes | Yes | Yes | Yes | Yes | Yes | NR | Yes | NR | Yes | Yes | **12** | **Good** |
| **Li 2019** | Yes | Yes | Yes | NR | NR | Yes | Yes | Yes | NR | NR | Yes | NR | Yes | Yes | **9** | **Fair** |
| **Lim 2004** | Yes | Yes | Yes | NR | NR | Yes | Yes | Yes | Yes | NR | NR | NR | Yes | Yes | **9** | **Fair** |
| **Lin 2020** | Yes | Yes | Yes | NR | Yes | Yes | Yes | Yes | Yes | NR | Yes | NR | Yes | Yes | **11** | **Good** |
| **Liu 2016** | Yes | Yes | Yes | NR | NR | Yes | Yes | Yes | NR | NR | NR | NR | Yes | Yes | **8** | **Fair** |
| **Liu 2017** | Yes | Yes | Yes | NR | NR | Yes | Yes | Yes | NR | NR | Yes | NR | Yes | Yes | **9** | **Fair** |
| **Liu 2019** | Yes | Yes | Yes | NR | NR | Yes | Yes | Yes | NR | NR | NR | NR | Yes | Yes | **8** | **Fair** |
| **Lodder 2010** | Yes | Yes | Yes | NR | NR | Yes | Yes | Yes | NR | NR | Yes | NR | Yes | Yes | **9** | **Fair** |
| **Loganathan 2016** | Yes | Yes | Yes | NR | NR | Yes | Yes | Yes | NR | NR | Yes | NR | Yes | Yes | **9** | **Fair** |
| **Lu 2022** | Yes | Yes | Yes | NR | Yes | Yes | Yes | Yes | NR | NR | Yes | NR | Yes | Yes | **10** | **Fair** |
| **Luksic 2016** | Yes | Yes | Yes | Yes | NR | Yes | Yes | Yes | Yes | NR | Yes | NR | Yes | Yes | **11** | **Good** |
| **Madana 2015** | Yes | Yes | Yes | NR | NR | Yes | Yes | Yes | Yes | NR | Yes | NR | Yes | Yes | **11** | **Good** |
| **Mafra 2018** | Yes | Yes | Yes | NR | NR | Yes | Yes | Yes | Yes | NR | Yes | Yes | Yes | Yes | **11** | **Good** |
| **Mair 2018** | Yes | Yes | Yes | Yes | Yes | Yes | Yes | Yes | NR | NR | Yes | NR | Yes | Yes | **11** | **Good** |
| **Manjula 2014** | Yes | Yes | Yes | NR | NR | Yes | Yes | Yes | Yes | NR | Yes | NR | Yes | Yes | **10** | **Fair** |
| **Marinelli 2020** | Yes | Yes | Yes | Yes | NR | Yes | Yes | Yes | Yes | NR | Yes | NR | Yes | Yes | **11** | **Good** |
| **Marktaylor 2010** | Yes | Yes | Yes | Yes | NR | Yes | Yes | Yes | Yes | NR | Yes | Yes | Yes | Yes | **12** | **Good** |
| **Marzouki 2023** | Yes | Yes | Yes | Yes | NR | Yes | Yes | Yes | Yes | NR | NR | NR | Yes | Yes | **10** | **Fair** |
| **Mascitti 2020** | Yes | Yes | Yes | NR | NR | Yes | Yes | Yes | Yes | NR | NR | Yes | Yes | Yes | **10** | **Fair** |
| **Matos 2014** | Yes | Yes | Yes | NR | NR | Yes | Yes | Yes | Yes | NR | Yes | NR | Yes | Yes | **10** | **Fair** |
| **Matsui 2015** | Yes | Yes | Yes | NR | NR | Yes | Yes | Yes | Yes | NR | Yes | NR | Yes | Yes | **10** | **Fair** |
| **Matsushita 2015** | Yes | Yes | Yes | NR | NR | Yes | Yes | Yes | Yes | NR | Yes | NR | Yes | Yes | **10** | **Fair** |
| **Melchers 2012** | Yes | Yes | Yes | NR | NR | Yes | Yes | Yes | Yes | NR | Yes | NR | Yes | Yes | **10** | **Fair** |
| **Michikawa 2012** | Yes | Yes | Yes | Yes | NR | Yes | Yes | Yes | Yes | NR | Yes | NR | Yes | Yes | **11** | **Good** |
| **Mijatov 2023** | Yes | Yes | Yes | Yes | NR | Yes | Yes | Yes | Yes | NR | Yes | NR | Yes | Yes | **11** | **Good** |
| **Mneimneh 2021** | Yes | Yes | Yes | NR | NR | Yes | Yes | Yes | Yes | NR | NR | NR | Yes | Yes | **9** | **Fair** |
| **Morand 2019** | Yes | Yes | Yes | NR | NR | Yes | Yes | Yes | Yes | NR | Yes | NR | Yes | Yes | **10** | **Fair** |
| **Morimoto 2006** | Yes | Yes | Yes | NR | NR | Yes | Yes | Yes | Yes | NR | Yes | NR | Yes | NR | **9** | **Fair** |
| **Mucke 2011** | Yes | Yes | Yes | Yes | NR | Yes | Yes | Yes | Yes | NR | Yes | NR | Yes | Yes | **11** | **Good** |
| **Mücke 2016** | Yes | Yes | Yes | Yes | Yes | Yes | Yes | Yes | Yes | NR | Yes | NR | Yes | Yes | **12** | **Good** |
| **Muhammad 2021** | Yes | Yes | Yes | Yes | Yes | Yes | Yes | Yes | Yes | NR | Yes | Yes | Yes | Yes | **12** | **Good** |
| **Muttagi 2016** | Yes | Yes | Yes | NR | NR | Yes | Yes | Yes | Yes | NR | Yes | NR | Yes | Yes | **10** | **Fair** |
| **Naha 2023** | Yes | Yes | Yes | Yes | NR | Yes | Yes | Yes | Yes | NR | Yes | NR | Yes | Yes | **11** | **Good** |
| **Nair 2018** | Yes | Yes | Yes | Yes | Yes | Yes | Yes | Yes | Yes | NR | NR | NR | Yes | Yes | **11** | **Good** |
| **Nair 2021** | Yes | Yes | Yes | NR | NR | Yes | Yes | Yes | Yes | NR | Yes | NR | Yes | NR | **9** | **Fair** |
| **Nayanar 2019** | Yes | Yes | Yes | NR | NR | Yes | Yes | Yes | Yes | NR | Yes | NR | Yes | Yes | **10** | **Fair** |
| **Nguyen 2021** | Yes | Yes | Yes | NR | NR | Yes | Yes | Yes | Yes | NR | NR | NR | Yes | Yes | **9** | **Fair** |
| **Niu 2016** | Yes | Yes | Yes | NR | NR | Yes | Yes | Yes | Yes | NR | Yes | NR | Yes | Yes | **10** | **Fair** |
| **Noda 2022** | Yes | Yes | Yes | NR | NR | Yes | Yes | Yes | Yes | NR | Yes | NR | Yes | Yes | **10** | **Fair** |
| **Nomura 2009** | Yes | Yes | Yes | NR | NR | Yes | Yes | Yes | Yes | NR | Yes | NR | Yes | Yes | **10** | **Fair** |
| **Nseir 2019** | Yes | Yes | Yes | Yes | NR | Yes | Yes | Yes | NR | NR | NR | NR | Yes | Yes | **9** | **Fair** |
| **O’Brien 2003** | Yes | Yes | Yes | NR | NR | Yes | Yes | Yes | Yes | NR | NR | NR | Yes | Yes | **9** | **Fair** |
| **O-charoenrat 2003** | Yes | Yes | Yes | NR | NR | Yes | Yes | Yes | Yes | NR | Yes | NR | Yes | Yes | **10** | **Fair** |
| **Okada 2003** | Yes | Yes | Yes | NR | NR | Yes | Yes | Yes | Yes | NR | NR | NR | Yes | NR | **7** | **Poor** |
| **Okada 2010** | Yes | Yes | Yes | NR | NR | Yes | Yes | Yes | Yes | NR | Yes | NR | Yes | Yes | **10** | **Fair** |
| **Okuyama 2018** | Yes | Yes | Yes | NR | NR | Yes | Yes | Yes | Yes | NR | NR | NR | Yes | Yes | **9** | **Fair** |
| **Oneyama 2009** | Yes | Yes | Yes | NR | NR | Yes | Yes | Yes | Yes | NR | Yes | NR | Yes | Yes | **10** | **Fair** |
| **Ong 2015** | Yes | Yes | Yes | Yes | NR | Yes | Yes | Yes | Yes | NR | Yes | NR | Yes | Yes | **11** | **Good** |
| **Pandit 2023** | Yes | Yes | Yes | Yes | Yes | Yes | Yes | Yes | Yes | NR | NR | NR | Yes | Yes | **11** | **Good** |
| **Patel 2009** | Yes | Yes | Yes | Yes | Yes | Yes | Yes | Yes | NR | NR | NR | NR | Yes | Yes | **10** | **Fair** |
| **Pedersen 2015** | Yes | Yes | Yes | Yes | Yes | Yes | Yes | Yes | Yes | NR | NR | NR | Yes | Yes | **11** | **Good** |
| **Perisanidis 2013** | Yes | Yes | Yes | NR | NR | Yes | Yes | Yes | Yes | NR | NR | NR | Yes | Yes | **9** | **Fair** |
| **Petrovic 2016** | Yes | Yes | Yes | Yes | NR | Yes | Yes | Yes | Yes | NR | Yes | NR | Yes | Yes | **11** | **Good** |
| **Monevska 2013** | Yes | Yes | Yes | NR | NR | Yes | Yes | Yes | Yes | NR | Yes | NR | Yes | Yes | **10** | **Fair** |
| **Rahman 2021** | Yes | Yes | Yes | NR | NR | Yes | Yes | Yes | Yes | NR | Yes | Yes | Yes | Yes | **11** | **Good** |
| **Sowmya 2020** | Yes | Yes | Yes | NR | NR | Yes | Yes | Yes | Yes | NR | NR | NR | Yes | Yes | **9** | **Fair** |
| **Reddy 2018** | Yes | Yes | Yes | NR | NR | Yes | Yes | Yes | Yes | NR | Yes | NR | Yes | Yes | **10** | **Fair** |
| **Rhutso 2022** | Yes | Yes | Yes | NR | NR | Yes | Yes | Yes | Yes | NR | Yes | Yes | Yes | Yes | **11** | **Good** |
| **Rocchetti 2020** | Yes | Yes | Yes | NR | NR | Yes | Yes | Yes | Yes | NR | Yes | NR | Yes | Yes | **10** | **Fair** |
| **Safi 2017** | Yes | Yes | Yes | Yes | Yes | Yes | Yes | Yes | Yes | NR | Yes | NR | Yes | Yes | **12** | **Good** |
| **Safi 2018** | Yes | Yes | Yes | NR | NR | Yes | Yes | Yes | Yes | NR | Yes | NR | Yes | Yes | **10** | **Fair** |
| **Sagowski 2004** | Yes | Yes | Yes | Yes | NR | Yes | Yes | Yes | Yes | NR | Yes | NR | Yes | NR | **10** | **Fair** |
| **Sahoo 2019** | Yes | Yes | Yes | NR | NR | Yes | Yes | Yes | Yes | NR | Yes | NR | Yes | Yes | **10** | **Fair** |
| **Sakamoto 2016** | Yes | Yes | Yes | NR | NR | Yes | Yes | Yes | NR | NR | NR | NR | Yes | Yes | **8** | **Fair** |
| **Sakata 2018** | Yes | Yes | Yes | NR | NR | Yes | Yes | Yes | Yes | NR | Yes | NR | Yes | Yes | **10** | **Fair** |
| **Salama 2019** | Yes | Yes | Yes | NR | NR | Yes | Yes | Yes | Yes | NR | NR | NR | Yes | Yes | **9** | **Fair** |
| **Sarioglu 2010** | Yes | Yes | Yes | NR | NR | Yes | Yes | Yes | Yes | NR | NR | NR | Yes | Yes | **9** | **Fair** |
| **Sawazaki-calone 2015** | Yes | Yes | Yes | NR | NR | Yes | Yes | Yes | Yes | NR | NR | Yes | Yes | Yes | **10** | **Fair** |
| **Seki 2016** | Yes | Yes | Yes | NR | NR | Yes | Yes | Yes | Yes | NR | NR | NR | Yes | Yes | **9** | **Fair** |
| **Sekikawa 2019** | Yes | Yes | Yes | NR | NR | Yes | Yes | Yes | Yes | NR | Yes | NR | Yes | Yes | **10** | **Fair** |
| **Shan 2020** | Yes | Yes | Yes | Yes | NR | Yes | Yes | Yes | Yes | NR | NR | Yes | Yes | Yes | **11** | **Good** |
| **Sharma 2017** | Yes | Yes | Yes | NR | NR | Yes | Yes | Yes | Yes | NR | NR | NR | Yes | Yes | **9** | **Fair** |
| **Shen 2013** | Yes | Yes | Yes | NR | NR | Yes | Yes | Yes | Yes | NR | NR | NR | Yes | Yes | **9** | **Fair** |
| **Shetty 2020** | Yes | Yes | Yes | NR | NR | Yes | Yes | Yes | Yes | NR | NR | Yes | Yes | Yes | **10** | **Fair** |
| **Shimizu 2018** | Yes | Yes | Yes | NR | NR | Yes | Yes | Yes | Yes | NR | Yes | NR | Yes | Yes | **10** | **Fair** |
| **Shinn 2018** | Yes | Yes | Yes | NR | NR | Yes | Yes | Yes | NR | NR | Yes | NR | Yes | Yes | **9** | **Fair** |
| **Simonetti 2017** | Yes | Yes | Yes | NR | NR | Yes | Yes | Yes | Yes | NR | Yes | Yes | Yes | Yes | **11** | **Good** |
| **Sindhura 2023** | Yes | Yes | Yes | NR | NR | Yes | Yes | Yes | Yes | NR | Yes | NR | Yes | Yes | **10** | **Fair** |
| **Singh 2019** | Yes | Yes | Yes | Yes | Yes | Yes | Yes | NR | Yes | NR | Yes | NR | Yes | Yes | **11** | **Good** |
| **Siriwardena 2018** | Yes | Yes | Yes | Yes | Yes | Yes | Yes | Yes | Yes | NR | NR | NR | Yes | Yes | **11** | **Good** |
| **Solomon 2021** | Yes | Yes | Yes | NR | Yes | Yes | Yes | Yes | Yes | NR | NR | NR | Yes | Yes | **10** | **Fair** |
| **Son 2017** | Yes | Yes | Yes | Yes | NR | Yes | Yes | Yes | Yes | NR | Yes | NR | Yes | Yes | **11** | **Good** |
| **Sparano 2004** | Yes | Yes | Yes | NR | NR | Yes | Yes | Yes | Yes | NR | Yes | NR | Yes | Yes | **10** | **Fair** |
| **Spoerl 2021** | Yes | Yes | Yes | Yes | Yes | Yes | Yes | Yes | Yes | NR | NR | NR | Yes | Yes | **11** | **Good** |
| **Spoerl 2022** | Yes | Yes | Yes | Yes | Yes | Yes | Yes | Yes | Yes | NR | NR | NR | Yes | Yes | **11** | **Good** |
| **Stoop 2020** | Yes | Yes | Yes | NR | NR | Yes | Yes | Yes | Yes | NR | Yes | NR | Yes | Yes | **10** | **Fair** |
| **Sundaram 202** | Yes | Yes | Yes | NR | NR | Yes | Yes | Yes | Yes | NR | Yes | NR | Yes | Yes | **10** | **Fair** |
| **Suresh 2015** | Yes | Yes | Yes | NR | NR | Yes | Yes | Yes | Yes | NR | Yes | Yes | Yes | Yes | **11** | **Good** |
| **Tai 2013** | Yes | Yes | Yes | NR | NR | Yes | Yes | Yes | Yes | NR | Yes | NR | Yes | Yes | **10** | **Fair** |
| **Tam 2018** | Yes | Yes | Yes | NR | NR | Yes | Yes | Yes | Yes | NR | Yes | Yes | Yes | Yes | **11** | **Good** |
| **Tan 2023** | Yes | Yes | Yes | NR | NR | Yes | Yes | Yes | Yes | NR | Yes | NR | Yes | Yes | **10** | **Fair** |
| **Tarsitano 2016** | Yes | Yes | Yes | Yes | NR | Yes | Yes | Yes | Yes | NR | Yes | Yes | Yes | Yes | **12** | **Good** |
| **Thiagarajan 2014** | Yes | Yes | Yes | NR | Yes | Yes | Yes | Yes | NR | NR | NR | NR | Yes | Yes | **9** | **Fair** |
| **Thompson 1986** | Yes | NR | Yes | NR | NR | Yes | Yes | NR | NR | NR | NR | NR | Yes | NR | **5** | **Poor** |
| **Ting 2021** | Yes | Yes | Yes | NR | Yes | Yes | Yes | Yes | Yes | NR | Yes | Yes | Yes | Yes | **12** | **Good** |
| **Toom 2019** | Yes | Yes | Yes | NR | NR | Yes | Yes | Yes | Yes | NR | Yes | NR | Yes | Yes | **10** | **Fair** |
| **Van Lanschot 2020** | Yes | Yes | Yes | NR | Yes | Yes | Yes | Yes | Yes | NR | Yes | NR | Yes | Yes | **11** | **Good** |
| **Varsha 2015** | Yes | Yes | Yes | NR | NR | Yes | Yes | Yes | Yes | NR | Yes | NR | Yes | NR | **9** | **Fair** |
| **Verma 2021** | Yes | Yes | Yes | NR | NR | Yes | Yes | Yes | Yes | NR | Yes | NR | Yes | Yes | **10** | **Fair** |
| **Vidiri 2019** | Yes | Yes | Yes | NR | NR | Yes | Yes | Yes | Yes | NR | Yes | Yes | Yes | Yes | **11** | **Good** |
| **Wang 2016** | Yes | Yes | Yes | NR | NR | Yes | Yes | Yes | Yes | NR | Yes | NR | Yes | NR | **9** | **Fair** |
| **Wang 2021** | Yes | Yes | Yes | NR | NR | Yes | Yes | Yes | Yes | NR | Yes | NR | Yes | Yes | **10** | **Fair** |
| **Wang 2016 (2)** | Yes | Yes | Yes | Yes | NR | Yes | Yes | Yes | Yes | NR | Yes | NR | Yes | Yes | **11** | **Good** |
| **Warburton 2007** | Yes | Yes | Yes | NR | NR | Yes | Yes | Yes | Yes | NR | NR | NR | Yes | Yes | **9** | **Fair** |
| **Wedemeyer 2013** | Yes | Yes | Yes | NR | NR | Yes | Yes | Yes | Yes | NR | Yes | NR | Yes | Yes | **10** | **Fair** |
| **Wei 2018** | Yes | Yes | Yes | Yes | Yes | Yes | Yes | Yes | Yes | NR | Yes | Yes | Yes | Yes | **12** | **Good** |
| **Weimar 2018** | Yes | Yes | Yes | Yes | Yes | Yes | Yes | Yes | Yes | NR | Yes | NR | Yes | Yes | **11** | **Good** |
| **Woolgar 2003** | Yes | Yes | Yes | Yes | NR | Yes | Yes | Yes | Yes | NR | Yes | NR | Yes | Yes | **11** | **Good** |
| **Wu 2019** | Yes | Yes | Yes | NR | NR | Yes | Yes | Yes | Yes | NR | Yes | NR | Yes | Yes | **10** | **Fair** |
| **Xie 2014** | Yes | Yes | Yes | NR | NR | Yes | Yes | Yes | Yes | NR | Yes | Yes | Yes | Yes | **11** | **Good** |
| **Xie 2019** | Yes | Yes | Yes | NR | NR | Yes | Yes | Yes | Yes | NR | Yes | NR | Yes | Yes | **10** | **Fair** |
| **Xu 2021** | Yes | Yes | Yes | NR | NR | Yes | Yes | Yes | Yes | NR | NR | NR | Yes | Yes | **9** | **Fair** |
| **Yamada 2017** | Yes | Yes | Yes | NR | NR | Yes | Yes | Yes | Yes | NR | Yes | NR | Yes | Yes | **10** | **Fair** |
| **Yamagata 2019** | Yes | Yes | Yes | No | NR | Yes | Yes | Yes | NR | NR | NR | NR | Yes | Yes | **8.5** | **Fair** |
| **Yamakawa 2018** | Yes | Yes | Yes | NR | NR | Yes | Yes | Yes | Yes | NR | Yes | Yes | Yes | Yes | **11** | **Good** |
| **Yamamoto 2014** | Yes | Yes | Yes | NR | NR | Yes | Yes | Yes | NR | NR | NR | Yes | Yes | NR | **8** | **Fair** |
| **Yang 2021** | Yes | Yes | Yes | NR | NR | Yes | Yes | Yes | Yes | NR | Yes | NR | Yes | Yes | **10** | **Fair** |
| **Yoshida 2018** | Yes | Yes | Yes | NR | NR | Yes | Yes | Yes | Yes | NR | NR | NR | Yes | Yes | **9** | **Fair** |
| **Yoshizawa 2022** | Yes | Yes | Yes | NR | NR | Yes | Yes | Yes | Yes | NR | Yes | Yes | Yes | Yes | **11** | **Good** |
| **Yu 2019** | Yes | Yes | Yes | NR | NR | Yes | Yes | Yes | Yes | NR | Yes | Yes | Yes | Yes | **11** | **Good** |
| **Zenga 2019** | Yes | Yes | Yes | NR | Yes | Yes | Yes | Yes | Yes | NR | Yes | NR | Yes | Yes | **11** | **Good** |
| **Zhang 2019** | Yes | Yes | Yes | NR | NR | Yes | Yes | Yes | Yes | NR | Yes | Yes | Yes | Yes | **11** | **Good** |
